# Angiogenic Doping: Plausible Yet Difficult to Detect

**DOI:** 10.1007/s40279-026-02447-y

**Published:** 2026-05-21

**Authors:** Sofie Lehto, Setareh Sima, Jaana Künnapuu, Sergei Iljukov, Michael Jeltsch

**Affiliations:** 1 Drug Research Program, University of Helsinki, Helsinki, Finland; 2 Department of Pharmaceutical Biotechnology, School of Pharmacy and Pharmaceutical Sciences, Isfahan University of Medical Sciences, Isfahan, Iran; 3 Sports Medicine, Terveystalo Healthcare Services, Helsinki, Finland; 4 Individualized Drug Therapy Research Program, University of Helsinki, Helsinki, Finland; 5 Wihuri Research Institute, Helsinki, Finland; 6 Helsinki One Health, Helsinki, Finland; 7 Terveystalo Lahti, Aleksanterinkatu 13, 15110 Lahti, Finland; 8 Drug Research Program, Faculty of Pharmacy, University of Helsinki, Biocenter 2, Viikinkaari 5E, P.O.B. 56, 00014 Helsinki, Finland

## Abstract

In endurance athletes, cardiovascular oxygen delivery is the primary limitation to performance. While the focus of athletes and regulatory bodies has been on hemoglobin and red blood cells, the increased oxygen-delivering capacity resulting from training is also a consequence of the expanded blood volume, which requires vascular adaptation. Angiogenesis—the formation of new blood vessels from pre-existing ones—is an understudied area in exercise physiology due to the need for invasive procedures. Among the vascular endothelial growth factors, VEGF-A and VEGF-D are the most potent angiogenic inducers and thus candidates for doping purposes. VEGF expression can be stimulated by several external factors, of which oxygen deprivation and its mimics are the most significant in the context of doping. A controlled overexpression of VEGF-A or VEGF-D, and the resulting blood vessel formation, could directly increase vascular space and indirectly increase blood volume and athletic ability. A master regulatory gene such as HIF-1α would be a preferred target over any single growth factor, as it would affect red blood cell production and vascular expansion synchronously. Currently available compounds may already be misused, with potential unintended consequences, including the aggravation of inflammatory diseases or tumor progression. Due to the ease of implementation and difficulty of detection, angiogenic doping and possible detection strategies deserve to be studied further.

**Graphical Abstract:**

**Figure:**
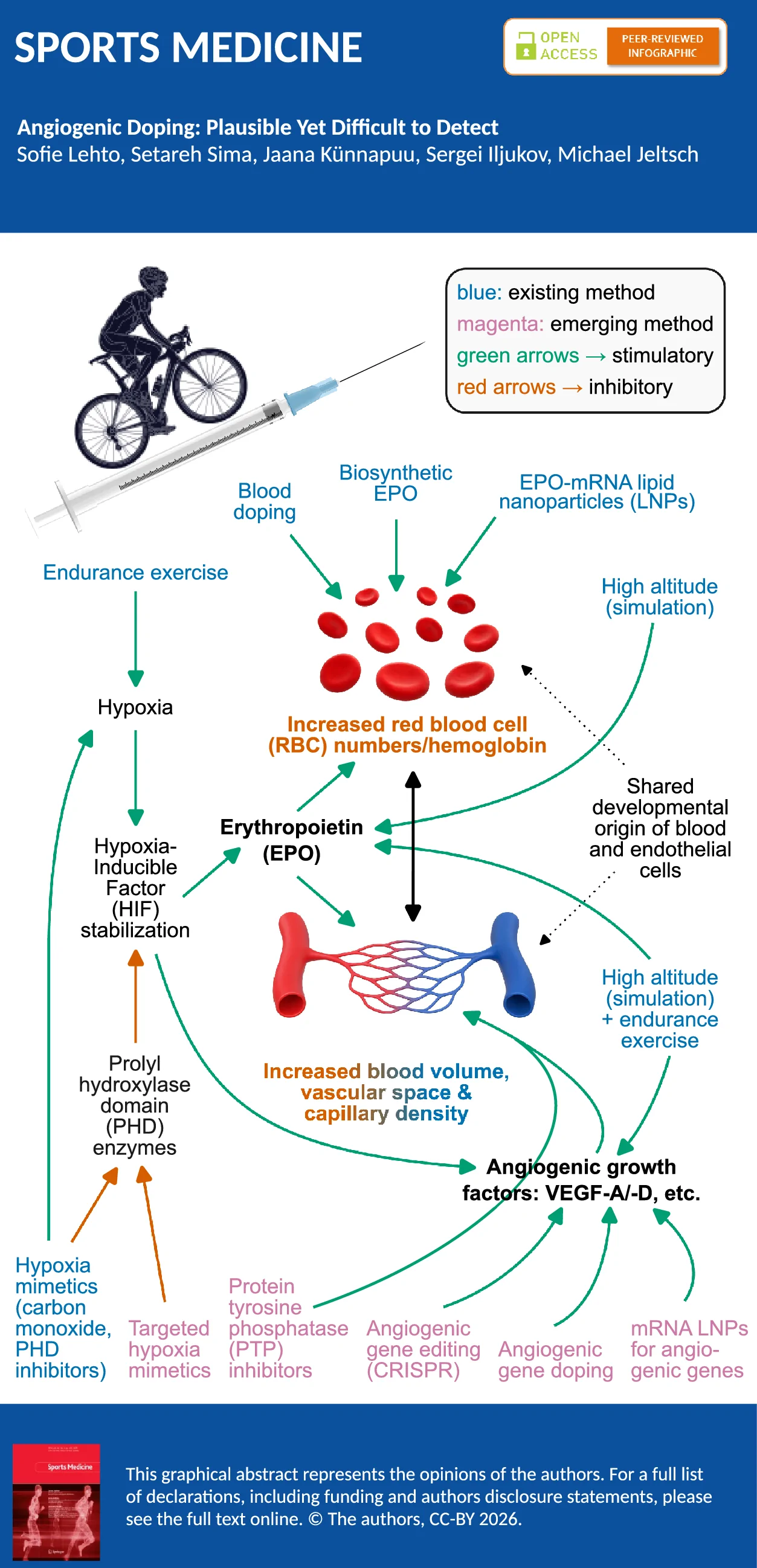

**Supplementary Information:**

The online version contains supplementary material available at 10.1007/s40279-026-02447-y.

## Key Points

**Table:** 

Doping based on new blood vessel formation (‘angiogenic doping’) can likely boost athletic performance and would be difficult to detect.
Angiogenic doping could target growth factors for blood vessels or regulatory proteins. Suitable pharmacologic agents, currently not subject to doping controls, are readily available.
Despite the risk of serious side effects, angiogenic doping is likely already a reality, and its use will increase with the emergence of effective advanced medical procedures such as gene therapy and gene editing.

## Introduction

Until about 20 years ago, with blood doping and erythropoietin, cheating endurance athletes had a head start in the cat-and-mouse game with the World Anti-Doping Agency (WADA). In their efforts, the cardiovascular system and its oxygen-transport capacity have been central targets for manipulation. It is generally believed that the introduction of the Athlete Biological Passport (ABP) has largely kept the use of blood doping and erythropoietin at bay [1]. However, a new front is opening up: *next-generation doping*. We use this term to collectively describe the use of recent advances in medicine, such as gene therapy, gene editing, and targeted drug delivery, for doping purposes. As molecular biology and gene therapy technologies improve, the risk of misuse to enhance athletic performance in both human and non-human animal athletes increases [2–5]. WADA predicted this over 20 years ago, when, in 2003, it added gene doping to its list of prohibited substances and methods, responding to the rapid development in this area [6].

While some form of doping has been used by elite athletes since ancient Greece and Rome [7], doping opportunities have been on the rise, facilitated by the discovery and development of effective drugs to treat diseases. With the arrival of unprecedentedly powerful new drug classes within the last decades (recombinant proteins, targeted drug delivery, gene therapy, and gene editing), doping has become a considerable liability to the integrity of sports [8].

To maximize the blood’s oxygen-carrying capacity, erythropoietin has famously been used to increase the red blood cell (RBC) count, and before that (and perhaps again increasingly) blood doping, which dates back to the early 1970s, when the first successful attempts were, by rumor and admission, made by Finnish long-distance runners [9]. Although there is likely substantial heterogeneity among athletes [10], the major bottleneck in top endurance athletes is believed to be the cardiovascular system’s ability to deliver oxygen (“flow-limited athletes”) [11, 12]. Stimulating red blood cell production with erythropoietin injections has received considerable attention due to its use by prominent cyclists [13]. In the context of endurance sports, public perception and doping control have focused on increases in hemoglobin and RBC density, as well as on concurrent hematological parameters [14, 15]. However, a substantial part of the increased ability to deliver oxygen to muscles that results from endurance training is attributed to an increase in total blood volume [16]. In line with this, the total circulating mass of hemoglobin displays a stronger relationship with maximum oxygen uptake than hemoglobin concentration or blood volume alone [16–18].

Endurance training increases red blood cell production. Yet, the majority of this increase is not visible as an increase in hemoglobin or RBC concentration, but rather is absorbed by the larger blood volume. An increase in blood volume inevitably requires an expansion of the intravascular compartment (the fluid-filled space within blood vessels) to accommodate the blood. Both blood composition and blood volume have been extensively researched, but the changes to the vascular space itself have been much less studied. The scarcity of studies in humans is not surprising since the study of blood vessels in skeletal muscles requires repeated invasive procedures that are prohibitive for competitive athletes. While erythropoietin acts primarily on the RBC production, prolyl hydroxylase domain (PHD) inhibitors induce a broader hypoxia-responsive transcriptional program. In addition to increasing erythropoietin expression, they upregulate genes of the VEGF family, most notably *VEGFA*, the central angiogenic growth factor. VEGF-A plays a key role in regulating capillary density by stimulating endothelial cell proliferation and migration. Endothelial cells form the inner lining of all blood vessels and constitute the critical interface between circulating blood and surrounding tissues [19]. Also, erythropoietin itself exerts effects beyond RBC formation, including direct actions on the vascular system [20]. However, to date, none of the published human clinical studies has examined the effect of PHD inhibitors on total blood volume or capillary density.

When increasing the total blood volume by transfusion, the body rapidly normalizes hematological parameters [21]. Although blood doping has repeatedly been shown to be effective (reviewed by [22]), the basis of this enhanced performance remains unclear [21]. The performance gains do not only result from changes in blood composition, but the blood vessels themselves also play a critical role, as their collective internal volume imposes constraints on the maximal blood volume, which limits in turn red blood cell numbers. Hence, the factors that regulate blood vessel growth—notably the VEGFs—have become particularly interesting in efforts to increase exercise performance, not least because they and their effects are relatively difficult to detect.

**Fig. 1:**
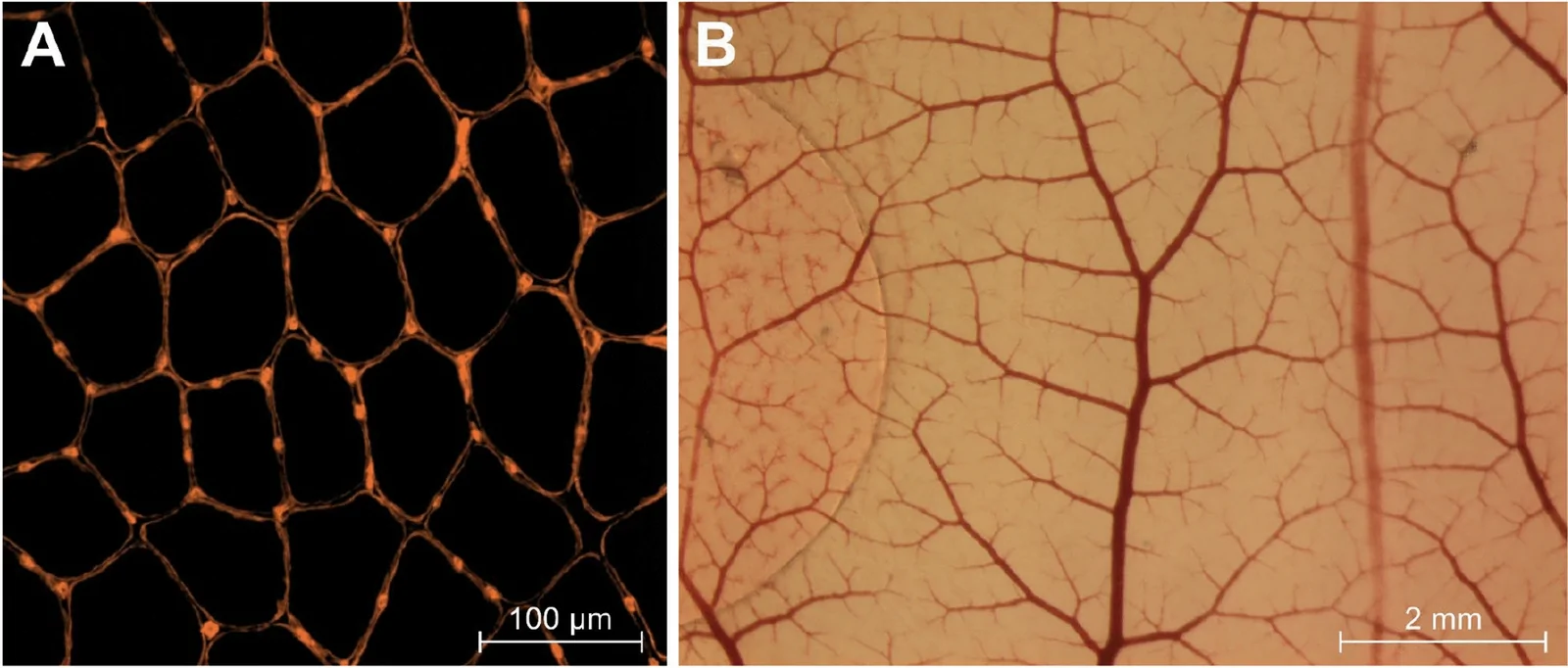
Increasing oxygen availability has been a central goal of training and doping. For doping purposes, angiogenesis—the growth of new blood vessels—has received less attention than the composition of the blood itself. **A** Laminin staining in a cross-section of human skeletal muscle shows the basement membrane surrounding each myofiber and capillary. The partial pressure gradient of oxygen between the capillary and the mitochondria, together with the diffusion distance, is a primary determinant of the rate of oxygen flux in the terminal diffusion pathway. Image CC-licensed from [23]. **B** Angiogenic growth factors which mediate the physiological increase in vascular density resulting from training, can also be applied exogenously. To assess the angiogenic effect of VEGF-A, the disc on the left was coated with VEGF-A and applied to the chorioallantoic membrane (CAM) of a developing chicken, resulting in a denser vascular network and more angiogenic sprouts. The CAM assay is frequently used to test the angiogenic potential of compounds [24, 25]. Analogous to sports performance, the CAM’s ability to deliver oxygen is a limiting factor for the speed of embryonic development [26]

The vascular system is essential for athletic performance as it delivers the oxygen and nutrients needed for sustained exercise while removing CO_2_ and waste products. Endurance performance is primarily limited by the maximal rate of oxygen uptake (V̇O_2_max), which is governed by a series of convective and diffusive resistances. The convective pathway is defined by maximal cardiac output and arterial oxygen content, ensuring bulk delivery to the microcirculation. The diffusive pathway is determined by the oxygen pressure gradient between the capillary and the mitochondria and the muscle's diffusing capacity (see Fig. 1) [27].

Enhancing capillary density through angiogenesis is a fundamental physiological adaptation to exercise training because it increases the mean transit time of red blood cells and expands the surface area for exchange. A higher capillary density reduces diffusion distances and prevents the ‘functional shunting’ of oxygen at high flow rates, thereby optimizing the microenvironment for oxidative metabolism and elevating the ceiling for aerobically generated power [28].

Targeting oxygen transport has been proven to be a successful doping strategy in the past. The use of erythropoietin among cyclists was largely unchecked before WADA began testing in 2000, after piloting testing schemes since 1996. Still, despite testing, elaborate techniques were deployed to evade detection [29]. For example, erythropoietin was combined with intravenously administered volume expanders, such as hydroxyethyl starch (HES), which has been marketed under trade names such as Voluven^®^ and is included on the WADA Prohibited List [30], to lower otherwise suspiciously elevated hemoglobin levels [31]. Manipulating VEGFs in a controlled manner to expand the blood volume the cardiovascular system can hold could increase its oxygen-carrying capacity without increasing hemoglobin levels or RBC concentration [8].

This narrative review examines angiogenic doping as an emerging threat to sports integrity. The aims are to (1) evaluate the biological basis for angiogenesis-based performance enhancement, focusing on VEGF family growth factors as the primary and direct regulators of angiogenesis and their transcriptional activators; (2) assess the feasibility and accessibility of such angiogenic doping methods, from pharmacological agents to gene therapy and gene editing; and (3) analyze current detection challenges and potential countermeasures. An initial systematic literature search (see Supplemental Fig. 1 in the electronic supplementary material [ESM]) revealed that angiogenic doping remains an understudied area. The initial draft was therefore enriched with expert contributions to corroborate the findings with evidence from vascular and molecular biology, exercise physiology, clinical trials, and anti-doping research. Unless explicitly specified otherwise or obvious from the context, the data referenced in this review are derived from human studies. In Sects. 2 through 3.1, we only indicate exceptions to the rule of thumb that basic biomedical research is performed in rodents if there are reasons to believe that significant species differences might exist.

## The Vascular Endothelial Growth Factor (VEGF) Family

The vascular endothelial growth factors (VEGFs) are potential targets for *next-generation doping* due to their blood vessel growth-inducing (angiogenic) effects. The VEGF family consists of VEGF-A, VEGF-B, VEGF-C, VEGF-D, VEGF-E, VEGF-F, and placenta growth factor (PlGF), each encoded by its own gene [32, 33]. Of these, VEGF-A through VEGF-D and PlGF are endogenous to humans (see Table 1). VEGF-A (often simply called VEGF in older literature) is the primary angiogenic factor [32], and its potential for gene doping has been previously recognized [34–38].

**Table 1:** Biological effects of VEGFs

	Interacting receptors			Biological effects			References First description(s)/recent review(s)
				Angiogenesis	Vascular permeability	Lymphangiogenesis	
	VEGFR-1	VEGFR-2	VEGFR-3				
VEGF-A	+	+		+++	++	Conflicting reports^1^	[53, 54] / [55, 56]
PlGF	+			Indirectly^2^	–	Not determined	[57] / [58]
VEGF-B	+			Conflicting reports^3^	–	–	[59] / [56]
VEGF-C		+	+	( +)^4^	+	+++	[60, 61] / [62, 63]
VEGF-D		+	+	(+++)^4^	+	++	[64–66] / [67, 68]
VEGF-E^5^		+		+	++	Not determined	[69] / [56]
VEGF-F^5^		+		+	+++	Not determined	[70] / [71]

*PlGF* placenta growth factor, *VEGF* vascular endothelial growth factor, *VEGFR* vascular endothelial growth factor receptor

^1^VEGF-A can be lymphangiogenic [72, 73], but the effect is likely indirect (blood vessels produce VEGF-C, interstitial pressure renders VEGFR-3 more sensitive to VEGF-C, inflammation upregulates VEGF-C)

^2^PlGF competes with VEGF-A for binding to VEGFR-1, thus pushing VEGF-A onto the pro-angiogenic VEGFR-2 [74]

^3^The angiogenic effect of VEGF-B seems to be species- and heart-specific [75]. It is unclear whether the effect is direct or whether VEGF-B leads to hypertrophy, which in turn stimulates angiogenesis [75, 76]

^4^Both VEGF-C and VEGF-D can be angiogenic but only after processing by specific proteases [55, 77, 78]. This processing increases their affinity for VEGFR-2. In the case of VEGF-D, the growth factor simultaneously loses its affinity for VEGFR-3, making it the only endogenous growth factor that binds exclusively to VEGFR-2, which explains its strong angiogenic potential, observed e.g. in rabbit and pig studies [79, 80]

^5^VEGF-E and VEGF-F are not present in mammals. VEGF-Es are viral homologs, and VEGF-Fs are homologs found primarily in snake venom

VEGF concentrations are highest during embryogenesis and fetal development, where, for example, VEGF-A is first essential for the survival of hemangioblasts, the common developmental ancestors of blood and endothelial cells [39–41], and subsequently for the growth and development of the cardiovascular system [42, 43]. The close relationship between blood and endothelial cells is maintained into adulthood through many shared cell surface receptors [44, 45].

VEGF-C is primarily known for its role in the development and growth of the lymphatic system [46–48] and in cancer metastasis via lymphatic dissemination [49–51]. In adults, VEGF-C regulates lymphangiogenesis during tissue regeneration, such as wound healing [52].

The other human VEGFs have more specialized roles. VEGF-B is prominently expressed during early embryonic development, acting in a species-specific manner in heart vascularization and as a general vascular survival factor [75, 76, 81, 82]. Unlike VEGF-C, which is primarily lymphangiogenic, VEGF-D can also be a potent angiogenic inducer, sometimes even outperforming VEGF-A [79]. In humans, it is believed to replace VEGF-A and thus allow tumors to resist drugs that target VEGF-A [83, 84].

PlGF, named for its high expression in the placenta [57], is vital for the endometrial cycle and fetal development. Outside the reproductive system, it indirectly boosts angiogenesis and vascular permeability by displacing VEGF-A from the inhibitory VEGFR-1, allowing VEGF-A to bind to the mitogenic receptor VEGFR-2 [74, 85, 86]. In mice, PlGF, VEGF-B, and VEGF-D contribute to, but are not essential for, angiogenesis, as studies have shown only minor changes in the cardiovascular system when each gene was deleted individually [87–94].

### The Regulation of VEGF Expression

**Fig. 2:**
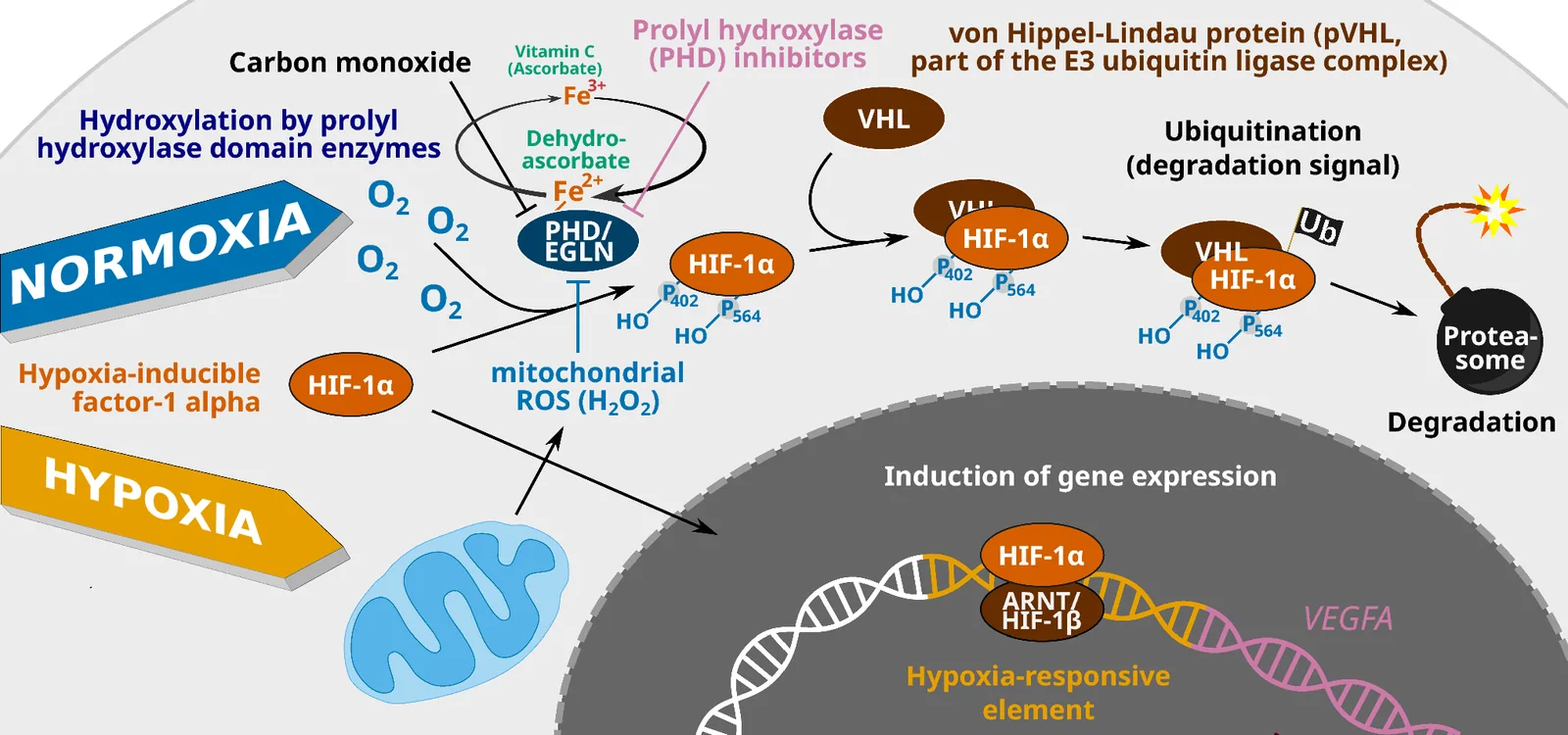
Induction of VEGF-A expression by HIF-1α. Prolyl hydroxylases limit the physiological activation of HIF-1α, the oxygen-sensitive subunit of the HIF-1 complex, and are central enzymes targeted by many interventions aimed at inducing hypoxia-responsive genes. Fe^2+^ in the active site of PHDs participates in the prolyl hydroxylation as a cofactor, being oxidized to Fe^3+^ (the same mechanism underlies the connective tissue phenotype in scurvy, where ascorbate deficiency prevents the reduction of Fe^3+^ to Fe^2+^ and thus the prolyl hydroxylation required for collagen maturation). Carbon monoxide is not only a toxic gas, but also an endogenously produced gasotransmitter. It is generated when heme oxygenases-1 and -2 degrade heme. Heme oxygenase-1 is strongly upregulated by hypoxia, and the generated CO further enhances the expression of hypoxia-response genes, such as *VEGFA*. Cytochrome c oxidase (Complex IV) is a heme-containing enzyme in the mitochondrial electron transport chain. Under hypoxia, reduced cytochrome c oxidase activity leads to ROS generation and HIF-1 stabilization [121]. *ARNT* Aryl hydrocarbon receptor nuclear translocator, *HIF* hypoxia-inducible factor, *P* proline, *PHD/EGLN* prolyl hydroxylase domain enzyme/egg-laying defective gene 9, *ROS* reactive oxygen species, *Ub* ubiquitin, *VEGF* vascular endothelial growth factor, *VHL* von Hippel-Lindau protein

VEGF-A is regulated by a complex array of external factors at multiple levels, from gene transcription to mRNA translation [95–98]. In adults, VEGF-A levels are typically low in most organs, but are increased during wound healing [99, 100], muscle growth [101, 102], tissue repair [103], and the hair growth [104] and female reproductive cycles [105, 106], for example, but also during inflammation [107–109] (reviewed by [56]) and in pathologically oxygen-depleted tissues, such as tumors (reviewed by [110]). There is a rich literature about hypoxia-induced VEGF-A expression (reviewed by [111]). In contrast, the evidence for hypoxia-induced upregulation of other VEGF family members is either negative [112] or sparse, and often limited to pathological situations [113, 114]. Upregulation of VEGF-A expression is mediated by hypoxia-inducible factor-1 (HIF-1) [115, 116] (see Fig. 2). Under hypoxic conditions, HIF-1 degradation is suppressed, allowing it to move into the nucleus. There, it acts as a transcription factor interacting with hypoxia-responsive elements in hypoxia-inducible genes, including *EPO* and *VEGFA* [115, 117–120]. One goal of this hypoxia-induced change in gene expression is to improve oxygen transport to hypoxic tissue by increasing vascularization. Besides hypoxia, other factors and variables, such as growth factors, inflammatory cytokines, and an acidic pH, can increase HIF-1 levels and, therefore, VEGF-A expression [97].

### The VEGF Receptors

**Fig. 3:**
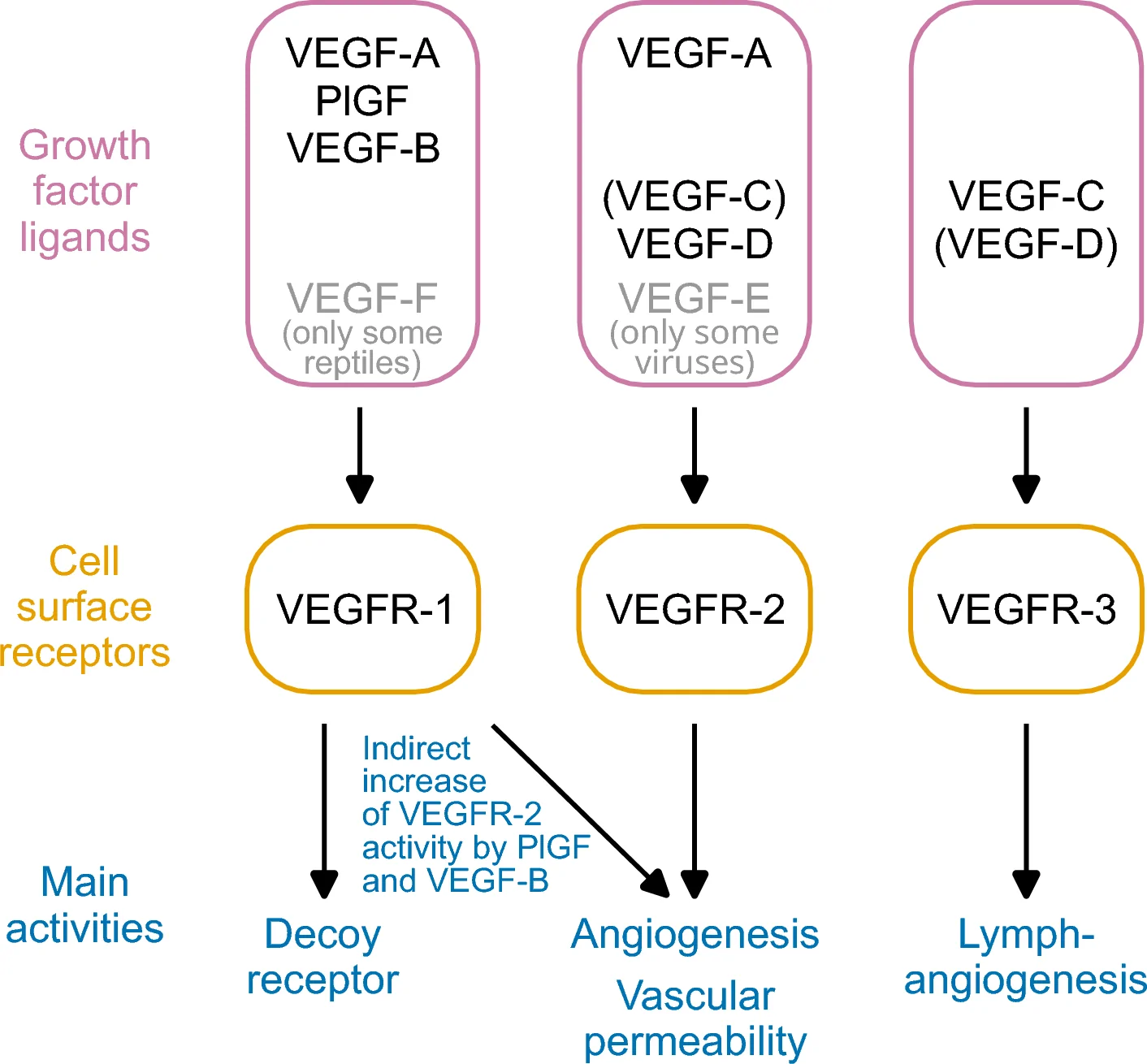
The VEGFs and VEGF receptors. Humans feature five endogenous VEGF growth factors, each of which interacts with a specific set of receptors, resulting in distinct biological effects. As elaborated in Sect. 4.6, VEGF-A and VEGF-D are the most promising targets for therapy and performance enhancement. Despite its ability to interact with VEGFR-2, in vivo application of VEGF-C is surprisingly specific for the lymphatic system, perhaps due to its unique activation by ADAMTS-3/14 [137, 138]. VEGF-D, on the other hand, can lose all of its affinity for VEGFR-3—and thus its lymphangiogenic potency—if activated by cathepsin D [139, 140]. *VEGF* vascular endothelial growth factor, *VEGFR* vascular endothelial growth factor receptor, *PlGF* placenta growth factor

VEGFs bind to VEGF receptors (VEGFRs), which are tyrosine kinase receptors [40]. Placental mammals such as humans and mice feature three VEGFRs (VEGFR-1, VEGFR-2, and VEGFR-3), while marsupial mammals feature one more (VEGFR-4, aka Kdr-like). The different receptors mediate distinct effects (Fig. 3). VEGFR-2 is the best-known of these, as it mediates most of the known responses to VEGF-A, including angiogenesis and vascular permeability [122–124]. VEGFR-1 is thought to limit angiogenesis by trapping VEGF-A and thus decreasing signaling via the angiogenic VEGFR-2 [125, 126]. VEGFR-1 also mediates some of the disease-related signaling of VEGFs [127]. VEGFR-3, on the other hand, mediates the effects of VEGF-C and VEGF-D on the lymphatic system, as these receptors are found mainly on lymphatic endothelial cells [128, 129]. All VEGFRs are primarily found on the surface of endothelial cells [130], but there are numerous exceptions, including immune cells [45, 129, 131], vascular smooth muscle cells [132, 133], and neurons [134–136].

## VEGF-A: the Prototypical Angiogenic Growth Factor

VEGF-A, discovered by Senger et al. in 1983 [53], is the most critical promoter of blood vessel growth, during both normal human development and abnormal pathological processes [40]. VEGF-A stimulates the growth and movement of endothelial cells and inhibits their apoptosis [32, 54, 141, 142]. Its expression can be induced in a wide variety of cell types and tissues [143], including muscle fibers [144, 145], myofibroblasts [146], endothelial cells [119, 147], and vascular smooth muscle cells [132]. The VEGF-A gene generates several isoforms by alternative mRNA splicing, each with slightly different effects on the vascular system [32, 127, 148]. VEGF-A expression requires tight control during embryogenesis [42, 43] and in postnatal life [149, 150].

VEGF-A plays an essential role not only in angiogenesis but also in vasculogenesis (the embryonic de novo formation of blood vessels) [151] and embryonic hematopoiesis [41], for which it remains important in adult life by protecting the hematopoietic stem cells from apoptosis [171, 172]. VEGF-A also increases vascular permeability and vasodilation [53, 152], effects linked to pathological conditions in which VEGF-A is overexpressed [40], though their physiological relevance remains uncertain.

### The Pathogenic Roles of VEGF-A

**Fig. 4:**
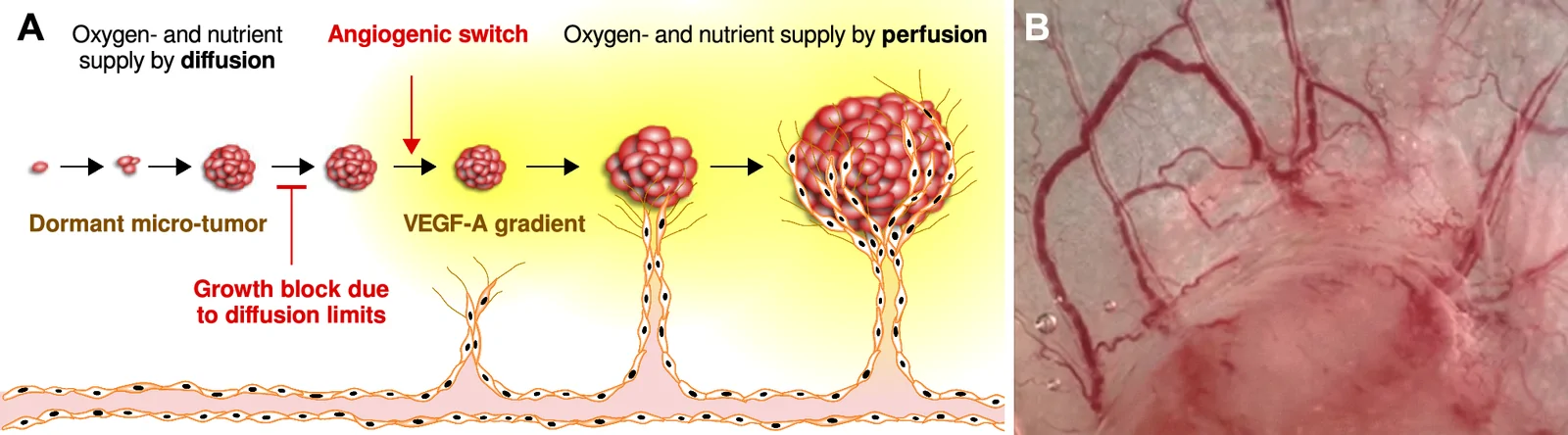
A potential consequence of angiogenic doping is to promote the growth of otherwise clinically dormant micro-tumors. **A** The original concept of tumor blood vessel dependency [162] was supported by experimental evidence, including the discovery of VEGF-A [53, 54] and the angiogenic switch [163, 164]. **B** Tumors also demonstrate that VEGF-A alone does not create a functional, hierarchical network. VEGF-A expression by this mouse ovarian tumor resulted in higher capillary density, but did not produce a hierarchical network optimized for oxygen delivery (see also [24, 25]); image CC-licensed from [165]. *VEGF* vascular endothelial growth factor

Because blood vessels penetrate virtually all tissues, VEGF-A is integral to many pathological processes [56]. VEGF-A enables tumor growth (Fig. 4A) and metastasis [153, 154]. VEGF-A also drives pathological angiogenesis in neovascular eye diseases, such as diabetic retinopathy [155, 156], and inflammatory diseases, such as rheumatoid arthritis or osteoarthritis [157, 158]. In atherosclerosis, rather than stimulating beneficial neovessel formation, VEGF-A seems to contribute to plaque buildup by promoting foam cell formation [159, 160]. However, unregulated, pathological VEGF-A expression produces irregular, leaky, dysfunctional vessels (Fig. 4B) [161], and anti-VEGF-A therapies have improved the therapeutic outcomes in several neovascular diseases, in some cases even dramatically [110, 161]. Conversely, VEGF-A doping (as well as pro-angiogenic therapy for heart diseases) has the potential to trigger or exacerbate the same diseases in which antiangiogenic therapy is beneficial.

### The Role of VEGF-A in Increasing Sports Performance

There is ample evidence from both animal and human studies for the role of VEGF-A in the vascular response to endurance training (reviewed by [166, 167]). In a study by Richardson et al., the amount of VEGF-A mRNA in skeletal muscle increased more than tenfold in untrained human subjects within 1 h after endurance exercise, while the response in trained subjects was less pronounced. Muscle capillarization increased on average by 18% after 8 weeks of training, but the capillary density remained largely the same as the muscle fiber area increased to a similar degree [102]. However, angiogenesis is not governed by VEGF-A alone, but rather by a dynamic balance of pro- and antiangiogenic factors. While VEGF-A is the primary direct driver, some of the capillary response persisted even when VEGFR-2 was blocked in a rat exercise model [168], indicating that other pro-angiogenic players contribute. For instance, in both human and animal studies, endurance exercise upregulated Ang-2 and downregulated Ang-1 [168–171], destabilizing the vessel and thereby permitting endothelial cells to proliferate and migrate in response to VEGF-A. For a review that discusses non-VEGF angiogenic factors, see [172].

Key physical stimuli that upregulate VEGF-A in skeletal muscle include mechanical forces, such as increased hemodynamic shear stress on the capillary endothelium from elevated blood flow, and passive stretch and compression of the vascular network during muscle contractions [173, 174]. Both VEGFR-2 and VEGFR-3 are part of mechanosensing complexes [175, 176]. The same forces also upregulate endothelial nitric oxide synthase (eNOS), a key enzyme whose product, nitric oxide (NO), is itself a potent stimulator of VEGF-A expression [166, 177]. The relative hypoxia experienced by muscle fibers during intense exercise activates HIF-1α [169, 178]. This process is further modulated by the transcriptional coactivator PGC-1α, which coordinates mitochondrial biogenesis with angiogenesis [179]. However, the association between HIF-1α and VEGF-A mRNA expression in endurance-trained skeletal muscle is surprisingly weak [180], and HIF-1α knockout (KO)-mice show no significant difference in VEGF-A mRNA response to acute exercise compared with wild-type mice. Even more surprisingly, HIF-1α KO mice exhibit greater basal capillarization than wild-type littermates [181]. Lindholm and Rundqvist [182] proposed that while acute exercise activates HIF-1, long-term endurance training blunts the HIF-1α response by inducing its negative regulators, most notably the PHD enzymes and factor inhibiting HIF-1 (FIH), and a similar blunting has also been seen in the heart muscle of mice as a response to prolonged training [183]. Hence, the attention has shifted to HIF-1-independent pathways. For example, metabolic byproducts of exercise, such as adenosine and lactate, have been identified as important chemical signals that promote VEGF-A expression and secretion [184–186].

Beyond gene expression, the local bioavailability of VEGF-A protein has been shown to be critical in several human studies. VEGF-A levels in the abluminal, muscle interstitium increased several-fold during an exercise bout [187, 188] (see also Sect. 5.5 for why blood VEGF-A levels are not relevant). This VEGF-A release is mediated, at least in part, by adenosine acting on A_2B_ receptors on muscle cells [184]. During endurance training, while the acute exercise-induced increase in VEGF mRNA is attenuated, the basal protein level of VEGF-A within the muscle tissue is often elevated. This adaptation ensures that a readily available pool of VEGF-A is stored and can be rapidly secreted in response to subsequent exercise bouts, thereby sustaining the angiogenic drive [170, 189].

Direct evidence for an endurance performance-enhancing effect of isolated increased vascular density is lacking, as all interventions (training, HIF-1α inhibitors, etc.) have effects beyond increasing vascular density. Nevertheless, a plethora of indirect evidence points to the importance of neovascularization for improving endurance performance. Both untrained and trained human individuals respond to endurance exercise training with increases in VEGF-A levels [170, 190] and, subsequently, in vascular density [167, 191]. In line with the importance of peripheral flow for endurance performance is the observation that the size and blood flow capacity of major vessels vary with the muscle groups experiencing the highest activity (e.g., the subclavian arteries in tennis players and the femoral arteries in cyclists) [192, 193]. Furthermore, polymorphisms in the *VEGFA* gene appear to be among the genetic determinants of human endurance performance, acting through *VEGFA* gene expression and maximal oxygen consumption [194, 195].

## The Practicalities of Angiogenic Doping

Because of the critical role of VEGF-A in the angiogenic response to endurance training, could VEGF-A be misused for angiogenic doping? Furthermore, could other interventions known to upregulate the *VEGFA* gene be exploited to achieve angiogenic doping indirectly?

### The Current State of Pro-Angiogenic Therapies

The same technologies developed for gene therapy in ischemic diseases could be utilized for doping, in the hope of improving athletic performance. Currently, the use of VEGFs and other pro-angiogenic growth factors for gene doping is generally considered theoretical only. The first clinical trials targeting ischemic hearts were conducted more than 30 years ago by the research group of Jeffrey Isner [196–199]. The understanding of what does and does not work for pro-angiogenic therapy has increased over the years [200, 201]. Although no FDA-approved drug or procedure exists yet, the technology has been refined through successive clinical trials to the point that many experts are cautiously optimistic that the first breakthroughs may occur soon [202–204]. The key features that characterize most ongoing phase II and III trials are the use of secreted angiogenic factors (VEGF-A, VEGF-D, FGF, HGF), local highly efficient viral or mRNA delivery, and preclinical large-animal models (pigs) to optimize dose, safety, and pharmacology [201]. The technological barriers to achieving clinically meaningful delivery—such as the need for multiple targeted injections of high-titer viral vectors via catheter—currently limit the application of this technology to therapeutic use in patients. However, as treatment methods evolve and become more accessible, there is a potential risk that such technologies could eventually be exploited for performance enhancement.

### Erythropoietin ahead of VEGFs in the mRNA Doping Landscape

Currently, commercially available gene doping products likely do not contain any VEGF-A or VEGF-D coding sequences, but have been shown to contain erythropoietin complementary DNA (cDNA). However, the amount of genetic material was deemed insufficient to produce an effect [205]. It is noteworthy that among the first commercially available off-the-shelf lipid nanoparticle (LNP)-encapsulated mRNA products were formulations designed to produce human erythropoietin upon injection [206, 207]. LNP-encapsulated mRNA is the technology that first debuted with the Moderna and BioNTech/Pfizer SARS-CoV-2 vaccines [208]. Depending on the injection site, different cell types would produce the erythropoietin (hepatocytes after intravenous injection, muscle cells after intramuscular injection, etc.). There are no published data on whether erythropoietin produced from synthetic mRNA in these cells can be distinguished from erythropoietin originating from mRNA transcribed from endogenous genes by its glycosylation patterns. In any case, the COVID-19 vaccines and mouse experiments with Epo-mRNA-containing LNPs demonstrate that this technology produces functional protein [209].

### Doping and Anti-Ischemic Therapy Do Not Have the Same Goals

Do the modest results and technological barriers mean that angiogenic gene therapy would not work for doping? Not necessarily, because the treatment goals and the target population for gene therapy for ischemic diseases are quite different from those for angiogenic doping. Highly trained young athletes presumably have a different response to angiogenic stimuli than the predominantly elderly population with coronary artery disease. While small improvements in capillarization and oxygen extraction efficiency (such as has been shown by Mortensen et al. in untrained males [210]) might not be clinically meaningful in a patient population, such differences may be significant for athletes, where differences are measured in tenths of a second.

### VEGF-A-Based Biological Drugs to Treat Ischemia

Neither the FDA nor the EMA have approved any VEGF-A therapy (protein, mRNA, or gene) for ischemic diseases. However, a gene therapy that delivers the *VEGFA* gene has been approved in Russia, under the name cambiogenplasmid (marketed as Neovasculgen^®^), for the treatment of peripheral arterial disease [211]. Its ‘naked plasmid’ technology is comparable to that used in phase II and III trials by the Isner group 30 years ago, none of which showed definitive clinical benefits, most likely due to very inefficient gene delivery [212].

Presumably similarly unsuitable for angiogenic doping purposes is telbermin (also known as sNN0029 in the context of amyotrophic lateral sclerosis treatment), an earlier attempt by Genentech to use VEGF-A protein as a pro-angiogenic drug to treat diabetic foot ulcers, which failed to meet the required efficacy in phase II. As a topical application, telbermin was designed to have a local effect only, relying on the wound to enter the body and unable to penetrate healthy skin [213, 214]. Previously, the same recombinant human VEGF-A had been used via intracoronary delivery in the VIVA trial for myocardial ischemia/coronary artery disease, with equally unimpressive results, likely due to poor uptake from the circulation, a short half-life, and the dose-limiting blood pressure-lowering effect of VEGF-A [215].

Merely changing to an intramyocardial delivery strategy did not improve the outcomes, with the REVASC (adenoviral VEGF-A_121_) and NORTHERN (intramyocardial plasmid VEGF-A_165_) trials similarly ineffective [216, 217]. Distinct from previous VEGF-A-based therapies is the currently still ongoing phase IIb trial to treat refractory angina with encoberminogene rezmadenovec (‘Adenovirus XC001’). This gene therapy produces three different VEGF-A isoforms (VEGF-A_121_, VEGF-A_165_, and VEGF-A_189_) from a single hybrid sequence, more closely mimicking the endogenous isoform distribution [218, 219].

### Gene Therapy and Gene Editing

Gene therapy is the delivery of external genetic information into cells, causing them to produce the protein of interest. Gene delivery can be achieved in different ways. Currently, viruses are the vectors of choice because they have evolved to efficiently transfer genetic material into cells [217], and many studies on angiogenic growth factor gene delivery have used viral vectors [200, 202, 204, 220–222]. However, alternative non-viral vectors do exist, such as liposomes, which have been used to deliver mRNA in the Comirnaty and Spikevax Covid-19 vaccines [223]. Vectors can be administered directly (e.g., by injection) into target tissues, such as skeletal or cardiac muscle, in a process referred to as in vivo gene therapy [224]. Alternatively, target cells may be genetically modified outside the body (ex vivo) under controlled culture conditions before reintroduction into the patient [225, 226]. In addition to gene delivery, endogenous genes can be selectively altered through gene editing techniques. Since its introduction in 2012, the clustered regularly interspaced short palindromic repeats (CRISPR)/CRISPR-associated protein (Cas) system has rapidly replaced previous gene editing technologies in preclinical and clinical studies [227]. Although there is only one FDA/EMA-approved drug on the market [226], the theoretical possibilities of gene editing for both therapeutic and doping purposes are enormous, as reflected by approximately 250 ongoing clinical trials [228]. One major implementation hurdle remains the effective and specific delivery of CRISPR agents into target cells. Ex vivo editing of blood cells and liver targeting are low-hanging fruits expected to cover most of the CRISPR drugs anticipated to receive market authorization over the next few years [229]. However, possibly superior technologies based on CRISPR-related systems are being discovered and developed [230].

### Which VEGF Would Work Best?

Among the angiogenic growth factors, VEGFs are considered the most specific, with VEGF-A and VEGF-D being the most promising targets for pro-angiogenic gene therapy [2]. While VEGF-A is the primary angiogenic factor during embryogenesis [42, 43], recent research suggests that other VEGFs may be superior during adulthood, particularly VEGF-D [79, 202, 204]. Specifically, the mature form of VEGF-D (also called ΔNΔC-VEGF-D) might be a superior option, because—unlike VEGF-A and VEGF-B [231]—it does not interact with VEGFR-1 and thus does not attract large amounts of inflammatory immune cells to the target tissue, and also is much more soluble since it does not—unlike the main isoform of VEGF-A—strongly interact with extracellular matrix and cell-surface associated proteoglycans [232]. Interestingly, active VEGF-D exists endogenously in two primary forms, one that is almost exclusively angiogenic [139] and another that supports both angiogenesis and lymphangiogenesis [78, 140], as reviewed by [55]. In addition, enhanced versions of VEGF-D with improved biological activity have been developed [233]. Lymphangiogenesis, which VEGF-C and VEGF-D can stimulate, appears beneficial in the experimental therapy of ischemic mouse hearts [234], and VEGF-C acts as a growth factor for the coronary vasculature [235]. Exercise increases the capillary filtrate, which needs to be drained, which is the primary task of the lymphatics [236, 237], but the effect of stimulating lymphangiogenesis on athletic performance is unknown. Apart from their angiogenic function, both VEGF-A and VEGF-C reportedly affect blood cell formation at the hematopoietic stem cell level [44, 238], and VEGF-A in the erythropoietic lineage [239]. Notably, based on mouse studies, VEGF-A has been proposed as a direct non-canonical, hypoxia-independent inducer of erythropoietin expression in perivascular stromal cells [240].

### Would Hypoxia-Inducible Factor-1α be Superior to VEGFs in Boosting Tissue Oxygenation?

The use of any single angiogenic factor might not be sufficient to establish a physiological vascular network. Because the transcription factor HIF-1 acts as a master regulator of a large number of hypoxia-responsive genes, including *VEGFA* (see Sect. 2.1), it might provide superior vascularization results compared with the angiogenic factors themselves [241–243], also due to the cell type-specific responses [115, 244]. A study by Elson et al. [245] demonstrated that the expression of a stable HIF-1α mutant transgene in mice led to the formation of an organized, functional blood vessel network. HIF-1’s suitability is further supported by the effects on the vascular system demonstrated by HIF-stabilizing drugs. HIF-stabilizing drugs target the same genes as HIF-1α itself, including *EPO* and *VEGFA*. PHD inhibitors, such as roxadustat, which is approved for the treatment of renal anemia in numerous countries [246], target not only erythropoietin but also upregulate VEGF-A levels in animal studies [247]. However, the effects of PHD inhibitors on angiogenesis and vascular parameters, such as capillary density and branching patterns, have not been reported in any of the clinical trials. Roxadustat doping was first seen in 2015, before the drug had received market authorization [248]. The emergence of structurally diverse roxadustat analogs poses challenges for standard detection by liquid chromatography-tandem mass spectrometry (LC–MS/MS) [249]. A method to detect all HIF stabilizers in a single assay, based on their common function, has been developed but may not yet be sensitive enough [250]. Beyond LC–MS-based methods, alternative rapid screening approaches are emerging. For example, a recently described surface-enhanced Raman spectroscopy (SERS) platform enabled sensitive detection of PHD inhibitors in aqueous media and saliva at sub-µg/L levels [251].

### Hypoxic Mimicry Targets Both Red Blood and Endothelial Cells

A chemically diverse range of chemical compounds (‘hypoxia mimetics’) can simulate low-oxygen conditions in the presence of normal oxygen levels and phenocopy the cellular and physiological responses to hypoxia. Such agents are widely used in research, as true hypoxia chambers for cell culture or animal husbandry are relatively expensive and cumbersome to use [252]. Because of the relative ease of access, hypoxia mimetics have already been experimented with for doping purposes as substitutes or enhancers of high-altitude training. In the following, we discuss carbon monoxide (CO), but it is only one of many compounds, including, among others, xenon and cobalt [253–255].

Unlike the unstable gasotransmitter nitric oxide (NO), which endothelial cells deploy as a signaling molecule in angiogenesis [256, 257], CO is stable and has been researched as a pharmacological agent [258–261]. Limited exposure to CO or CO-releasing molecules (CORMs) might be a means of increasing vascularization. In cultured vascular smooth muscle cells, which are a source of endogenous VEGF-A, a 1% CO atmosphere resulted in a very large 20-fold increase in VEGF-A generation [262]. While high concentrations of CO inhibit erythropoietin expression in cell culture [263], much lower concentrations can still inhibit prolyl hydroxylases and displace O_2_ from hemoglobin, resulting in hypoxia, which in turn upregulates erythropoietin and other hypoxia-responsive genes, such as *VEGFA*. Unsurprisingly, CO has emerged as a new doping agent [264]. As CO is highly lethal when overdosed [265], CORMs are considered safer than direct CO administration, and their action could be made more specific by coupling them to cell-specific antibodies to target their activity to particular organs or cell types, such as endothelial or smooth muscle cells. A safe CO delivery device (*Covox DS*) was developed by Ikaria, Inc. more than 20 years ago. Still, direct CO application was largely abandoned in favor of CORMs. In 2015, Ikaria was acquired by Mallinckrodt Pharmaceuticals, a company that later became infamous for its role in the US opioid crisis [266]. The use of CO is still somewhat common in exercise research as ‘CO rebreathing’ is an established research method for measuring the total hemoglobin mass in athletes [267]. CO—and, for that matter, all hypoxia mimetics when used systemically—not only target angiogenesis but also upregulate other hypoxia-responsive genes, most notably *EPO*. While most hypoxia mimetics have not been tested for effects on athletic performance, there is moderate evidence that some, including CO, may improve endurance performance [264].

### No Angiogenesis Response to Moderate Hypoxia without Exercise

Two or eight weeks of 4100-m altitude-induced increase in HIF-1α expression were not sufficient to increase VEGF-A mRNA expression or capillary density. However, the subjects in this study were not athletes, but rather regular people who continued their usual level of physical activity at altitude [268]. At an altitude of 4100 m, the oxygen pressure is reduced from ~ 21 kPa (sea level) to about 12.8 kPa [269]. While this is a 40% reduction, it is not comparable to the decrease in oxygen pressure at the muscle cell level during exercise, which can drop from ~ 4 kPa at rest [270, 271] to approximately 0.3–0.5 kPa during heavy exercise [270, 272], with mitochondrial PO_2_ potentially falling below 0.13 kPa (1 mmHg) under maximal exercise conditions [273].

The results of Lundby et al. [268] indicate that, on average, the cardiovascular reserve of most people is sufficient not to require any neovascular response to moderately high altitudes. They also agree with our shared experience of air travel: cabin pressure, typically corresponding to an altitude of 2400 m, does not appreciably cause breathlessness in most people, even though the oxygen pressure is reduced by more than 25%.

In rat studies, moderately low oxygen levels (12%, equivalent to ~ 4400 m of altitude) did not induce neovascularization in largely inactive muscles, but did so in active muscles. Interestingly, regional heterogeneity in angiogenesis was not only observed between different muscles but also within a single muscle [274, 275], discussed in detail by [276]. Likely, angiogenesis does not result solely from increased transcription of VEGF-A mRNA, but translational control mechanisms might also be at work [98, 277], which would not be reflected in the mRNA levels. After the angiogenic adjustment of capillary density, which occurs relatively early in endurance training [167], the VEGF-A concentrations required to maintain a vascular network are likely very similar, largely independent of its density.

### Pseudoanemia

The finding that some endurance athletes have relatively low hematocrit and hemoglobin levels is sometimes referred to as “sports anemia” or “pseudoanemia.” Despite relatively low hemoglobin and RBC concentrations, these athletes can have a large total hemoglobin mass due to their large total blood volume. While this phenomenon is not observed in all athletes, it is widespread because blood volume expansion is an early and consistent response to endurance training [278–281].

To what degree and by which mechanisms pseudoanemia contributes to improved performance has been controversial. Enhanced cardiac performance [282], reduced blood viscosity [283] and cardiovascular strain [284], as well as increased thermoregulatory capacity (more blood flow to the skin to cool down) [285, 286] have been proposed to contribute to the effect. Specific to runners is the controversial ‘footstrike hypothesis’, according to which older, less functional RBCs are mechanically destroyed during footstrike, thereby rejuvenating the RBC population [287–289]. Interestingly, the possible beneficial role of blood volume expansion as a compensatory mechanism has been re-examined, even in pathological contexts such as heart failure [290].

Beyond its relevance for masking erythropoietin doping, pseudoanemia could have significant implications for detecting angiogenic doping via the ABP. When new vessels form or existing vessels dilate without concurrent RBC stimulation, compensatory plasma volume expansion would decrease hemoglobin and RBC concentrations, creating a detectable hematological signature analogous to post-donation hemodilution.

### Risks and Problems Associated with the Execution of Angiogenic Doping

If pro-angiogenic responses are achieved through gene delivery or gene editing, all commonly known risks associated with such therapies become relevant [225, 291, 292]. While today’s regulatory requirements ensure relative safety and the highest possible quality in vector production [293], this is not the case when such tools are produced in rogue labs or administered outside a regulated healthcare system. Off-target effects can result from viral integration [8] or spurious annealing of the CRISPR guide RNA [292]. Repeated injections may induce immune reactions [225], and local overdosing may occur [294]. In addition to these generic side effects, specific side effects are associated with the overexpression of any particular gene. For angiogenic growth factors, concerns have included the vascularization of dormant tumors and other forms of aberrant angiogenesis (VEGF-A, VEGF-D) [83, 84, 154], heightened immune responses (VEGF-C) [295, 296], and inflammation (VEGF-A, VEGF-B) [231, 297]. Atherosclerosis, neovascular eye disorders, and many other diseases have been associated with the overexpression of VEGFs [156, 298]. It is unknown currently whether there would be any specific risks associated with a doping-induced denser vascular network, similar to the particular risk of erythropoietin-induced hyperviscosity syndrome (‘blood sludging’) leading to blood clots and possibly even sudden cardiac death.

## Detection of Angiogenic Doping

### Detecting Angiogenic Gene Doping

**Fig. 5:**
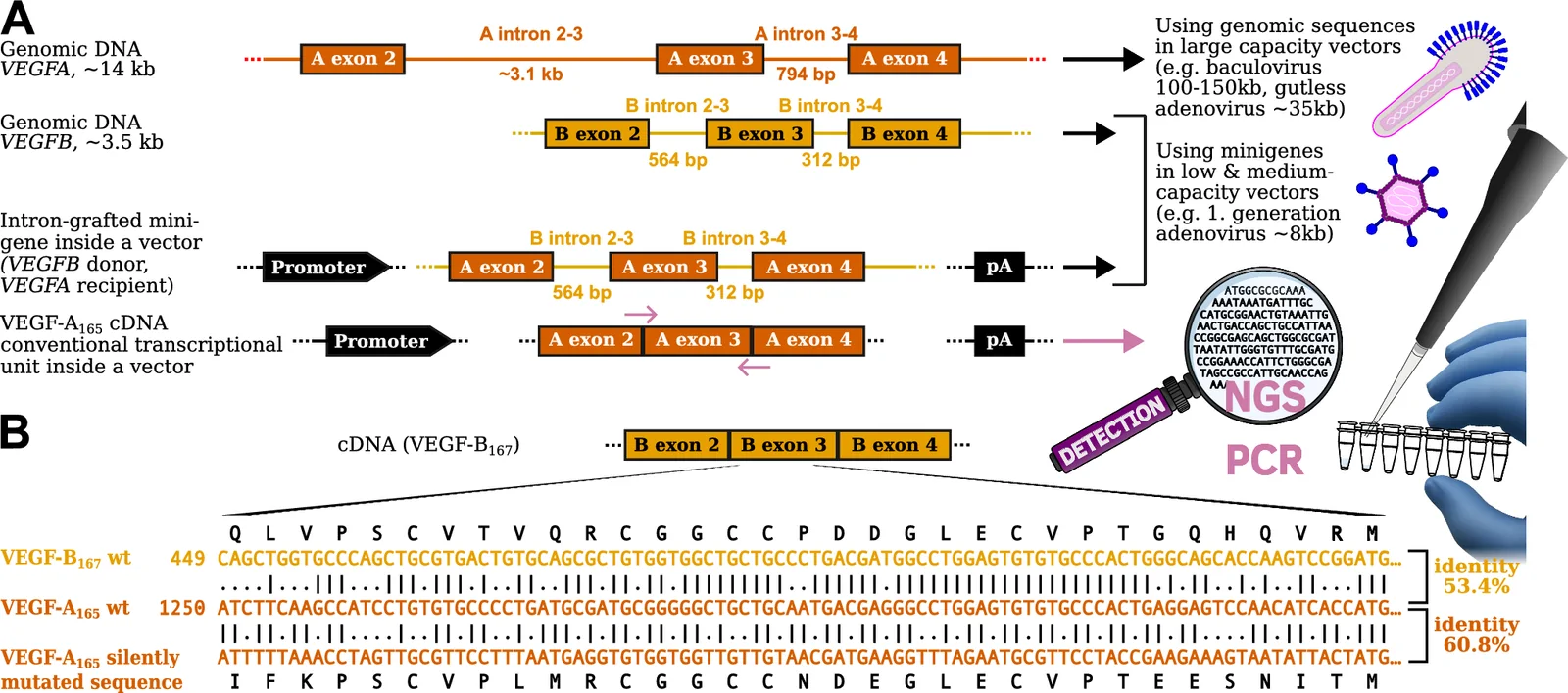
Detection of angiogenic gene doping. **A** Even the most advanced and sensitive detection methods rely on some form of discriminatory PCR, e.g., using exon junction-spanning primers to detect cDNA or next-generation sequencing to detect missing exon–intron junctions or novel junctions [205, 302]. However, large-capacity vectors can accommodate full genes, while low- and medium-capacity vectors can use intron grafting to evade detection. **B** Unlike the unique *EPO* gene, there are five *VEGF* genes with significant homology. Due to the degeneracy of the genetic code, silent mutagenesis can create thousands of different nucleotide sequences for *VEGF* genes while maintaining their amino acid sequence. In the example shown, the VEGF-A_165_ cDNA sequence was maximally modified by silent mutagenesis to become almost as different from its wild-type sequence as it is from the paralogous VEGF-B_167_ cDNA sequence. Due to their close evolutionary relationship [306], this is possible for all VEGFs. DNA swarms, all coding for the same protein, can be used to increase the detection threshold. The numbering of the nucleotides is according to NM_001171626 (VEGF-A) and NM_001243733.2 (VEGF-B). Exons are not drawn to scale. The % identities refer to the cDNAs of the entire mature proteins, not only to the exon 3 sequence shown. *A exon/intron* VEGF-A exon/intron, *B exon/intron* VEGF-B exon/intron, *bp* base pairs, *cDNA* complementary DNA, *NGS* next-generation sequencing, *pA* polyadenylation sequence, *PCR* polymerase chain reaction, *wt* wild type

Shortly after it was banned in sports, WADA began funding research into detecting gene doping [299]. Yet, two decades later, there is no standardized detection method [300]. The tests that could be used to screen for gene doping include detection of the doping agent itself, specific biomarkers in blood samples, and atypical DNA signatures by real-time PCR and next-generation sequencing (NGS) [225, 301]. While methods have been proposed to detect atypical DNA signatures [205, 301–305], multiple methods exist to bypass them. For example, detecting exon–exon junctions, which are absent in genomic DNA, can be bypassed using intron grafting (see Fig. 5).

Notably, the human genomic VEGF-B sequence—including six introns—measures only 3.5 kb, from start-ATG to stop-TGA [307], and can fit into cargo-limited viral vectors. Cargo up to ~ 38 kb has been inserted into baculoviruses [308], long enough to accommodate most human genes, which have a mean length of ~ 28 kb [309] and a median of ~ 24 kb [310]. An upper limit on the insert length for baculovirus genomes has not been established, as the capsid size appears to adapt to genome size, potentially allowing up to 100-kb inserts [311], thereby eliminating the need for intron grafting for all but the largest genes. Silent mutagenesis is another effective countermeasure to PCR-based detection methods. However, it remains to be shown how maximized codon optimization would perform against hybridization-based methods such as those proposed by de Boer et al. [301].

### Detecting Angiogenic Protein Doping

Crucially, in gene doping, the active pharmaceutical protein is produced by the athlete’s cells and is therefore potentially indistinguishable from the ‘normal’ protein if expressed orthotopically. Not only is the molecular identity of angiogenic proteins in question, their levels also might not reliably distinguish between endogenous and doping-induced angiogenic factors, because the levels of angiogenic proteins in the blood plasma of normal healthy human individuals vary substantially. For example, VEGF-A levels in blood plasma can range from undetectable to nearly 500 pg/mL [312, 313], making the use of cutoff values challenging. On the upside, despite significant interindividual variability, plasma and serum VEGF-A levels in any given individual seem to be relatively stable, at least up to 6 months, and show no circadian variation [314, 315].

**Fig. 6:**
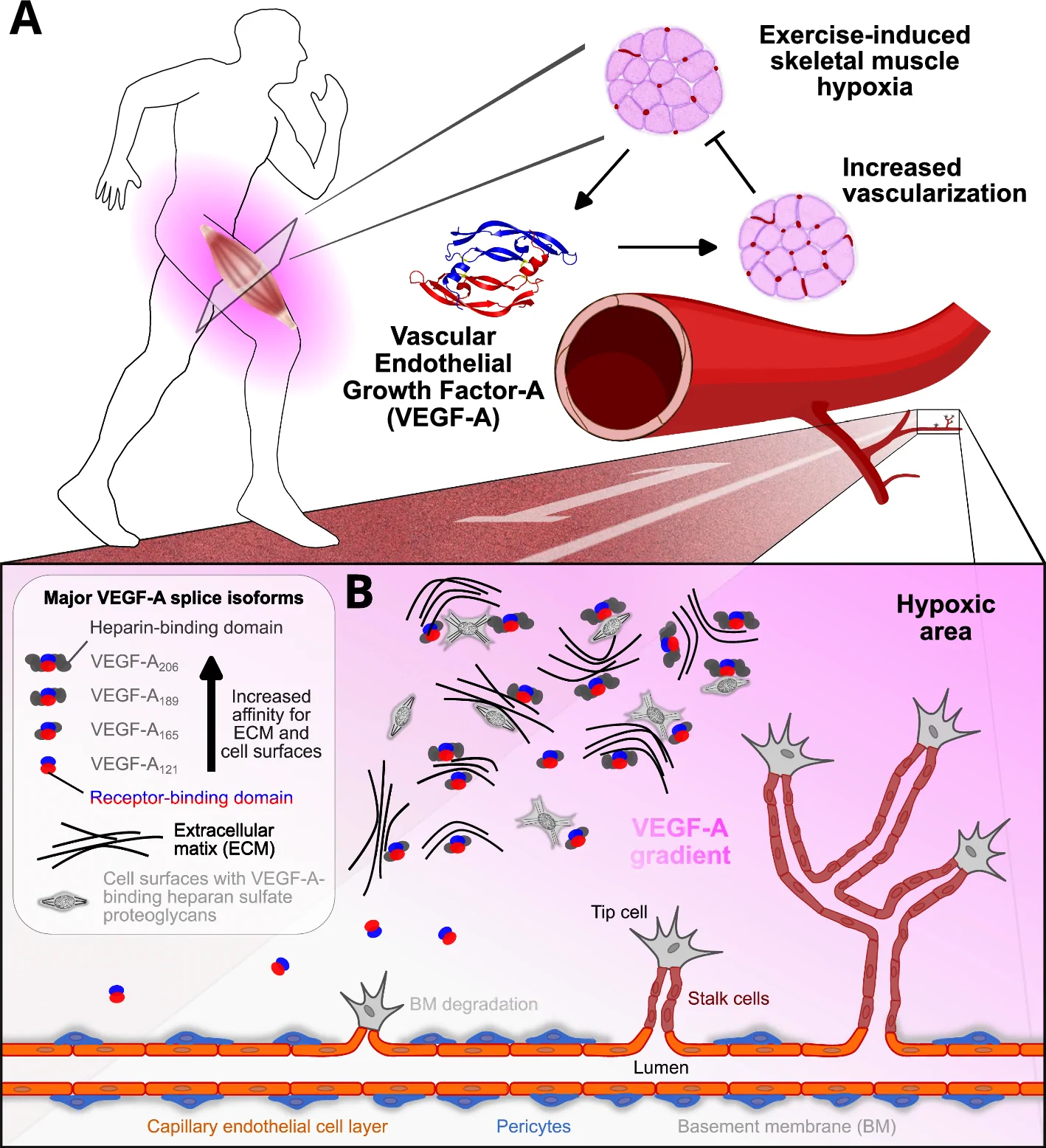
Current model of VEGF-A action via hypoxia-generated growth factor gradients and negative feedback. **A** Cells that experience hypoxia (due to tissue growth, decreased oxygen delivery, or increased consumption) upregulate hypoxia-regulated genes in a cell-type–specific manner; for example, muscle cells begin to produce VEGF-A [171], and renal cells erythropoietin [317]. Many cell types can respond to hypoxia by increasing VEGF-A expression, which attempts to restore normoxia via increased vascularization. **B** It is generally thought that by their differential affinity to extracellular matrix and cell surface heparan sulfate proteoglycans, the various VEGF-A isoforms lay down a growth factor gradient, which is sensed by specialized endothelial cells at the growth front (tip cells), leading the angiogenic sprout to form a hierarchical vascular network to supply the hypoxic tissue with oxygen. Interestingly, there is surprisingly little direct evidence for VEGF-A gradients in vivo [318, 319], suggesting that other mechanisms may be involved

The detection of the angiogenic protein itself presumes that the protein (a) is a secreted protein and (b) enters the circulation or urine. Neither can be assumed. The transcription factor HIF-1α (see Fig. 2) is highly angiogenic but not secreted from cells [242]. Furthermore, VEGF-A is a tissue hormone, that is, unlike erythropoietin, not systemically distributed by the blood. Instead, VEGF-A is secreted into the extracellular space and encounters the VEGF receptors on endothelial cells via the interstitium from the abluminal side [187, 316]. While the soluble VEGF-A_121_ isoform might leak into the circulation to some extent, the longer heparin-binding isoforms of VEGF-A are much less likely to show up in the blood as they are bound tightly to the extracellular matrix and cell surfaces (see Fig. 6).

### Indirect Detection Methods

Indirect methods attempt to detect the means or consequences of doping. For example, tests could screen for the gene-doping delivery vector or recent blood vessel growth. Viruses used to deliver the genetic cargo would result in a measurable immune response [225]. Also, other delivery systems, such as lipoprotein nanoparticles or engineered extracellular vesicles, would leave traces for at least a short time in the athlete’s body. Because the ABP has proven to be a valuable tool [320, 321], its use could be expanded to encompass additional indirect biomarkers that may indicate angiogenic doping. It is unclear what biomarkers these would be, especially since tissue biopsies are currently the only reliable method for quantifying parameters indicative of pro-angiogenic interventions at the capillary level, such as vascular and branching density, vessel diameter and length, or vascular volume. Currently, there is no non-invasive imaging technology that can quantitatively image the microvasculature at the required resolution (< 1 µm) and depth for large leg muscles [322]. However, microRNA in the blood might serve as a proxy, at least for HIF-upregulating agents [255, 323], as hypoxia-mediated regulation of angiogenesis involves several specific microRNAs [324].

Unlike in the pre-ABP era, with fixed cut-off values (e.g., hemoglobin 150 and 175 g/L for women and men, respectively), the ABP uses, among many other parameters, previous test results to predict individualized upper and lower limits for blood parameters [325]. While retrospective studies suggest that athletes have ‘taken the fast lane’ in the past [320], slow changes that do not result in an abnormal blood profile score may represent a potential ‘Achilles heel’ of the ABP. Doping, increased slowly over several years, could produce physiological changes that fall within normal biological variation or phenocopy improvements gained through a sustained training effort [325].

### Gene Doping Versus Gene Editing

In the long run, the single most critical technology to monitor is CRISPR, as it can perform precise genetic modifications to existing genetic material in a live organism. While FDA-approved treatments are currently limited to a single ex vivo drug [226], there is little doubt that CRISPR and similar gene-editing technologies will be the future of biologicals [227]. If the delivered gene-editing agents are transient enough and the editing is limited to single base-pair mutations, the results could only be distinguished from hereditary mutations by genome sequencing. Even with great technological advances, it is unlikely that we will achieve editing efficiency in vivo of close to 100% in the near future. But since genetic mosaics do also occur naturally, the mere existence of a mixed genotype would not constitute proof of editing. A detailed genomic analysis, including single-cell sequencing, might be required. However, single-cell sequencing is not only expensive but also requires an invasive biopsy. Since CRISPR technology can also arbitrarily change gene expression without modifying DNA by targeting methylating enzymes or transcription factors, detecting the CRISPR delivery system—despite its transient nature—may be easier than detecting the CRISPR effect.

Science has already identified a few naturally occurring mutations that can increase athletic performance [326]. Feasibility studies of such modifications have been performed in mice [327]. Once an athlete is found to carry a known performance-enhancing mutation, what would be the consequences? If it was acceptable when the mutation originated from ‘natural’ inheritance or from a spontaneous mutation during embryonic development, why would an ‘artificially’ acquired mutation be treated differently? Assuming sufficiently advanced technology, there appears to be no way to determine whether a person is mutated by nature or genetic engineering. Even genotyping all alive and deceased athlete's relatives would leave doubt as humans naturally acquire surprisingly many mutations during embryonic development [328].

### Plasma VEGF-A: An Unreliable Epiphenomenon

There are several methods for measuring VEGF levels in biological samples, such as bioassays and enzyme-linked immunosorbent assays (ELISAs). Bioassays use growth factor–sensitive cell lines, whereas ELISAs are based on VEGF-A-specific antibodies. ELISA tests have been the method of choice in most preclinical studies [329–331]. The best current ELISAs detect VEGF-A levels down to the single-digit pg/mL range [312], and typical VEGF-A concentrations in blood plasma are in the double-digit pg/mL range, below what is considered necessary for a biological effect.

Currently, none of these tests has FDA clearance, and thus they are not widely available. However, unlike erythropoietin, whose producer cells are in close contact with permeable capillary networks [332], hypoxia-induced VEGF-A is, by definition, produced far distant from capillaries, and there is no reason to believe that it serves—the platelet VEGF-A pool excluded [333]—any purpose in the systemic blood; its presence in plasma is likely an epiphenomenon.

Since VEGF-A can be produced by most cell types in the human body that are exposed to hypoxia, the glycosylation pattern of endogenous human VEGF can be heterogeneous [334]. Detecting recombinant CHO- or HEK-293–produced VEGFs might therefore be less straightforward than detecting recombinant erythropoietin, whose production is almost exclusively limited to a single cell type in the kidneys [335], and any size aberration of erythropoietin is likely to represent exogenous protein. Unlike erythropoietin, VEGF-A also exists in a variety of different isoforms. Only the smallest isoforms (VEGF-A_110_, VEGF-A_121_, VEGF-A_145_, VEGF-A_165_) are thought to be soluble and to leak into the vasculature, because the longer VEGF-A isoforms are strongly ‘heparin-binding’, meaning they bind to cell surfaces and extracellular matrix due to their interaction with heparan sulfate proteoglycans (HSPGs) [55]. However, these longer VEGF-A forms are essential for angiogenesis [336, 337]. The major isoform in humans (VEGF-A_165_) is partly soluble and partly HSPG-bound, but in some mammals, isoforms corresponding to the human VEGF-A_165_ do not exist, and the major isoform (VEGF-A_188/189_) is strongly HSPG-binding [306], indicating that VEGF-A_189_ might also work well in humans (see Fig. 6).

Once produced by hypoxic cells, VEGF-A distributes in the interstitial space, and its interactions with VEGF receptor-1 or -2 are thought to happen on the abluminal side of the endothelial cell [338, 339]. Thus, the VEGF-A measured in blood plasma represents leakage, transendothelial transport, or is introduced into the blood via lymphatic drainage. This view is supported by the typically low VEGF-A plasma concentrations, which are often a fraction of what is needed to elicit an angiogenic response. In line with this view is the fact that the correlation of plasma or serum VEGF-A with angiogenesis is not very strong. While an increase in interstitial VEGF-A will also increase plasma VEGF-A, individual differences in permeability (how much VEGF-A leaks into the blood) and lymphatic function (how much VEGF-A is carried into the blood via interstitial fluid drainage) easily explain the variation between individuals. In cancer patients, plasma VEGF-A levels are significantly higher, most likely due to VEGF-A entering the blood via the tumor’s leaky vessels [340]. High VEGF-A plasma levels have consistently been associated with disease severity (as a prognostic marker), but their use as a biomarker to predict the response to antiangiogenic cancer treatment remains elusive [341, 342], and may even depend on the sampling method [330].

The distribution of receptors on the luminal and abluminal sides of the cell has been modelled. While the model was relatively simple (i.e., it included only two VEGF-A isoforms, two receptors, and one co-receptor), an increase in VEGF-A production in this model, first and foremost, increased the internalization of VEGFR-1, which primarily functions as a non-angiogenic decoy receptor [316]. Not much wetlab research has compared luminal and abluminal signaling; however, based on the available literature, it is very likely that the spatial distribution of signaling is a crucial factor in the response to VEGF-A [187, 188, 343].

## Can Angiogenic Doping Boost Athletic Performance?

Can angiogenic doping increase athletic performance? While delivering VEGF-A results in an angiogenic response, it has been shown not to increase blood supply in healthy tissues that already have an adequate blood supply. VEGF-A gene therapy effects are more pronounced in tissues with impaired blood flow, such as ischemic tissues [200, 216]. Studies have shown that overexpressing or overdelivering VEGF-A leads to the formation of a chaotic, leaky, and irregular blood vessel network that is incapable of upgrading the vascular system [153]. It is not entirely clear how a sophisticated vascular patterning is achieved, which optimizes blood oxygen uptake and delivery, but growth factor gradient formation, exploiting the differential affinities of the multiple VEGF-A isoforms, is thought to play a central role [318, 319] (see Fig. 6).

The treatment of ischemic diseases with VEGF-A has shown that many hurdles have to be overcome to achieve clinically relevant results [200]. However, in highly ischemic tissues of coronary artery disease patients, impaired blood flow is better than none. It has also been speculated that VEGF-A alone may not be sufficient to improve blood flow, and that a combination of angiogenic growth factors in appropriate ratios might be necessary [344]. For example, combining VEGF-A with angiopoietin-1 could reduce the leakiness of newly formed vessels, leading to more physiological outcomes [344, 345].

### Systemic Versus Local Delivery and the Importance of Gradients

The current view is that effective direct VEGF application requires complex and tightly controlled delivery methods, making it largely impractical for doping attempts currently. Systemic administration of larger amounts is considered hazardous due to the vasodilatory effect of VEGF-A observed in early clinical trials and animal studies [215, 346]. Systemic administration of VEGF-A might not have any positive impact on angiogenesis. In one study, intravenous delivery of a VEGF-A form engineered for prolonged half-life actually resulted in a reduction in capillary density in the kidney target organ [347].

Hence, as a protein, VEGF-A can presently only be applied locally. However, the distribution of locally applied VEGF-A does not replicate the endogenous growth factor gradients necessary to organize angiogenic sprouts and ultimately result in a hierarchical, functional network. In the absence of these instructive gradients, VEGF-A application typically results in nondirectional growth, which does not necessarily improve oxygen and nutrient supply (see Fig. 4B). For example, topically applied VEGF-A in the chorioallantoic membrane assay results in extensive angiogenesis, but not in a hierarchical, streamlined network [24, 25]. The early clinical trials to increase vascularization of ischemic hearts using VEGF-A had no clinically meaningful effects, at least partly for the same reasons, despite measurable angiogenesis [200].

### Angiogenic Doping—without Genes and without Increasing VEGF Levels

The goal of angiogenic doping is to increase vascular density slightly and uniformly across large areas of the body, particularly in skeletal muscle. This goal has been achieved, but with lymphatic vessels rather than blood vessels. Kataru et al. increased lymphatic vascular density in mice throughout the body without altering VEGF-C levels [348]. This increase was achieved by conditionally inactivating a gene that negatively regulates VEGF-C-mediated intracellular signaling in lymphatic endothelial cells. The same paradigm should work equally well in blood vascular endothelial cells with VEGF-A signaling. Kataru et al. used genetic engineering to implement ‘molecular nudging’ of the *PTEN* gene, but pharmacological inhibitors of *PTEN* or other phosphatases could enable targeting of the intracellular signaling of VEGFR-2 [349]. Nobody has been searching intensively for suitable compounds, as the primary goal of the pharmaceutical industry was the opposite: to find inhibitors of intracellular signaling downstream of the VEGF receptors, such as receptor tyrosine kinase (RTK) inhibitors [350]. Protein tyrosine phosphatase (PTP) inhibitors, on the other hand, target the off switches for RTK signaling. VE-PTP and PTP1b are examples of endothelial cell-specific PTPs [351, 352]. The VE-PTP inhibitor razuprotafib has been tested in clinical trials, but nothing is known about its effects on sports performance [353, 354].

Small-molecule drugs such as PTP and PHD inhibitors could also be targeted to the vasculature. Targeting pericytes or vascular smooth muscle cells, which are natural sources of VEGF-A, could be done with an antibody conjugate directed against platelet-derived growth factor receptor-β or other mural or endothelial cell surface markers. Targeting would allow for a sufficient local concentration despite almost undetectable systemic levels.

### Current Technical Barriers and Suspected Current Adoption

Based on the technical limitations of delivery described in Sects. 4.1 through 4.5, it appears unlikely that currently available pro-angiogenic gene therapies, CRISPR or mRNA-based drugs, will be misused for doping purposes in the near future. However, the same cannot be said for erythropoietin delivery, as microdosing of mRNA drugs appears fully feasible with currently available agents.

The leading VEGF candidate for future misuse would likely be VEGF-D, not only because of its superior biological properties, but also because many fewer methods exist for its detection, as it is the most recently discovered and arguably least researched VEGF. Very different from VEGFs are hypoxia mimetics and phosphatase inhibitors, which are currently available and easy to administer. Microdosing of hypoxia mimetics, including CO, could produce a small but meaningful effect on erythropoiesis, which might go undetected due to the concurrent effect on angiogenesis and blood volume expansion. From a detection point of view, CO is very similar to high-altitude training and indistinguishable from environmental exposure [355]. Similarly, systemically microdosed phosphatase inhibitors could slightly increase the response to normal levels of VEGF-A. Moreover, the development of small-molecule analogs is well understood [356], and current detection efforts do not even cover all known compounds.

While all such pharmacological interventions carry significant risks, moderate high-altitude hypoxia, in contrast, appears to be cardioprotective in both animal models and humans [357, 358]. However, the mechanisms underlying protection remain unclear because the multitude of concurrent physiological changes associated with high-altitude exposure creates uncertainty about which changes are causative [359]. Additionally, similar benefits may be achieved through different adaptive strategies, as genetic variation shows [360]. In the absence of high-altitude hypoxia, aerobic exercise is the most effective way to achieve a similar effect [361]. Table 2 summarizes the existing and potential methods to stimulate angiogenesis for doping purposes, along with their detectability and practical considerations.

**Table 2:** Existing and potential methods to stimulate angiogenesis

Agent	Example(s)	How to detect it	(Likelihood of) current use	Remarks: ease of synthesis, acquisition, and application
(Simulated or real) altitude living/training	Hypoxic tents, hypobaric chambers	No need for detection, as it is not banned	Common	Easy
Iron competitors and chelators	Carbon monoxide, CORMs, deferoxamine, Co^2+^	Spectrophotometry based on the different absorption maxima of carboxyhemoglobin vs other hemoglobin forms; smokers always test positive The iron chelator deferoxamine can be detected by mass spectrometry or HPLC, but has a very short half-life [253]	High	Readily available, correct dosing is challenging for carbon monoxide
PHD/HIF inhibitors	Roxadustat (FDA-approved), IOX5 (ongoing clinical studies)	Different types of mass spectrometry	Medium (new compounds are being developed)	Readily available, oral administration
PTP inhibitors	Razuprotafib (AKB-9778)	Different types of mass spectrometry	Medium	Compounds are commercially available. Failed in clinical trials to treat, e.g., diabetic nephropathy and proliferative diabetic retinopathy, but might still work for doping
Targeted small-molecule drugs	Antibody conjugates of HIF stabilizers and hypoxia mimetics	Difficult to detect due to small amounts and targeting	Medium–low	Requires custom synthesis and injection
Gene therapy	AdVEGF-D, AdHIF-1α	PCR (within a few weeks of use)	Low	Requires expensive and specialized equipment for production and administration
Growth factor proteins	VEGF-A, VEGF-D	Western blot (within a few weeks of use)	Low	Currently, it remains challenging to deliver the agent in a way that benefits athletic performance
mRNA	VEGF-A, VEGF-D, HIF-1α	RT-PCR (within a few weeks of use)	Low	Currently, it is technically challenging to deliver the agent in a way that benefits athletic performance
Gene editing, e.g., CRISPR-based drugs	SNP-editing of *EPO* or *VEGFA* promoters to slightly increase the endogenous hormone levels	Depending on the delivery system, (RT-) PCR could be used within a few weeks of application. Mosaicism resulting from incomplete editing is likely to remain undetectable for years to come	Low, but rapidly increasing	Delivery of the CRISPR drugs is still a high technical hurdle, but it might be easier to achieve for doping than for therapy. Target cells can be muscle cells, muscle-derived stem cells, or satellite cells
miRNA	miR-126, miR-210	Dependent on the delivery system. Targeted delivery (e.g., aptamers or customized extracellular vesicles) can be challenging to detect	Very low	Requires expensive and specialized equipment for production and administration
Slow-release biomaterials	Hyaluronic acid hydrogels, poly- (lactic-co-glycolic acid), various nanoparticles	Slow-release materials are relatively easy to detect because of their long biological half-lives	Very low	It can be used in combination with any of the above methods

The table is not exhaustive; only a few examples are given for each agent class

*Ad* adenoviral, *CORM* carbon monoxide-releasing molecule, *CRISPR* clustered regularly interspaced short palindromic repeats, *HPLC* high-performance liquid chromatography, *HIF* hypoxia-inducible factor, *miRNA* microRNA, *PHD* prolyl hydroxylase domain, *PTP* protein tyrosine phosphatase, *(RT-) PCR* (reverse-transcription) polymerase chain reaction, *SNP* single-nucleotide polymorphism

## Conclusions

While the ABP has significantly curbed traditional blood doping and erythropoietin misuse, the evolving landscape of performance enhancement now faces the formidable challenge of *next-generation doping*. Angiogenic doping might be an attractive use case for athletes aiming to implement *next-generation doping*, particularly through the manipulation of VEGF signaling via HIF-1α, prolyl hydroxylases, or protein tyrosine phosphatases. Perhaps we are failing already to detect some of the cutting-edge angiogenic doping techniques. For example, PTP inhibitors are not routinely checked by WADA, and targeted small-molecule drugs could easily fall below the detection threshold. If this is the case, the true prevalence of doping among top athletes might be much higher than typically assumed [362].

Enhancing oxygen delivery, a critical determinant of endurance performance, can be achieved not only by altering blood composition but also by increasing vascularization through angiogenesis. The observed interplay among hypoxia-inducible factors, prolyl hydroxylases, and emerging agents such as targeted hypoxia mimetics suggests ample potential for illicit manipulation. In contrast, achieving functional and beneficial angiogenesis by direct application of angiogenic factors such as VEGF-A or VEGF-D is complex, requiring precise control of growth factor gradients and isoform-specific effects, which are not easily achievable in the context of doping at this time.

The pursuit of angiogenic doping carries significant and unpredictable health risks, including the potential for triggering tumor progression and the exacerbation of various non-malignant diseases. In the long run, the detection of angiogenic gene doping or editing poses formidable challenges, given the possible orthotopic expression of endogenous proteins, the limitations of current non-invasive imaging technologies for the skeletal muscle microvasculature, and the improvements of advanced gene-editing techniques such as CRISPR.

If history is any guide, athletes will experiment with pro-angiogenic drugs and regimens in the hope of gaining a performance advantage—even before the FDA approves such agents. Some of these substances are already accessible, while others, such as gene doping and gene editing, still face significant technical challenges before becoming practical for doping purposes. Angiogenic doping, along with its potential long-term physiological consequences and the difficulties of detection, warrants dedicated research to safeguard both the integrity of competitive sport and the health of athletes.

## Supplementary Information

Below is the link to the electronic supplementary material.

Supplementary file1 (PDF 121 KB)

## References

[1] Krumm B, Botrè F, Saugy JJ, Faiss R. Future opportunities for the Athlete Biological Passport. Front Sports Act Living. 2022;4:986875. 10.3389/fspor.2022.986875. 36406774 10.3389/fspor.2022.986875 PMC9666424

[2] Pokrywka A, Kaliszewski P, Majorczyk E, Zembroń-Łacny A. Genes in sport and doping. Biol Sport. 2013;30:155–61. 10.5604/20831862.1059606. 24744482 10.5604/20831862.1059606 PMC3944571

[3] Gould D. Gene doping: gene delivery for Olympic victory. Br J Clin Pharmacol. 2013;76:292–8. 10.1111/bcp.12010. 23082866 10.1111/bcp.12010 PMC3731603

[4] Wilkin T, Baoutina A, Hamilton N. Equine performance genes and the future of doping in horseracing. Drug Test Anal. 2017;9:1456–71. 10.1002/dta.2198. 28349656 10.1002/dta.2198

[5] Puchalska M, Witkowska-Piłaszewicz O. Gene doping in horse racing and equine sports: current landscape and future perspectives. Equine Vet J. 2025;57:312–24. 10.1111/evj.14418. 39267222 10.1111/evj.14418 PMC11807943

[6] IOC, WADA. Olympic Movement Anti-Doping Code 1999, Appendix A. IOC, WADA; 2003. https://web.archive.org/web/20031011110933/http://www.wada-ama.org/docs/web/research_science/prohibited_substances/prohibited%20classes%20of%20substances%20and%20prohibited%20methods%202003.pdf

[7] Philostratus LF. De Gymnastica. 200 AD. https://www.perseus.tufts.edu/hopper/text?doc=Perseus:text:2008.01.0600

[8] Unal M, Ozer Unal D. Gene doping in sports. Sports Med. 2004;34:357–62. 10.2165/00007256-200434060-00002. 15157120 10.2165/00007256-200434060-00002

[9] Hoyt A. Blood Doping Goes Back to the Future. The Atlantic. 2010. https://www.theatlantic.com/technology/archive/2010/11/blood-doping-goes-back-to-the-future/65508/. Accessed 30 Jun 2025

[10] Chapman RF. The individual response to training and competition at altitude. Br J Sports Med. 2013;47:i40–4. 10.1136/bjsports-2013-092837. 24282206 10.1136/bjsports-2013-092837 PMC3903142

[11] Saltin B, Calbet JAL. Point: in health and in a normoxic environment, V̇o2 max is limited primarily by cardiac output and locomotor muscle blood flow. J Appl Physiol. 2006;100:744–8. 10.1152/japplphysiol.01395.2005. 16421282 10.1152/japplphysiol.01395.2005

[12] Warburton DER, Gledhill N. Comment on point:counterpoint “in health and in a normoxic environment, V̇o2 max is/is not limited primarily by cardiac output and locomotor muscle blood flow.” J Appl Physiol. 2006;100:1415–6. 10.1152/japplphysiol.01597.2005. 16540715 10.1152/japplphysiol.01597.2005

[13] Wikipedia. List of doping cases in cycling. Wikipedia. 2025. https://en.wikipedia.org/wiki/List_of_doping_cases_in_cycling. Accessed 4 Jul 2025

[14] Sottas P-E, Robinson N, Rabin O, Saugy M. The Athlete Biological Passport. Clin Chem. 2011;57:969–76. 10.1373/clinchem.2011.162271. 21596947 10.1373/clinchem.2011.162271

[15] Schumacher YO, Saugy M, Pottgiesser T, Robinson N. Detection of EPO doping and blood doping: the haematological module of the Athlete Biological Passport. Drug Test Anal. 2012;4:846–53. 10.1002/dta.406. 22374784 10.1002/dta.406

[16] Wakeham DJ, Hearon CM, Levine BD. The effect of chronic habitual exercise on oxygen carrying capacity and blood compartment volumes in older adults. J Appl Physiol. 2024;136:984–93. 10.1152/japplphysiol.00706.2023. 38420680 10.1152/japplphysiol.00706.2023 PMC11305637

[17] Otto JM, Montgomery HE, Richards T. Haemoglobin concentration and mass as determinants of exercise performance and of surgical outcome. Extreme Physiol Med. 2013;2:33. 10.1186/2046-7648-2-33. 10.1186/2046-7648-2-33 PMC3874847 24280034

[18] Schmidt W, Prommer N. Impact of alterations in total hemoglobin mass on V˙O2max. Exerc Sport Sci Rev. 2010;38:68. 10.1097/JES.0b013e3181d4957a. 20335738 10.1097/JES.0b013e3181d4957a

[19] Olfert IM, Howlett RA, Tang K, Dalton ND, Gu Y, Peterson KL, et al. Muscle-specific VEGF deficiency greatly reduces exercise endurance in mice. J Physiol. 2009;587:1755–67. 10.1113/jphysiol.2008.164384. 19237429 10.1113/jphysiol.2008.164384 PMC2683962

[20] Chong ZZ, Kang J-Q, Maiese K. Angiogenesis and plasticity: role of erythropoietin in vascular systems. J Hematother Stem Cell Res. 2002;11:863–71. 10.1089/152581602321080529. 12590701 10.1089/152581602321080529

[21] Malm CB, Khoo NS, Granlund I, Lindstedt E, Hult A. Autologous doping with cryopreserved red blood cells – effects on physical performance and detection by multivariate statistics. PLoS ONE. 2016;11:e0156157. 10.1371/journal.pone.0156157. 27284981 10.1371/journal.pone.0156157 PMC4902314

[22] Solheim SA, Bejder J, Breenfeldt Andersen A, Mørkeberg J, Nordsborg NB. Autologous blood transfusion enhances exercise performance—strength of the evidence and physiological mechanisms. Sports Med - Open. 2019;5:30. 10.1186/s40798-019-0204-1. 31286284 10.1186/s40798-019-0204-1 PMC6614299

[23] Nielsen JL, Frandsen U, Jensen KY, Prokhorova TA, Dalgaard LB, Bech RD, et al. Skeletal muscle microvascular changes in response to short-term blood flow restricted training—exercise-induced adaptations and signs of perivascular stress. Front Physiol. 2020;11:556. 10.3389/fphys.2020.00556. 32595516 10.3389/fphys.2020.00556 PMC7303802

[24] Wilting J, Christ B, Bokeloh M, Weich HA. In vivo effects of vascular endothelial growth factor on the chicken chorioallantoic membrane. Cell Tissue Res. 1993;274:163–72. 7694800 10.1007/BF00327997

[25] Oh S-J, Jeltsch MM, Birkenhäger R, McCarthy JEG, Weich HA, Christ B, et al. VEGF and VEGF-C: specific induction of angiogenesis and lymphangiogenesis in the differentiated avian chorioallantoic membrane. Dev Biol. 1997;188:96–109. 10.1006/dbio.1997.8639. 9245515 10.1006/dbio.1997.8639

[26] Tona K, Voemesse K, N’nanlé O, Oke OE, Kouame YAE, Bilalissi A, et al. Chicken incubation conditions: role in embryo development, physiology and adaptation to the post-hatch environment. Front Physiol. 2022;13:895854. 10.3389/fphys.2022.895854. 35677093 10.3389/fphys.2022.895854 PMC9170334

[27] Wagner PD. Determinants of maximal oxygen transport and utilization. Annu Rev Physiol. 1996;58:21–50. 10.1146/annurev.ph.58.030196.000321. 8815793 10.1146/annurev.ph.58.030196.000321

[28] Bassett DR Jr, Howley ET. Limiting factors for maximum oxygen uptake and determinants of endurance performance. Med Sci Sports Exerc. 2000;32:70–84. 10.1097/00005768-200001000-00012. 10647532 10.1097/00005768-200001000-00012

[29] Saugy M, Leuenberger N. Antidoping: from health tests to the athlete biological passport. Drug Test Anal. 2020;12:621–8. 10.1002/dta.2773. 31994337 10.1002/dta.2773

[30] World Anti-Doping Agency. WADA Prohibited List 2025. World Anti-Doping Agency; 2024. https://www.wada-ama.org/sites/default/files/2024-09/2025list_en_final_clean_12_september_2024.pdf

[31] Simoni RE, Scalco FB, de Oliveira MLC, Aquino Neto FR. Plasma volume expanders: use in medicine and detecting misuse in sports. Bioanalysis. 2011;3:215–26. 10.4155/bio.10.181. 21250849 10.4155/bio.10.181

[32] Yamazaki Y, Morita T. Molecular and functional diversity of vascular endothelial growth factors. Mol Divers. 2006;10:515–27. 10.1007/s11030-006-9027-3. 16972015 10.1007/s11030-006-9027-3

[33] Tjwa M, Luttun A, Autiero M, Carmeliet P. VEGF and PlGF: two pleiotropic growth factors with distinct roles in development and homeostasis. Cell Tissue Res. 2003;314:5–14. 10.1007/s00441-003-0776-3. 13680354 10.1007/s00441-003-0776-3

[34] Baoutina A. Performance enhancement by gene doping. Genet Mol Asp Sport Perform. 2010;373–82. 10.1002/9781444327335.ch32.

[35] Haisma HJ, De Hon O. Gene doping. Int J Sports Med. 2006;27:257–66. 10.1055/s-2006-923986. 16572366 10.1055/s-2006-923986

[36] Krych K, Goździcka-Józefïak A. Doping in sport: new developments. Hum Mov. 2008;9:62–75. 10.2478/v10038-008-0009-4.

[37] Oliveira RS, Collares TF, Smith KR, Collares TV, Seixas FK. The use of genes for performance enhancement: doping or therapy? Braz J Med Biol Res. 2011;44:1194–201. 10.1590/s0100-879x2011007500145. 22030863 10.1590/s0100-879x2011007500145

[38] van der Gronde T, De Hon O, Haisma HJ, Pieters T. Gene doping: an overview and current implications for athletes. Br J Sports Med. 2013;47:670–8. 10.1136/bjsports-2012-091288. 23322893 10.1136/bjsports-2012-091288

[39] Choi K, Kennedy M, Kazarov A, Papadimitriou JC, Keller G. A common precursor for hematopoietic and endothelial cells. Development. 1998;125:725–32. 10.1242/dev.125.4.725. 9435292 10.1242/dev.125.4.725

[40] Ferrara N, Gerber H-P, LeCouter J. The biology of VEGF and its receptors. Nat Med. 2003;9:669–76. 10.1038/nm0603-669. 12778165 10.1038/nm0603-669

[41] Eichmann A, Corbel C, Nataf V, Vaigot P, Bréant C, Le Douarin NM. Ligand-dependent development of the endothelial and hemopoietic lineages from embryonic mesodermal cells expressing vascular endothelial growth factor receptor 2. Proc Natl Acad Sci U S A. 1997;94:5141–6. 10.1073/pnas.94.10.5141. 9144204 10.1073/pnas.94.10.5141 PMC24645

[42] Ferrara N, Carver-Moore K, Chen H, Dowd M, Lu L, O’Shea KS, et al. Heterozygous embryonic lethality induced by targeted inactivation of the VEGF gene. Nature. 1996;380:439–42. 10.1038/380439a0. 8602242 10.1038/380439a0

[43] Carmeliet P, Ferreira V, Breier G, Pollefeyt S, Kieckens L, Gertsenstein M, et al. Abnormal blood vessel development and lethality in embryos lacking a single VEGF allele. Nature. 1996;380:435–9. 10.1038/380435a0. 8602241 10.1038/380435a0

[44] Fang S, Chen S, Nurmi H, Leppänen V-M, Jeltsch M, Scadden D, et al. VEGF-C protects the integrity of the bone marrow perivascular niche in mice. Blood. 2020;136:1871–83. 10.1182/blood.2020005699. 32842144 10.1182/blood.2020005699 PMC7568034

[45] Sekiguchi K, Ito Y, Hattori K, Inoue T, Hosono K, Honda M, et al. VEGF receptor 1-expressing macrophages recruited from bone marrow enhances angiogenesis in endometrial tissues. Sci Rep. 2019;9:7037. 10.1038/s41598-019-43185-8. 31065021 10.1038/s41598-019-43185-8 PMC6504918

[46] Kukk E, Lymboussaki A, Taira S, Kaipainen A, Jeltsch M, Joukov V, et al. VEGF-C receptor binding and pattern of expression with VEGFR-3 suggests a role in lymphatic vascular. Development. 1996;122:3829–337. 10.1242/dev.122.12.3829. 9012504 10.1242/dev.122.12.3829

[47] Jeltsch M, Kaipainen A, Joukov V, Meng X, Lakso M, Rauvala H, et al. Hyperplasia of lymphatic vessels in VEGF-C transgenic mice. Science. 1997;276:1423–5. 10.1126/science.276.5317.1423. 9162011 10.1126/science.276.5317.1423

[48] Karkkainen MJ, Haiko P, Sainio K, Partanen J, Taipale J, Petrova TV, et al. Vascular endothelial growth factor C is required for sprouting of the first lymphatic vessels from embryonic veins. Nat Immunol. 2004;5:74–80. 10.1038/ni1013. 14634646 10.1038/ni1013

[49] Mandriota SJ, Jussila L, Jeltsch M, Compagni A, Baetens D, Prevo R. Vascular endothelial growth factor-C-mediated lymphangiogenesis promotes tumour metastasis. EMBO J. 2001;20:672–82. 10.1093/emboj/20.4.672. 11179212 10.1093/emboj/20.4.672 PMC145430

[50] Skobe M, Hawighorst T, Jackson DG, Prevo R, Janes L, Velasco P, et al. Induction of tumor lymphangiogenesis by VEGF-C promotes breast cancer metastasis. Nat Med. 2001;7:192–8. 10.1038/84643. 11175850 10.1038/84643

[51] Karpanen T, Egeblad M, Karkkainen MJ, Kubo H, Ylä-Herttuala S, Jäättelä M, et al. Vascular Endothelial Growth Factor C Promotes Tumor Lymphangiogenesis and Intralymphatic Tumor Growth. Cancer Res. 2001;61:1786–90. https://cancerres.aacrjournals.org/content/61/5/1786. 11280723

[52] Saaristo A, Tammela T, Timonen J, Yla-Herttuala S, Tukiainen E, Asko-Seljavaara S, et al. Vascular endothelial growth factor-C gene therapy restores lymphatic flow across incision wounds. FASEB J. 2004;18:1707–9. 10.1096/fj.04-1592fje. 15361472 10.1096/fj.04-1592fje

[53] Senger DR, Galli SJ, Dvorak AM, Perruzzi CA, Harvey VS, Dvorak HF. Tumor cells secrete a vascular permeability factor that promotes accumulation of ascites fluid. Science. 1983;219:983–5. 10.1126/science.6823562. 6823562 10.1126/science.6823562

[54] Leung DW, Cachianes G, Kuang WJ, Goeddel DV, Ferrara N. Vascular endothelial growth factor is a secreted angiogenic mitogen. Science. 1989;246:1306–9. 10.1126/science.2479986. 2479986 10.1126/science.2479986

[55] Künnapuu J, Bokharaie H, Jeltsch M. Proteolytic cleavages in the VEGF family: generating diversity among angiogenic VEGFs, essential for the activation of lymphangiogenic VEGFs. Biology. 2021;10:167. 10.3390/biology10020167. 33672235 10.3390/biology10020167 PMC7926383

[56] Lee C, Kim M-J, Kumar A, Lee H-W, Yang Y, Kim Y. Vascular endothelial growth factor signaling in health and disease: from molecular mechanisms to therapeutic perspectives. Signal Transduct Target Ther. 2025;10:170. 10.1038/s41392-025-02249-0. 40383803 10.1038/s41392-025-02249-0 PMC12086256

[57] Maglione D, Guerriero V, Viglietto G, Delli-Bovi P, Persico MG. Isolation of a human placenta cDNA coding for a protein related to the vascular permeability factor. Proc Natl Acad Sci U S A. 1991;88:9267–71. 10.1073/pnas.88.20.9267. 1924389 10.1073/pnas.88.20.9267 PMC52695

[58] Gladstone RA, Snelgrove JW, McLaughlin K, Hobson SR, Windrim RC, Melamed N, et al. Placental growth factor (PlGF) and soluble fms-like tyrosine kinase-1 (sFlt1): powerful new tools to guide obstetric and medical care in pregnancy. Obstet Med. 2025. 10.1177/1753495X251327462. 40191640 10.1177/1753495X251327462 PMC11969481

[59] Olofsson B, Pajusola K, Kaipainen A, von Euler G, Joukov V, Saksela O, et al. Vascular endothelial growth factor B, a novel growth factor for endothelial cells. Proc Natl Acad Sci U S A. 1996;93:2576–81. 10.1073/pnas.93.6.2576. 8637916 10.1073/pnas.93.6.2576 PMC39839

[60] Joukov V, Pajusola K, Kaipainen A, Chilov D, Lahtinen I, Kukk E, et al. A novel vascular endothelial growth factor, VEGF-C, is a ligand for the Flt4 (VEGFR-3) and KDR (VEGFR-2) receptor tyrosine kinases. EMBO J. 1996;15(2):290–8. 8617204 PMC449944

[61] Lee J, Gray A, Yuan J, Luoh SM, Avraham H, Wood WI. Vascular endothelial growth factor-related protein: a ligand and specific activator of the tyrosine kinase receptor Flt4. Proc Natl Acad Sci U S A. 1996;93:1988–92. 10.1073/pnas.93.5.1988. 8700872 10.1073/pnas.93.5.1988 PMC39896

[62] Kuonqui KG, Campbell A-C, Pollack BL, Shin J, Sarker A, Brown S, et al. Regulation of VEGFR3 signaling in lymphatic endothelial cells. Front Cell Dev Biol. 2025;13:1527971. 10.3389/fcell.2025.1527971. 40046235 10.3389/fcell.2025.1527971 PMC11880633

[63] Rauniyar K, Jha SK, Jeltsch M. Biology of Vascular Endothelial Growth Factor C in the morphogenesis of lymphatic vessels. Front Bioeng Biotechnol. 2018;6:7. 10.3389/fbioe.2018.00007. 29484295 10.3389/fbioe.2018.00007 PMC5816233

[64] Achen MG, Jeltsch M, Kukk E, Mäkinen T, Vitali A, Wilks AF, et al. Vascular endothelial growth factor D (VEGF-D) is a ligand for the tyrosine kinases VEGF receptor 2 (Flk1) and VEGF receptor 3 (Flt4). Proc Natl Acad Sci U S A. 1998;95:548–53. 10.1073/pnas.95.2.548. 9435229 10.1073/pnas.95.2.548 PMC18457

[65] Orlandini M, Marconcini L, Ferruzzi R, Oliviero S. Identification of a c-fos-induced gene that is related to the platelet-derived growth factor/vascular endothelial growth factor family. Proc Natl Acad Sci U S A. 1996;93:11675–80. 10.1073/pnas.93.21.11675. 8876195 10.1073/pnas.93.21.11675 PMC38117

[66] Yamada Y, Nezu J, Shimane M, Hirata Y. Molecular cloning of a novel vascular endothelial growth factor, VEGF-D. Genomics. 1997;42:483–8. 10.1006/geno.1997.4774. 9205122 10.1006/geno.1997.4774

[67] Bokhari SMZ, Hamar P. Vascular endothelial growth factor-D (VEGF-D): an angiogenesis bypass in malignant tumors. Int J Mol Sci. 2023;24:13317. 10.3390/ijms241713317. 37686121 10.3390/ijms241713317 PMC10487419

[68] Stacker SA, Achen MG. Emerging roles for VEGF-D in human disease. Biomolecules. 2018. 10.3390/biom8010001. 29300337 10.3390/biom8010001 PMC5871970

[69] Lyttle DJ, Fraser KM, Fleming SB, Mercer AA, Robinson AJ. Homologs of vascular endothelial growth factor are encoded by the poxvirus orf virus. J Virol. 1994;68:84–92. 10.1128/JVI.68.1.84-92.1994. 8254780 10.1128/jvi.68.1.84-92.1994 PMC236267

[70] Komori Y, Nikai T, Taniguchi K, Masuda K, Sugihara H. Vascular endothelial growth factor VEGF-like heparin-binding protein from the venom of *Vipera aspis* (Aspic viper). Biochemistry. 1999;38:11796–803. 10.1021/bi990562z. 10512636 10.1021/bi990562z

[71] Yamazaki Y, Matsunaga Y, Tokunaga Y, Obayashi S, Saito M, Morita T. Snake venom vascular endothelial growth factors (VEGF-Fs) exclusively vary their structures and functions among species. J Biol Chem. 2009;284:9885–91. 10.1074/jbc.M809071200. 19208624 10.1074/jbc.M809071200 PMC2665111

[72] Nagy JA, Vasile E, Feng D, Sundberg C, Brown LF, Detmar MJ, et al. Vascular Permeability Factor/Vascular Endothelial Growth Factor induces lymphangiogenesis as well as angiogenesis. J Exp Med. 2002;196:1497–506. 10.1084/jem.20021244. 12461084 10.1084/jem.20021244 PMC2194262

[73] Halin C, Tobler NE, Vigl B, Brown LF, Detmar M. VEGF-A produced by chronically inflamed tissue induces lymphangiogenesis in draining lymph nodes. Blood. 2007;110:3158–67. 10.1182/blood-2007-01-066811. 17625067 10.1182/blood-2007-01-066811 PMC2200913

[74] Anisimov A, Leppanen V-M, Tvorogov D, Zarkada G, Jeltsch M, Holopainen T, et al. The basis for the distinct biological activities of vascular endothelial growth factor receptor-1 ligands. Sci Signal. 2013. 10.1126/scisignal.2003905. 23821770 10.1126/scisignal.2003905

[75] Bry M, Kivelä R, Holopainen T, Anisimov A, Tammela T, Soronen J, et al. Vascular Endothelial Growth Factor-B acts as a coronary growth factor in transgenic rats without inducing angiogenesis, vascular leak, or inflammation. Circulation. 2010;122:1725–33. 10.1161/CIRCULATIONAHA.110.957332. 20937974 10.1161/CIRCULATIONAHA.110.957332

[76] Li X, Tjwa M, Van Hove I, Enholm B, Neven E, Paavonen K, et al. Reevaluation of the role of VEGF-B suggests a restricted role in the revascularization of the ischemic myocardium. Arterioscler Thromb Vasc Biol. 2008;28:1614–20. 10.1161/ATVBAHA.107.158725. 18511699 10.1161/ATVBAHA.107.158725 PMC2753879

[77] Joukov V, Sorsa T, Kumar V, Jeltsch M, Claesson-Welsh L, Cao Y, et al. Proteolytic processing regulates receptor specificity and activity of VEGF-C. EMBO J. 1997;16:3898–911. 10.1093/emboj/16.13.3898. 9233800 10.1093/emboj/16.13.3898 PMC1170014

[78] Stacker SA, Stenvers K, Caesar C, Vitali A, Domagala T, Nice E, et al. Biosynthesis of Vascular Endothelial Growth Factor-D involves proteolytic processing which generates non-covalent homodimers. J Biol Chem. 1999;274:32127–36. 10.1074/jbc.274.45.32127. 10542248 10.1074/jbc.274.45.32127

[79] Rissanen TT, Markkanen JE, Gruchala M, Heikura T, Puranen A, Kettunen MI, et al. VEGF-D is the strongest angiogenic and lymphangiogenic effector among VEGFs delivered into skeletal muscle via adenoviruses. Circ Res. 2003;92:1098–106. 10.1161/01.RES.0000073584.46059.E3. 12714562 10.1161/01.RES.0000073584.46059.E3

[80] Rutanen J, Rissanen TT, Markkanen JE, Gruchala M, Silvennoinen P, Kivelä A, et al. Adenoviral Catheter-Mediated Intramyocardial Gene Transfer Using the Mature Form of Vascular Endothelial Growth Factor-D Induces Transmural Angiogenesis in Porcine Heart. Circulation. 2004;109:1029–35. 10.1161/01.CIR.0000115519.03688.A2. 14967735 10.1161/01.CIR.0000115519.03688.A2

[81] Zhang F, Tang Z, Hou X, Lennartsson J, Li Y, Koch AW, et al. VEGF-B is dispensable for blood vessel growth but critical for their survival, and VEGF-B targeting inhibits pathological angiogenesis. Proc Natl Acad Sci U S A. 2009;106:6152–7. 10.1073/pnas.0813061106. 19369214 10.1073/pnas.0813061106 PMC2669337

[82] Ardakanizade M. The effects of mid- and long-term endurance exercise on heart angiogenesis and oxidative stress. Iran J Basic Med Sci. 2018;21:800–5. 10.22038/IJBMS.2018.27211.6814. 30186566 10.22038/IJBMS.2018.27211.6814 PMC6118080

[83] Lieu CH, Tran H, Jiang Z-Q, Mao M, Overman MJ, Lin E, et al. The Association of Alternate VEGF ligands with resistance to anti-VEGF therapy in metastatic colorectal cancer. PLoS ONE. 2013;8:e77117. 10.1371/journal.pone.0077117. 24143206 10.1371/journal.pone.0077117 PMC3797099

[84] Carmeliet P, Li X, Treps L, Conradi L-C, Loges S. RAISEing VEGF-D’s importance as predictive biomarker for ramucirumab in metastatic colorectal cancer patients. Ann Oncol. 2018;29:529–32. 10.1093/annonc/mdy028. 32139029 10.1093/annonc/mdy028

[85] Park JE, Chen HH, Winer J, Houck KA, Ferrara N. Placenta growth factor. Potentiation of vascular endothelial growth factor bioactivity, in vitro and in vivo, and high affinity binding to Flt-1 but not to Flk-1/KDR. J Biol Chem. 1994;269:25646–54. 7929268

[86] Luttun A, Tjwa M, Moons L, Wu Y, Angelillo-Scherrer A, Liao F, et al. Revascularization of ischemic tissues by PlGF treatment, and inhibition of tumor angiogenesis, arthritis and atherosclerosis by anti-Flt1. Nat Med. 2002;8:831–40. 10.1038/nm731. 12091877 10.1038/nm731

[87] Baldwin ME, Halford MM, Roufail S, Williams RA, Hibbs ML, Grail D, et al. Vascular endothelial growth factor D is dispensable for development of the lymphatic system. Mol Cell Biol. 2005;25:2441–9. 10.1128/MCB.25.6.2441-2449.2005. 15743836 10.1128/MCB.25.6.2441-2449.2005 PMC1061605

[88] Bellomo D, Headrick JP, Silins GU, Paterson CA, Thomas PS, Gartside M, et al. Mice lacking the vascular endothelial growth factor-B gene (*Vegfb*) have smaller hearts, dysfunctional coronary vasculature, and impaired recovery from cardiac ischemia. Circ Res. 2000;86:E29–35. 10.1161/01.res.86.2.e29. 10666423 10.1161/01.res.86.2.e29

[89] Aase K, von Euler G, Li X, Pontén A, Thorén P, Cao R, et al. Vascular endothelial growth factor-B–deficient mice display an atrial conduction defect. Circulation. 2001;104:358–64. 10.1161/01.CIR.104.3.358. 11457758 10.1161/01.cir.104.3.358

[90] Wanstall JC, Gambino A, Jeffery TK, Cahill MM, Bellomo D, Hayward NK, et al. Vascular endothelial growth factor-B-deficient mice show impaired development of hypoxic pulmonary hypertension. Cardiovasc Res. 2002;55:361–8. 10.1016/S0008-6363(02)00440-6. 12123775 10.1016/s0008-6363(02)00440-6

[91] Apicella I, Cicatiello V, Acampora D, Tarallo V, Falco SD. Full functional knockout of placental growth factor by knockin with an inactive variant able to heterodimerize with VEGF-A. Cell Rep. 2018;23:3635–46. 10.1016/j.celrep.2018.05.067. 29925004 10.1016/j.celrep.2018.05.067

[92] Cheung E, Myers K, Yancopoulos GD, Lobov IB, Wiegand SJ. Placental growth factor (PlGF) deficient mice show delays in normal retinal vascular development and reduced vascular abnormalities in oxygen-induced retinal ischemia. Invest Ophthalmol Vis Sci. 2009;50:2943.

[93] Parchem JG, Kanasaki K, Kanasaki M, Sugimoto H, Xie L, Hamano Y, et al. Loss of placental growth factor ameliorates maternal hypertension and preeclampsia in mice. J Clin Invest. 2018;128:5008–17. 10.1172/JCI99026. 30179860 10.1172/JCI99026 PMC6205389

[94] Oura H, Bertoncini J, Velasco P, Brown LF, Carmeliet P, Detmar M. A critical role of placental growth factor in the induction of inflammation and edema formation. Blood. 2003;101:560–7. 10.1182/blood-2002-05-1516. 12393422 10.1182/blood-2002-05-1516

[95] Huez I, Créancier L, Audigier S, Gensac MC, Prats AC, Prats H. Two independent internal ribosome entry sites are involved in translation initiation of vascular endothelial growth factor mRNA. Mol Cell Biol. 1998;18:6178–90. 10.1128/MCB.18.11.6178. 9774635 10.1128/mcb.18.11.6178 PMC109205

[96] Stein I, Itin A, Einat P, Skaliter R, Grossman Z, Keshet E. Translation of vascular endothelial growth factor mRNA by internal ribosome entry: implications for translation under hypoxia. Mol Cell Biol. 1998;18:3112–9. 10.1128/MCB.18.6.3112. 9584152 10.1128/mcb.18.6.3112 PMC108893

[97] Pagès G, Pouysségur J. Transcriptional regulation of the Vascular Endothelial Growth Factor gene–a concert of activating factors. Cardiovasc Res. 2005;65:564–73. 10.1016/j.cardiores.2004.09.032. 15664382 10.1016/j.cardiores.2004.09.032

[98] Arcondéguy T, Lacazette E, Millevoi S, Prats H, Touriol C. VEGF-A mRNA processing, stability and translation: a paradigm for intricate regulation of gene expression at the post-transcriptional level. Nucleic Acids Res. 2013;41:7997–8010. 10.1093/nar/gkt539. 23851566 10.1093/nar/gkt539 PMC3783158

[99] Detmar M, Yeo KT, Nagy JA, Van de Water L, Brown LF, Berse B, et al. Keratinocyte-derived vascular permeability factor (vascular endothelial growth factor) is a potent mitogen for dermal microvascular endothelial cells. J Invest Dermatol. 1995;105:44–50. 10.1111/1523-1747.ep12312542 10.1111/1523-1747.ep12312542 7615975

[100] Brown LF, Yeo KT, Berse B, Yeo TK, Senger DR, Dvorak HF, et al. Expression of vascular permeability factor (vascular endothelial growth factor) by epidermal keratinocytes during wound healing. J Exp Med. 1992;176:1375–9. 10.1084/jem.176.5.1375. 1402682 10.1084/jem.176.5.1375 PMC2119412

[101] Gustafsson T, Ameln H, Fischer H, Sundberg CJ, Timmons JA, Jansson E. VEGF-A splice variants and related receptor expression in human skeletal muscle following submaximal exercise. J Appl Physiol. 2005;98:2137–46. 10.1152/japplphysiol.01402.2004. 15661835 10.1152/japplphysiol.01402.2004

[102] Richardson RS, Wagner H, Mudaliar SRD, Saucedo E, Henry R, Wagner PD. Exercise adaptation attenuates VEGF gene expression in human skeletal muscle. Am J Physiol-Heart Circ Physiol. 2000;279:H772–8. 10.1152/ajpheart.2000.279.2.H772. 10924077 10.1152/ajpheart.2000.279.2.H772

[103] Bao P, Kodra A, Tomic-Canic M, Golinko MS, Ehrlich HP, Brem H. The role of vascular endothelial growth factor in wound healing. J Surg Res. 2009;153:347–58. 10.1016/j.jss.2008.04.023. 19027922 10.1016/j.jss.2008.04.023 PMC2728016

[104] Yano K, Brown LF, Detmar M. Control of hair growth and follicle size by VEGF-mediated angiogenesis. J Clin Invest. 2001;107:409–17. 10.1172/JCI11317. 11181640 10.1172/JCI11317 PMC199257

[105] Sugino N, Kashida S, Karube-Harada A, Takiguchi S, Kato H. Expression of vascular endothelial growth factor (VEGF) and its receptors in human endometrium throughout the menstrual cycle and in early pregnancy. Reproduction. 2002. 10.1530/rep.0.1230379. 11882015 10.1530/rep.0.1230379

[106] Dong Q, Cheng Z. Functions of VEGF in female reproductive system. Chin Sci Bull. 2003;48:217–22. 10.1007/BF03183286.

[107] Inoue M, Itoh H, Ueda M, Naruko T, Kojima A, Komatsu R, et al. Vascular Endothelial Growth Factor (VEGF) expression in human coronary atherosclerotic lesions: possible pathophysiological significance of VEGF in progression of atherosclerosis. Circulation. 1998;98:2108–16. 10.1161/01.CIR.98.20.2108. 9815864 10.1161/01.cir.98.20.2108

[108] Yano K, Liaw PC, Mullington JM, Shih S-C, Okada H, Bodyak N, et al. Vascular endothelial growth factor is an important determinant of sepsis morbidity and mortality. J Exp Med. 2006;203:1447–58. 10.1084/jem.20060375. 16702604 10.1084/jem.20060375 PMC2118329

[109] Scaldaferri F, Vetrano S, Sans M, Arena V, Straface G, Stigliano E, et al. VEGF-A links angiogenesis and inflammation in inflammatory bowel disease pathogenesis. Gastroenterology. 2009;136:585-595.e5. 10.1053/j.gastro.2008.09.064. 19013462 10.1053/j.gastro.2008.09.064

[110] Kang Y, Li H, Liu Y, Li Z. Regulation of VEGF-A expression and VEGF-A-targeted therapy in malignant tumors. J Cancer Res Clin Oncol. 2024;150:221. 10.1007/s00432-024-05714-5. 38687357 10.1007/s00432-024-05714-5 PMC11061008

[111] Ramakrishnan S, Anand V, Roy S. Vascular Endothelial growth factor signaling in hypoxia and Inflammation. J Neuroimmune Pharmacol. 2014;9:142–60. 10.1007/s11481-014-9531-7. 24610033 10.1007/s11481-014-9531-7 PMC4048289

[112] Ristimäki A, Narko K, Enholm B, Joukov V, Alitalo K. Proinflammatory cytokines regulate expression of the lymphatic endothelial mitogen vascular endothelial growth factor-C. J Biol Chem. 1998;273:8413–8. 10.1074/jbc.273.14.8413. 9525952 10.1074/jbc.273.14.8413

[113] Morfoisse F, Kuchnio A, Frainay C, Gomez-Brouchet A, Delisle M-B, Marzi S, et al. Hypoxia induces VEGF-C expression in metastatic tumor cells via a HIF-1α-independent translation-mediated mechanism. Cell Rep. 2014;6:155–67. 10.1016/j.celrep.2013.12.011. 24388748 10.1016/j.celrep.2013.12.011

[114] Okada K, Osaki M, Araki K, Ishiguro K, Ito H, Ohgi S. Expression of Hypoxia-inducible Factor (HIF-1α), VEGF-C and VEGF-D in non-invasive and invasive breast ductal carcinomas. Anticancer Res. 2005;25:3003–9. 16080559

[115] Forsythe JA, Jiang BH, Iyer NV, Agani F, Leung SW, Koos RD, et al. Activation of vascular endothelial growth factor gene transcription by hypoxia-inducible factor 1. Mol Cell Biol. 1996;16:4604–13. 10.1128/MCB.16.9.4604. 8756616 10.1128/mcb.16.9.4604 PMC231459

[116] Semenza GL. Hypoxia-Inducible Factor 1: control of oxygen homeostasis in health and disease. Pediatr Res. 2001;49:614–7. 10.1203/00006450-200105000-00002. 11328942 10.1203/00006450-200105000-00002

[117] Shweiki D, Itin A, Soffer D, Keshet E. Vascular endothelial growth factor induced by hypoxia may mediate hypoxia-initiated angiogenesis. Nature. 1992;359:843–5. 10.1038/359843a0. 1279431 10.1038/359843a0

[118] Levy AP, Levy NS, Wegner S, Goldberg MA. Transcriptional regulation of the rat vascular endothelial growth factor gene by hypoxia. J Biol Chem. 1995;270:13333–40. 10.1074/jbc.270.22.13333. 7768934 10.1074/jbc.270.22.13333

[119] Liu Y, Cox SR, Morita T, Kourembanas S. Hypoxia regulates vascular endothelial growth factor gene expression in endothelial cells. Identification of a 5’ enhancer. Circ Res. 1995;77:638–43. 10.1161/01.res.77.3.638. 7641334 10.1161/01.res.77.3.638

[120] Huang LE, Bunn HF. Hypoxia-inducible factor and its biomedical relevance. J Biol Chem. 2003;278:19575–8. 10.1074/jbc.R200030200. 12639949 10.1074/jbc.R200030200

[121] Hamanaka RB, Chandel NS. Mitochondrial reactive oxygen species regulate hypoxic signaling. Curr Opin Cell Biol. 2009;21:894–9. 10.1016/j.ceb.2009.08.005. 19781926 10.1016/j.ceb.2009.08.005 PMC2787901

[122] Waltenberger J, Claesson-Welsh L, Siegbahn A, Shibuya M, Heldin CH. Different signal transduction properties of KDR and Flt1, two receptors for vascular endothelial growth factor. J Biol Chem. 1994;269:26988–95. 10.1016/S0021-9258(18)47116-5. 7929439

[123] Gille H, Kowalski J, Li B, LeCouter J, Moffat B, Zioncheck TF, et al. Analysis of biological effects and signaling properties of Flt-1 (VEGFR-1) and KDR (VEGFR-2): a reassessment using novel receptor-specific vascular endothelial growth factor mutants. J Biol Chem. 2001;276:3222–30. 10.1074/jbc.M002016200. 11058584 10.1074/jbc.M002016200

[124] Sun Z, Li X, Massena S, Kutschera S, Padhan N, Gualandi L, et al. VEGFR2 induces c-Src signaling and vascular permeability in vivo via the adaptor protein TSAd. J Exp Med. 2012;209:1363–77. 10.1084/jem.20111343. 22689825 10.1084/jem.20111343 PMC3405501

[125] Hiratsuka S, Minowa O, Kuno J, Noda T, Shibuya M. Flt-1 lacking the tyrosine kinase domain is sufficient for normal development and angiogenesis in mice. Proc Natl Acad Sci U S A. 1998;95:9349–54. 10.1073/pnas.95.16.9349. 9689083 10.1073/pnas.95.16.9349 PMC21341

[126] Hiratsuka S, Nakao K, Nakamura K, Katsuki M, Maru Y, Shibuya M. Membrane fixation of vascular endothelial growth factor receptor 1 ligand-binding domain is important for vasculogenesis and angiogenesis in mice. Mol Cell Biol. 2005;25:346–54. 10.1128/MCB.25.1.346-354.2005. 15601855 10.1128/MCB.25.1.346-354.2005 PMC538773

[127] Takahashi H, Shibuya M. The vascular endothelial growth factor (VEGF)/VEGF receptor system and its role under physiological and pathological conditions. Clin Sci Lond Engl 1979. 2005;109:227–41. 10.1042/CS20040370. 10.1042/CS20040370 16104843

[128] Kaipainen A, Korhonen J, Mustonen T, van Hinsbergh VW, Fang GH, Dumont D, et al. Expression of the fms-like tyrosine kinase 4 gene becomes restricted to lymphatic endothelium during development. Proc Natl Acad Sci U S A. 1995;92:3566–70. 10.1073/pnas.92.8.3566. 7724599 10.1073/pnas.92.8.3566 PMC42208

[129] Hamrah P, Chen L, Cursiefen C, Zhang Q, Joyce NC, Dana MR. Expression of vascular endothelial growth factor receptor-3 (VEGFR-3) on monocytic bone marrow-derived cells in the conjunctiva. Exp Eye Res. 2004;79:553–61. 10.1016/j.exer.2004.06.028. 15381039 10.1016/j.exer.2004.06.028

[130] Shibuya M. Differential roles of vascular endothelial growth factor receptor-1 and receptor-2 in angiogenesis. J Biochem Mol Biol. 2006;39:469–78. 10.5483/bmbrep.2006.39.5.469. 17002866 10.5483/bmbrep.2006.39.5.469

[131] Nowak G, Karrar A, Holmén C, Nava S, Uzunel M, Hultenby K, et al. Expression of vascular endothelial growth factor receptor-2 or tie-2 on peripheral blood cells defines functionally competent cell populations capable of reendothelialization. Circulation. 2004;110:3699–707. 10.1161/01.CIR.0000143626.16576.51. 15381639 10.1161/01.CIR.0000143626.16576.51

[132] Ishida A, Murray J, Saito Y, Kanthou C, Benzakour O, Shibuya M, et al. Expression of vascular endothelial growth factor receptors in smooth muscle cells. J Cell Physiol. 2001;188:359–68. 10.1002/jcp.1121. 11473363 10.1002/jcp.1121

[133] Liao XH, Xiang Y, Li H, Zheng DL, Xu Y, Xi Yu C, et al. VEGF-A stimulates STAT3 activity via nitrosylation of Myocardin to regulate the expression of vascular smooth muscle cell differentiation markers. Sci Rep. 2017;7:2660. 10.1038/s41598-017-02907-6. 28572685 10.1038/s41598-017-02907-6 PMC5453982

[134] Wittko-Schneider IM, Schneider FT, Plate KH. Brain homeostasis: VEGF receptor 1 and 2—two unequal brothers in mind. Cell Mol Life Sci. 2013;70:1705–25. 10.1007/s00018-013-1279-3. 23475067 10.1007/s00018-013-1279-3 PMC3632714

[135] Hou Y, Shin Y-J, Han EJ, Choi J-S, Park J-M, Cha J-H, et al. Distribution of vascular endothelial growth factor receptor-3/Flt4 mRNA in adult rat central nervous system. J Chem Neuroanat. 2011;42:56–64. 10.1016/j.jchemneu.2011.06.001. 21703344 10.1016/j.jchemneu.2011.06.001

[136] Ward MC, Cunningham AM. Developmental expression of vascular endothelial growth factor receptor 3 and vascular endothelial growth factor C in forebrain. Neuroscience. 2015;303:544–57. 10.1016/j.neuroscience.2015.04.063. 25943477 10.1016/j.neuroscience.2015.04.063

[137] Jeltsch M, Jha SK, Tvorogov D, Anisimov A, Leppänen V-M, Holopainen T, et al. CCBE1 enhances lymphangiogenesis via A Disintegrin and Metalloprotease With Thrombospondin Motifs-3–mediated vascular endothelial growth factor-C activation. Circulation. 2014;129:1962–71. 10.1161/CIRCULATIONAHA.113.002779. 24552833 10.1161/CIRCULATIONAHA.113.002779

[138] Wang G, Muhl L, Padberg Y, Dupont L, Peterson-Maduro J, Stehling M, et al. Specific fibroblast subpopulations and neuronal structures provide local sources of Vegfc-processing components during zebrafish lymphangiogenesis. Nat Commun. 2020;11:2724. 10.1038/s41467-020-16552-7. 32483144 10.1038/s41467-020-16552-7 PMC7264274

[139] Leppanen V-M, Jeltsch M, Anisimov A, Tvorogov D, Aho K, Kalkkinen N, et al. Structural determinants of vascular endothelial growth factor-D receptor binding and specificity. Blood. 2011;117:1507–15. 10.1182/blood-2010-08-301549. 21148085 10.1182/blood-2010-08-301549

[140] Jha SK, Rauniyar K, Chronowska E, Mattonet K, Maina EW, Koistinen H, et al. KLK3/PSA and cathepsin D activate VEGF-C and VEGF-D. Elife. 2019;8:e44478. 10.7554/eLife.44478. 31099754 10.7554/eLife.44478 PMC6588350

[141] Hoeben A, Landuyt B, Highley MS, Wildiers H, Van Oosterom AT, De Bruijn EA. Vascular endothelial growth factor and angiogenesis. Pharmacol Rev. 2004;56:549–80. 10.1124/pr.56.4.3. 15602010 10.1124/pr.56.4.3

[142] Melincovici CS, Boşca AB, Şuşman S, Mărginean M, Mihu C, Istrate M, et al. Vascular endothelial growth factor (VEGF) - key factor in normal and pathological angiogenesis. Rom J Morphol Embryol. 2018;59:455–67. 30173249

[143] Dvorak HF, Brown LF, Detmar M, Dvorak AM. Vascular permeability factor/vascular endothelial growth factor, microvascular hyperpermeability, and angiogenesis. Am J Pathol. 1995;146:1029–39. 7538264 PMC1869291

[144] Breen EC, Johnson EC, Wagner H, Tseng HM, Sung LA, Wagner PD. Angiogenic growth factor mRNA responses in muscle to a single bout of exercise. J Appl Physiol. 1996;81:355–61. 10.1152/jappl.1996.81.1.355. 8828685 10.1152/jappl.1996.81.1.355

[145] Verma M, Asakura Y, Wang X, Zhou K, Ünverdi M, Kann AP, et al. Endothelial cell signature in muscle stem cells validated by VEGFA-FLT1-AKT1 axis promoting survival of muscle stem cell. Elife. 2024;13:e73592. 10.7554/eLife.73592. 38842166 10.7554/eLife.73592 PMC11216748

[146] Chintalgattu V, Nair DM, Katwa LC. Cardiac myofibroblasts: a novel source of vascular endothelial growth factor (VEGF) and its receptors Flt-1 and KDR. J Mol Cell Cardiol. 2003;35:277–86. 10.1016/s0022-2828(03)00006-3. 12676542 10.1016/s0022-2828(03)00006-3

[147] Spinella F, Garrafa E, Di Castro V, Rosanò L, Nicotra MR, Caruso A, et al. Endothelin-1 stimulates lymphatic endothelial cells and lymphatic vessels to grow and invade. Cancer Res. 2009;69:2669–76. 10.1158/0008-5472.CAN-08-1879. 19276384 10.1158/0008-5472.CAN-08-1879

[148] Peach CJ, Mignone VW, Arruda MA, Alcobia DC, Hill SJ, Kilpatrick LE, et al. Molecular pharmacology of VEGF-A isoforms: binding and signalling at VEGFR2. Int J Mol Sci. 2018;19:1264. 10.3390/ijms19041264. 29690653 10.3390/ijms19041264 PMC5979509

[149] Gerber HP, Hillan KJ, Ryan AM, Kowalski J, Keller GA, Rangell L, et al. VEGF is required for growth and survival in neonatal mice. Development. 1999;126:1149–59. 10.1242/dev.126.6.1149. 10021335 10.1242/dev.126.6.1149

[150] Dumont DJ, Fong GH, Puri MC, Gradwohl G, Alitalo K, Breitman ML. Vascularization of the mouse embryo: a study of flk-1, tek, tie, and vascular endothelial growth factor expression during development. Dev Dyn. 1995;203:80–92. 10.1002/aja.1002030109. 7647376 10.1002/aja.1002030109

[151] Risau W, Flamme I. Vasculogenesis. Annu Rev Cell Dev Biol. 1995;11:73–91. 10.1146/annurev.cb.11.110195.000445. 8689573 10.1146/annurev.cb.11.110195.000445

[152] Janvier A, Nadeau S, Baribeau J, Perreault T. Role of vascular endothelial growth factor receptor 1 and vascular endothelial growth factor receptor 2 in the vasodilator response to vascular endothelial growth factor in the neonatal piglet lung. Crit Care Med. 2005;33:860–6. 10.1097/01.ccm.0000159563.97092.a7. 15818117 10.1097/01.ccm.0000159563.97092.a7

[153] Carmeliet P, Jain RK. Angiogenesis in cancer and other diseases. Nature. 2000;407:249–57. 10.1038/35025220. 11001068 10.1038/35025220

[154] Carmeliet P. VEGF as a key mediator of angiogenesis in cancer. Oncology. 2005;69(Suppl 3):4–10. 10.1159/000088478. 16301830 10.1159/000088478

[155] Adamis AP, Miller JW, Bernal MT, D’Amico DJ, Folkman J, Yeo TK, et al. Increased vascular endothelial growth factor levels in the vitreous of eyes with proliferative diabetic retinopathy. Am J Ophthalmol. 1994;118:445–50. 10.1016/s0002-9394(14)75794-0. 7943121 10.1016/s0002-9394(14)75794-0

[156] Aiello LP, Avery RL, Arrigg PG, Keyt BA, Jampel HD, Shah ST, et al. Vascular endothelial growth factor in ocular fluid of patients with diabetic retinopathy and other retinal disorders. N Engl J Med. 1994;331:1480–7. 10.1056/NEJM199412013312203. 7526212 10.1056/NEJM199412013312203

[157] Ikeda M, Hosoda Y, Hirose S, Okada Y, Ikeda E. Expression of vascular endothelial growth factor isoforms and their receptors Flt-1, KDR, and neuropilin-1 in synovial tissues of rheumatoid arthritis. J Pathol. 2000;191:426–33. 10.1002/1096-9896(2000)9999:9999<::AID-PATH649>3.0.CO;2-E. 10918218 10.1002/1096-9896(2000)9999:9999<::AID-PATH649>3.0.CO;2-E

[158] Enomoto H, Inoki I, Komiya K, Shiomi T, Ikeda E, Obata K, et al. Vascular endothelial growth factor isoforms and their receptors are expressed in human osteoarthritic cartilage. Am J Pathol. 2003;162:171–81. 10.1016/s0002-9440(10)63808-4. 12507900 10.1016/s0002-9440(10)63808-4 PMC1851114

[159] Ramos MA, Kuzuya M, Esaki T, Miura S, Satake S, Asai T, et al. Induction of macrophage VEGF in response to oxidized LDL and VEGF accumulation in human atherosclerotic lesions. Arterioscler Thromb Vasc Biol. 1998;18:1188–96. 10.1161/01.atv.18.7.1188. 9672081 10.1161/01.atv.18.7.1188

[160] Yang P-Y, Rui Y-C, Jin Y-X, Li T-J, Qiu Y, Zhang L, et al. Antisense oligodeoxynucleotide inhibits vascular endothelial growth factor expression in U937 foam cells. Acta Pharmacol Sin. 2003;24:610–4. 12791191

[161] Solomon SD, Lindsley K, Vedula SS, Krzystolik MG, Hawkins BS. Anti‐vascular endothelial growth factor for neovascular age‐related macular degeneration. Cochrane Database Syst Rev. 2019;2019:CD005139. 10.1002/14651858.CD005139.pub4. 10.1002/14651858.CD005139.pub4 PMC6419319 30834517

[162] Folkman J. Tumor angiogenesis: therapeutic implications. N Engl J Med. 1971;285:1182–6. 10.1056/NEJM197111182852108. 4938153 10.1056/NEJM197111182852108

[163] Hanahan D, Folkman J. Patterns and emerging mechanisms of the angiogenic switch during tumorigenesis. Cell. 1996;86:353–64. 10.1016/S0092-8674(00)80108-7. 8756718 10.1016/s0092-8674(00)80108-7

[164] Bergers G, Brekken R, McMahon G, Vu TH, Itoh T, Tamaki K, et al. Matrix metalloproteinase-9 triggers the angiogenic switch during carcinogenesis. Nat Cell Biol. 2000;2:737–44. 10.1038/35036374. 11025665 10.1038/35036374 PMC2852586

[165] Nagy JA, Chang S-H, Dvorak AM, Dvorak HF. Why are tumour blood vessels abnormal and why is it important to know? Br J Cancer. 2009;100:865–9. 10.1038/sj.bjc.6604929. 19240721 10.1038/sj.bjc.6604929 PMC2661770

[166] Ross M, Kargl CK, Ferguson R, Gavin TP, Hellsten Y. Exercise-induced skeletal muscle angiogenesis: impact of age, sex, angiocrines and cellular mediators. Eur J Appl Physiol. 2023;123:1415–32. 10.1007/s00421-022-05128-6. 36715739 10.1007/s00421-022-05128-6 PMC10276083

[167] Mølmen KS, Almquist NW, Skattebo Ø. Effects of exercise training on mitochondrial and capillary growth in human skeletal muscle: a systematic review and meta-regression. Sports Med. 2025;55:115–44. 10.1007/s40279-024-02120-2. 39390310 10.1007/s40279-024-02120-2 PMC11787188

[168] Lloyd PG, Prior BM, Li H, Yang HT, Terjung RL. VEGF receptor antagonism blocks arteriogenesis, but only partially inhibits angiogenesis, in skeletal muscle of exercise-trained rats. Am J Physiol Heart Circ Physiol. 2005;288:H759–68. 10.1152/ajpheart.00786.2004. 15471974 10.1152/ajpheart.00786.2004

[169] Gustafsson T, Puntschart A, Kaijser L, Jansson E, Sundberg CJ. Exercise-induced expression of angiogenesis-related transcription and growth factors in human skeletal muscle. Am J Physiol Heart Circ Physiol. 1999;276:H679–85. 10.1152/ajpheart.1999.276.2.H679. 10.1152/ajpheart.1999.276.2.H679 9950871

[170] Gustafsson T, Rundqvist H, Norrbom J, Rullman E, Jansson E, Sundberg CJ. The influence of physical training on the angiopoietin and VEGF-A systems in human skeletal muscle. J Appl Physiol. 2007;103:1012–20. 10.1152/japplphysiol.01103.2006. 17569764 10.1152/japplphysiol.01103.2006

[171] Lloyd PG, Prior BM, Yang HT, Terjung RL. Angiogenic growth factor expression in rat skeletal muscle in response to exercise training. Am J Physiol Heart Circ Physiol. 2003;284:H1668–78. 10.1152/ajpheart.00743.2002. 12543634 10.1152/ajpheart.00743.2002

[172] Eguchi R, Kawabe J, Wakabayashi I. VEGF-independent angiogenic factors: beyond VEGF/VEGFR2 signaling. J Vasc Res. 2022;59:78–89. 10.1159/000521584. 35152220 10.1159/000521584

[173] Hudlicka O, Brown MD. Adaptation of skeletal muscle microvasculature to increased or decreased blood flow: role of shear stress, nitric oxide and vascular endothelial growth factor. J Vasc Res. 2009;46:504–12. 10.1159/000226127. 19556804 10.1159/000226127

[174] Hellsten Y, Hoier B. Capillary growth in human skeletal muscle: physiological factors and the balance between pro-angiogenic and angiostatic factors. Biochem Soc Trans. 2014;42:1616–22. 10.1042/BST20140197. 25399579 10.1042/BST20140197

[175] Tzima E, Irani-Tehrani M, Kiosses WB, Dejana E, Schultz DA, Engelhardt B, et al. A mechanosensory complex that mediates the endothelial cell response to fluid shear stress. Nature. 2005;437:426–31. 10.1038/nature03952. 16163360 10.1038/nature03952

[176] Urner S, Planas‐Paz L, Hilger LS, Henning C, Branopolski A, Kelly‐Goss M, et al. Identification of ILK as a critical regulator of VEGFR3 signalling and lymphatic vascular growth. EMBO J. 2019. 10.15252/embj.201899322. 30518533 10.15252/embj.201899322 PMC6331728

[177] Milkiewicz M, Hudlicka O, Brown MD, Silgram H. Nitric oxide, VEGF, and VEGFR-2: interactions in activity-induced angiogenesis in rat skeletal muscle. Am J Physiol Heart Circ Physiol. 2005;289:H336–43. 10.1152/ajpheart.01105.2004. 15734877 10.1152/ajpheart.01105.2004

[178] Ameln H, Gustafsson T, Sundberg CJ, Okamoto K, Jansson E, Poellinger L, et al. Physiological activation of hypoxia inducible factor-1 in human skeletal muscle. FASEB J. 2005;19:1009–11. 10.1096/fj.04-2304fje. 15811877 10.1096/fj.04-2304fje

[179] Arany Z, Foo S-Y, Ma Y, Ruas JL, Bommi-Reddy A, Girnun G, et al. HIF-independent regulation of VEGF and angiogenesis by the transcriptional coactivator PGC-1α. Nature. 2008;451:1008–12. 10.1038/nature06613. 18288196 10.1038/nature06613

[180] Aragón-Vela J, Casuso RA. Effect of hypoxia-inducible factor 1 on vascular endothelial growth factor expression in exercised human skeletal muscle: a systematic review and meta-analysis. Am J Physiol Cell Physiol. 2025;329:C272–82. 10.1152/ajpcell.00297.2025. 40522862 10.1152/ajpcell.00297.2025

[181] Gavin TP. Basal and exercise-induced regulation of skeletal muscle capillarization. Exerc Sport Sci Rev. 2009;37:86. 10.1097/JES.0b013e31819c2e9b. 19305200 10.1097/JES.0b013e31819c2e9b PMC3836628

[182] Lindholm ME, Rundqvist H. Skeletal muscle hypoxia-inducible factor-1 and exercise. Exp Physiol. 2016;101:28–32. 10.1113/EP085318. 26391197 10.1113/EP085318

[183] Bellafiore M, Battaglia G, Bianco A, Palma A. Expression pattern of angiogenic factors in healthy heart in response to physical exercise intensity. Front Physiol. 2019. 10.3389/fphys.2019.00238. 30984008 10.3389/fphys.2019.00238 PMC6447665

[184] Høier B, Olsen K, Nyberg M, Bangsbo J, Hellsten Y. Contraction-induced secretion of VEGF from skeletal muscle cells is mediated by adenosine. Am J Physiol Heart Circ Physiol. 2010;299:H857–62. 10.1152/ajpheart.00082.2010. 20543089 10.1152/ajpheart.00082.2010

[185] Morland C, Andersson KA, Haugen ØP, Hadzic A, Kleppa L, Gille A, et al. Exercise induces cerebral VEGF and angiogenesis via the lactate receptor HCAR1. Nat Commun. 2017;8:15557. 10.1038/ncomms15557. 28534495 10.1038/ncomms15557 PMC5457513

[186] Adair TH. Growth regulation of the vascular system: an emerging role for adenosine. Am J Physiol Regul Integr Comp Physiol. 2005;289:R283–96. 10.1152/ajpregu.00840.2004. 16014444 10.1152/ajpregu.00840.2004

[187] Hellsten Y, Rufener N, Nielsen JJ, Høier B, Krustrup P, Bangsbo J. Passive leg movement enhances interstitial VEGF protein, endothelial cell proliferation, and eNOS mRNA content in human skeletal muscle. Am J Physiol Regul Integr Comp Physiol. 2008;294:R975–82. 10.1152/ajpregu.00677.2007. 18094062 10.1152/ajpregu.00677.2007

[188] Höffner L, Nielsen JJ, Langberg H, Hellsten Y. Exercise but not prostanoids enhance levels of vascular endothelial growth factor and other proliferative agents in human skeletal muscle interstitium. J Physiol. 2003;550:217–25. 10.1113/jphysiol.2002.037051. 12754306 10.1113/jphysiol.2002.037051 PMC2343029

[189] Hansen AH, Nielsen JJ, Saltin B, Hellsten Y. Exercise training normalizes skeletal muscle vascular endothelial growth factor levels in patients with essential hypertension. J Hypertens. 2010;28:1176. 10.1097/HJH.0b013e3283379120. 20179634 10.1097/HJH.0b013e3283379120

[190] Kraus RM, Stallings HW, Yeager RC, Gavin TP. Circulating plasma VEGF response to exercise in sedentary and endurance-trained men. J Appl Physiol. 2004;96:1445–50. 10.1152/japplphysiol.01031.2003. 14660505 10.1152/japplphysiol.01031.2003

[191] Yan Z, Okutsu M, Akhtar YN, Lira VA. Regulation of exercise-induced fiber type transformation, mitochondrial biogenesis, and angiogenesis in skeletal muscle. J Appl Physiol. 2011;110(1):264–74. 10.1152/japplphysiol.00993.2010. 21030673 10.1152/japplphysiol.00993.2010 PMC3253006

[192] Huonker M, Schmid A, Schmidt-Trucksäß A, Grathwohl D, Keul J. Size and blood flow of central and peripheral arteries in highly trained able-bodied and disabled athletes. J Appl Physiol. 2003;95:685–91. 10.1152/japplphysiol.00710.2001. 12433857 10.1152/japplphysiol.00710.2001

[193] Egginton S. Invited review: activity-induced angiogenesis. Pflugers Arch Eur J Physiol. 2009;457:963–77. 10.1007/s00424-008-0563-9. 18704490 10.1007/s00424-008-0563-9

[194] Kambouris M, Ntalouka F, Ziogas G, Maffulli N. Predictive genomics DNA profiling for athletic performance. Recent Pat DNA Gene Seq. 2012;6:229–39. 10.2174/187221512802717321. 22827597 10.2174/187221512802717321

[195] Prior SJ, Hagberg JM, Paton CM, Douglass LW, Brown MD, McLenithan JC, et al. DNA sequence variation in the promoter region of the VEGF gene impacts VEGF gene expression and maximal oxygen consumption. Am J Physiol-Heart Circ Physiol. 2006;290:H1848–55. 10.1152/ajpheart.01033.2005. 16339827 10.1152/ajpheart.01033.2005

[196] Tsurumi Y, Takeshita S, Chen D, Kearney M, Rossow ST, Passeri J, et al. Direct intramuscular gene transfer of naked DNA encoding vascular endothelial growth factor augments collateral development and tissue perfusion. Circulation. 1996;94:3281–90. 10.1161/01.CIR.94.12.3281. 8989142 10.1161/01.cir.94.12.3281

[197] Baumgartner I, Pieczek A, Manor O, Blair R, Kearney M, Walsh K, et al. Constitutive expression of phVEGF165 after intramuscular gene transfer promotes collateral vessel development in patients with critical limb ischemia. Circulation. 1998;97:1114–23. 10.1161/01.CIR.97.12.1114. 9537336 10.1161/01.cir.97.12.1114

[198] Asahara T, Bauters C, Zheng LP, Takeshita S, Bunting S, Ferrara N, et al. Synergistic effect of vascular endothelial growth factor and basic fibroblast growth factor on angiogenesis in vivo. Circulation. 1995;92:365–71. 10.1161/01.CIR.92.9.365. 10.1161/01.cir.92.9.365 7586439

[199] Yeh JL, Giordano FJ. Gene-based therapeutic angiogenesis. Semin Thorac Cardiovasc Surg. 2003;15:236–49. 10.1016/S1043-0679(03)70003-3. 12973701 10.1016/s1043-0679(03)70003-3

[200] Ylä-Herttuala S, Bridges C, Katz MG, Korpisalo P. Angiogenic gene therapy in cardiovascular diseases: dream or vision? Eur Heart J. 2017;38:1365–71. 10.1093/eurheartj/ehw547. 28073865 10.1093/eurheartj/ehw547 PMC5837788

[201] Romeo FJ, Mavropoulos SA, Ishikawa K. Progress in clinical gene therapy for cardiac disorders. Mol Diagn Ther. 2023;27:179–91. 10.1007/s40291-022-00632-z. 36641770 10.1007/s40291-022-00632-z PMC10023344

[202] Leikas AJ, Hartikainen JEK, Kastrup J, Mathur A, Gyöngyösi M, Fernández-Avilés F, et al. Clinical development and proof of principle testing of new regenerative vascular endothelial growth factor-d therapy for refractory angina: rationale and design of the phase 2 ReGenHeart trial. Open Heart. 2024;11:e002817. 10.1136/openhrt-2024-002817. 39424303 10.1136/openhrt-2024-002817 PMC11487854

[203] Hartikainen J, Hassinen I, Hedman A, Kivelä A, Saraste A, Knuuti J, et al. Adenoviral intramyocardial VEGF-DΔNΔC gene transfer increases myocardial perfusion reserve in refractory angina patients: a phase I/IIa study with 1-year follow-up. Eur Heart J. 2017;38:2547–55. 10.1093/eurheartj/ehx352. 28903476 10.1093/eurheartj/ehx352 PMC5837555

[204] Leikas AJ, Hassinen I, Hedman A, Kivelä A, Ylä-Herttuala S, Hartikainen JEK. Long-term safety and efficacy of intramyocardial adenovirus-mediated VEGF-DΔNΔC gene therapy eight-year follow-up of phase I KAT301 study. Gene Ther. 2022;29:289–93. 10.1038/s41434-021-00295-1. 34593990 10.1038/s41434-021-00295-1 PMC9159942

[205] Naumann N, Paßreiter A, Thomas A, Krug O, Walpurgis K, Thevis M. Analysis of potential gene doping preparations for transgenic DNA in the context of sports drug testing programs. Int J Mol Sci. 2023;24:15835. 10.3390/ijms242115835. 37958821 10.3390/ijms242115835 PMC10648417

[206] hEPO mRNA Product Website. HEPO - Etherna Shop. 2025. https://shop.etherna.be/products/hepo. Accessed 18 Aug 2025

[207] EPO mRNA - Gene Replacement mRNA Product Website. OZ Biosci. 2025. https://ozbiosciences.com/gene-replacement-mrna/128-epo-mrna.html. Accessed 18 Aug 2025

[208] Hou X, Zaks T, Langer R, Dong Y. Lipid nanoparticles for mRNA delivery. Nat Rev Mater. 2021;6:1078–94. 10.1038/s41578-021-00358-0. 34394960 10.1038/s41578-021-00358-0 PMC8353930

[209] Riley RS, Kashyap MV, Billingsley MM, White B, Alameh M-G, Bose SK, et al. Ionizable lipid nanoparticles for in utero mRNA delivery. Sci Adv. 2021;7:eaba1028. 10.1126/sciadv.aba1028. 33523869 10.1126/sciadv.aba1028 PMC7806221

[210] Mortensen SP, Egginton S, Madsen M, Hansen JB, Munch GDW, Iepsen UW, et al. Alpha adrenergic receptor blockade increases capillarization and fractional O2 extraction and lowers blood flow in contracting human skeletal muscle. Acta Physiol. 2017;221:32–43. 10.1111/apha.12857. 10.1111/apha.12857 28199786

[211] Deev R, Plaksa I, Bozo I, Mzhavanadze N, Suchkov I, Chervyakov Y, et al. Results of 5-year follow-up study in patients with peripheral artery disease treated with PL-VEGF165 for intermittent claudication. Ther Adv Cardiovasc Dis. 2018;12:237–46. 10.1177/1753944718786926. 29996720 10.1177/1753944718786926 PMC6116753

[212] Tongers J, Roncalli JG, Losordo DW. Therapeutic angiogenesis for critical limb ischemia. Circulation. 2008;118:9–16. 10.1161/CIRCULATIONAHA.108.784371. 18591450 10.1161/CIRCULATIONAHA.108.784371

[213] Genentech, Inc. A Phase II, Double-Blind, Randomized, Placebo-Controlled Study to Assess the Effect of Topical Recombinant Human Vascular Endothelial Growth Factor (Telbermin) for Induction of Healing of Diabetic Foot Ulcers. clinicaltrials.gov; 2013 Jan. Report No.: NCT00351767. https://clinicaltrials.gov/study/NCT00351767. Accessed 10 Feb 2026

[214] Hanft JR, Pollak RA, Barbul A, van Gils C, Kwon PS, Gray SM, et al. Phase I trial on the safety of topical rhVEGF on chronic neuropathic diabetic foot ulcers. J Wound Care. 2008;17:30–7. 10.12968/jowc.2008.17.1.27917. 18210954 10.12968/jowc.2008.17.1.27917

[215] Henry TD, Annex BH, McKendall GR, Azrin MA, Lopez JJ, Giordano FJ, et al. The VIVA Trial. Circulation. 2003;107:1359–65. 10.1161/01.CIR.0000061911.47710.8A. 12642354 10.1161/01.cir.0000061911.47710.8a

[216] Stewart DJ, Kutryk MJB, Fitchett D, Freeman M, Camack N, Su Y, et al. VEGF gene therapy fails to improve perfusion of ischemic myocardium in patients with advanced coronary disease: results of the NORTHERN trial. Mol Ther J Am Soc Gene Ther. 2009;17:1109–15. 10.1038/mt.2009.70. 10.1038/mt.2009.70 PMC2835194 19352324

[217] Stewart DJ, Hilton JD, Arnold JMO, Gregoire J, Rivard A, Archer SL, et al. Angiogenic gene therapy in patients with nonrevascularizable ischemic heart disease: a phase 2 randomized, controlled trial of AdVEGF121 (AdVEGF121) versus maximum medical treatment. Gene Ther. 2006;13:1503–11. 10.1038/sj.gt.3302802. 16791287 10.1038/sj.gt.3302802

[218] Nakamura K, Henry TD, Traverse JH, Latter DA, Mokadam NA, Answini GA, et al. Angiogenic gene therapy for refractory angina: results of the EXACT Phase 2 trial. Circ Cardiovasc Interv. 2024;17:e014054. 10.1161/CIRCINTERVENTIONS.124.014054. 38696284 10.1161/CIRCINTERVENTIONS.124.014054 PMC11097950

[219] XyloCor Therapeutics, Inc. Endocardial Delivery of XC001 Gene Therapy for Refractory Angina Coronary Treatment: A 26-Week (With 26 Week Extension) Phase 2b Randomized, Multi-Center, Double-Blind, Sham Controlled Study to Evaluate Efficacy and Safety. clinicaltrials.gov; 2025 July. Report No.: NCT07048808. https://clinicaltrials.gov/study/NCT07048808. Accessed 25 Feb 2026

[220] Lampela J, Pajula J, Järveläinen N, Siimes S, Laham-Karam N, Kivelä A, et al. Cardiac vein retroinjections provide an efficient approach for global left ventricular gene transfer with adenovirus and adeno-associated virus. Sci Rep. 2024;14:1467. 10.1038/s41598-024-51712-5. 38233585 10.1038/s41598-024-51712-5 PMC10794695

[221] Henry TD, Grines CL, Watkins MW, Dib N, Barbeau G, Moreadith R, et al. Effects of Ad5FGF-4 in patients with angina: an analysis of pooled data from the AGENT-3 and AGENT-4 trials. J Am Coll Cardiol. 2007;50:1038–46. 10.1016/j.jacc.2007.06.010. 17825712 10.1016/j.jacc.2007.06.010

[222] Yang Z-J, Zhang Y-R, Chen B, Zhang S-L, Jia E-Z, Wang L-S, et al. Phase I clinical trial on intracoronary administration of Ad-hHGF treating severe coronary artery disease. Mol Biol Rep. 2009;36:1323–9. 10.1007/s11033-008-9315-3. 18649012 10.1007/s11033-008-9315-3

[223] Tenchov R, Bird R, Curtze AE, Zhou Q. Lipid nanoparticles─from liposomes to mRNA vaccine delivery, a landscape of research diversity and advancement. ACS Nano. 2021;15:16982–7015. 10.1021/acsnano.1c04996. 34181394 10.1021/acsnano.1c04996

[224] Wolff JA, Malone RW, Williams P, Chong W, Acsadi G, Jani A, et al. Direct gene transfer into mouse muscle in vivo. Science. 1990;247:1465–8. 10.1126/science.1690918. 1690918 10.1126/science.1690918

[225] Wells DJ. Gene doping: the hype and the reality. Br J Pharmacol. 2008;154:623–31. 10.1038/bjp.2008.144. 18500383 10.1038/bjp.2008.144 PMC2439520

[226] Frangoul H, Locatelli F, Sharma A, Bhatia M, Mapara M, Molinari L, et al. Exagamglogene autotemcel for severe sickle cell disease. N Engl J Med. 2024;390:1649–62. 10.1056/NEJMoa2309676. 38661449 10.1056/NEJMoa2309676

[227] Laurent M, Geoffroy M, Pavani G, Guiraud S. CRISPR-based gene therapies: from preclinical to clinical treatments. Cells. 2024;13:800. 10.3390/cells13100800. 38786024 10.3390/cells13100800 PMC11119143

[228] Palmgren G. Overview CRISPR Clinical Trials 2025. CRISPR Med. News. 2025. https://crisprmedicinenews.com/clinical-trials/. Accessed 4 Jul 2025

[229] Zheng Y, Li Y, Zhou K, Li T, VanDusen NJ, Hua Y. Precise genome-editing in human diseases: mechanisms, strategies and applications. Signal Transduct Target Ther. 2024;9:47. 10.1038/s41392-024-01750-2. 38409199 10.1038/s41392-024-01750-2 PMC10897424

[230] Faure G, Saito M, Wilkinson ME, Quinones-Olvera N, Xu P, Flam-Shepherd D, et al. TIGR-Tas: a family of modular RNA-guided DNA-targeting systems in prokaryotes and their viruses. Science. 2025. 10.1126/science.adv9789. 40014690 10.1126/science.adv9789 PMC12045711

[231] Korpela H, Lampela J, Airaksinen J, Järveläinen N, Siimes S, Valli K, et al. AAV2-VEGF-B gene therapy failed to induce angiogenesis in ischemic porcine myocardium due to inflammatory responses. Gene Ther. 2022;29:643–52. 10.1038/s41434-022-00322-9. 35132204 10.1038/s41434-022-00322-9 PMC9684066

[232] Rossi C, Lees M, Mehta V, Heikura T, Martin J, Zachary I, et al. Comparison of efficiency and function of vascular endothelial growth factor adenovirus vectors in endothelial cells for gene therapy of placental insufficiency. Hum Gene Ther. 2020;31:1190–202. 10.1089/hum.2020.006. 32988220 10.1089/hum.2020.006 PMC7698978

[233] Toivanen PI, Nieminen T, Viitanen L, Alitalo A, Roschier M, Jauhiainen S, et al. Novel vascular endothelial growth factor D variants with increased biological activity. J Biol Chem. 2009;284:16037–48. 10.1074/jbc.M109.001123. 19366703 10.1074/jbc.M109.001123 PMC2708897

[234] Klotz L, Norman S, Vieira JM, Masters M, Rohling M, Dubé KN, et al. Cardiac lymphatics are heterogeneous in origin and respond to injury. Nature. 2015;522:62–7. 10.1038/nature14483. 25992544 10.1038/nature14483 PMC4458138

[235] Chen HI, Sharma B, Akerberg BN, Numi HJ, Kivelä R, Saharinen P, et al. The sinus venosus contributes to coronary vasculature through VEGFC-stimulated angiogenesis. Dev Camb Engl. 2014;141:4500–12. 10.1242/dev.113639. 10.1242/dev.113639 PMC4302936 25377552

[236] Shinaoka A, Kimata Y. Lymphatic flow dynamics under exercise load assessed with thoracic duct ultrasonography. Sci Rep. 2025;15:14323. 10.1038/s41598-025-99416-8. 40275043 10.1038/s41598-025-99416-8 PMC12022353

[237] Havas E, Parviainen T, Vuorela J, Toivanen J, Nikula T, Vihko V. Lymph flow dynamics in exercising human skeletal muscle as detected by scintography. J Physiol. 1997;504:233–9. 10.1111/j.1469-7793.1997.233bf.x. 9350633 10.1111/j.1469-7793.1997.233bf.x PMC1159951

[238] Gerber H-P, Ferrara N. The role of VEGF in normal and neoplastic hematopoiesis. J Mol Med. 2003;81:20–31. 10.1007/s00109-002-0397-4. 12545246 10.1007/s00109-002-0397-4

[239] Cerdan C, Rouleau A, Bhatia M. VEGF-A165 augments erythropoietic development from human embryonic stem cells. Blood. 2004;103:2504–12. 10.1182/blood-2003-07-2563. 14656883 10.1182/blood-2003-07-2563

[240] Greenwald AC, Licht T, Kumar S, Oladipupo SS, Iyer S, Grunewald M, et al. VEGF expands erythropoiesis via hypoxia-independent induction of erythropoietin in noncanonical perivascular stromal cells. J Exp Med. 2019;216:215–30. 10.1084/jem.20180752. 30545903 10.1084/jem.20180752 PMC6314526

[241] Rebar EJ. Development of pro-angiogenic engineered transcription factors for the treatment of cardiovascular disease. Expert Opin Investig Drugs. 2004;13:829–39. 10.1517/13543784.13.7.829. 15212621 10.1517/13543784.13.7.829

[242] Pajusola K, Künnapuu J, Vuorikoski S, Soronen J, André H, Pereira T, et al. Stabilized HIF-1α is superior to VEGF for angiogenesis in skeletal muscle via adeno-associated virus gene transfer. FASEB J. 2005. 10.1096/fj.05-3720fje. 15958522 10.1096/fj.05-3720fje

[243] Czibik G, Gravning J, Martinov V, Ishaq B, Knudsen E, Attramadal H, et al. Gene therapy with hypoxia-inducible factor 1 alpha in skeletal muscle is cardioprotective *in vivo*. Life Sci. 2011;88:543–50. 10.1016/j.lfs.2011.01.006. 21238463 10.1016/j.lfs.2011.01.006

[244] Benita Y, Kikuchi H, Smith AD, Zhang MQ, Chung DC, Xavier RJ. An integrative genomics approach identifies hypoxia inducible factor-1 (HIF-1)-target genes that form the core response to hypoxia. Nucleic Acids Res. 2009;37:4587–602. 10.1093/nar/gkp425. 19491311 10.1093/nar/gkp425 PMC2724271

[245] Elson DA, Thurston G, Huang LE, Ginzinger DG, McDonald DM, Johnson RS, et al. Induction of hypervascularity without leakage or inflammation in transgenic mice overexpressing hypoxia-inducible factor-1alpha. Genes Dev. 2001;15:2520–32. 10.1101/gad.914801. 11581158 10.1101/gad.914801 PMC312791

[246] Zhu X, Jiang L, Wei X, Long M, Du Y. Roxadustat: not just for anemia. Front Pharmacol. 2022;13:971795. 10.3389/fphar.2022.971795. 36105189 10.3389/fphar.2022.971795 PMC9465375

[247] Zhu Y, Wang Y, Jia Y, Xu J, Chai Y. Roxadustat promotes angiogenesis through HIF-1α/VEGF/VEGFR2 signaling and accelerates cutaneous wound healing in diabetic rats. Wound Repair Regen. 2019;27:324–34. 10.1111/wrr.12708. 30817065 10.1111/wrr.12708

[248] Wikipedia. Bertrand Moulinet. Wikipedia. 2025. https://en.wikipedia.org/w/index.php?title=Bertrand_Moulinet&oldid=1281486027. Accessed 5 Apr 2025

[249] Checkouri A, Gheddar L, Arbouche N, Raul J-S, Kintz P. Simultaneous detection of three hypoxia-inducible factor stabilizers—molidustat, roxadustat, and vadadustat—in multiple keratinized matrices and its application in a doping context. Drug Test Anal. 2024. 10.1002/dta.3771. 38992954 10.1002/dta.3771 PMC12012410

[250] Janssens LK, De Wilde L, Van Eenoo P, Stove CP. Untargeted detection of HIF stabilizers in doping samples: activity-based screening with a stable in vitro bioassay. Anal Chem. 2024;96:238–47. 10.1021/acs.analchem.3c03816. 38117670 10.1021/acs.analchem.3c03816

[251] Houston RL, Ayoko G, Izake EL. Light-driven biosensor for the rapid and selective detection of hypoxia-inducible factor-prolyl hydroxylase domain inhibitors in aqueous media and saliva. Mikrochim Acta. 2025;192:709. 10.1007/s00604-025-07579-y. 41034642 10.1007/s00604-025-07579-y PMC12488750

[252] Yazdani M. Technical aspects of oxygen level regulation in primary cell cultures: a review. Interdiscip Toxicol. 2016;9:85–9. 10.1515/intox-2016-0011. 28652851 10.1515/intox-2016-0011 PMC5464680

[253] Beuck S, Schänzer W, Thevis M. Hypoxia-inducible factor stabilizers and other small-molecule erythropoiesis-stimulating agents in current and preventive doping analysis. Drug Test Anal. 2012;4:830–45. 10.1002/dta.390. 22362605 10.1002/dta.390

[254] Jelkmann W. Xenon misuse in sports – increase of hypoxia-inducible factors and erythropoietin, or nothing but „hot air“? Dtsch Z Sportmed. 2014;65:267–71. 10.5960/dzsm.2014.143.

[255] Postnikov PV, Pronina IV, Polyansky DS, Efimova YA, Ordzhonikidze ZG, Badtieva VA, et al. Analysis of specific microRNAs expressed after intake of cobalt and xenon preparations for anti-doping control. Bull Exp Biol Med. 2025;178:726–32. 10.1007/s10517-025-06406-x. 40442471 10.1007/s10517-025-06406-x

[256] Fukumura D, Gohongi T, Kadambi A, Izumi Y, Ang J, Yun C-O, et al. Predominant role of endothelial nitric oxide synthase in vascular endothelial growth factor-induced angiogenesis and vascular permeability. Proc Natl Acad Sci U S A. 2001;98:2604–9. 10.1073/pnas.041359198. 11226286 10.1073/pnas.041359198 PMC30185

[257] Sessa WC. Molecular control of blood flow and angiogenesis: role of nitric oxide. J Thromb Haemost. 2009;7:35–7. 10.1111/j.1538-7836.2009.03424.x. 19630764 10.1111/j.1538-7836.2009.03424.x

[258] Motterlini R, Otterbein LE. The therapeutic potential of carbon monoxide. Nat Rev Drug Discov. 2010;9:728–43. 10.1038/nrd3228. 20811383 10.1038/nrd3228

[259] Ling K, Men F, Wang W-C, Zhou Y-Q, Zhang H-W, Ye D-W. Carbon monoxide and its controlled release: therapeutic application, detection, and development of carbon monoxide releasing molecules (CORMs). J Med Chem. 2018;61:2611–35. 10.1021/acs.jmedchem.6b01153. 28876065 10.1021/acs.jmedchem.6b01153

[260] Hopper CP, Zambrana PN, Goebel U, Wollborn J. A brief history of carbon monoxide and its therapeutic origins. Nitric Oxide. 2021;111–112:45–63. 10.1016/j.niox.2021.04.001. 33838343 10.1016/j.niox.2021.04.001

[261] Yang X, de Caestecker M, Otterbein LE, Wang B. Carbon monoxide: an emerging therapy for acute kidney injury. Med Res Rev. 2020;40:1147–77. 10.1002/med.21650. 31820474 10.1002/med.21650 PMC7280078

[262] Dulak J, Józkowicz A, Foresti R, Kasza A, Frick M, Huk I, et al. Heme oxygenase activity modulates vascular endothelial growth factor synthesis in vascular smooth muscle cells. Antioxid Redox Signal. 2002;4:229–40. 10.1089/152308602753666280. 12006174 10.1089/152308602753666280

[263] Daghman NA, McHale CM, Savage GM, Price S, Winter PC, Maxwell AP, et al. Regulation of erythropoietin gene expression depends on two different oxygen-sensing mechanisms. Mol Genet Metab. 1999;67:113–7. 10.1006/mgme.1999.2851. 10356310 10.1006/mgme.1999.2851

[264] Gatterer H, Dünnwald T, Woyke S, Faulhaber M, Schumacher YO, Schobersberger W. Low-dose carbon monoxide inhalation to increase total hemoglobin mass and endurance performance: scientific evidence and implications. Front Physiol. 2024. 10.3389/fphys.2024.1490205. 39473608 10.3389/fphys.2024.1490205 PMC11519625

[265] Bansal S, Liu D, Mao Q, Bauer N, Wang B. Carbon monoxide as a potential therapeutic agent: a molecular analysis of its safety profiles. J Med Chem. 2024;67:9789–815. 10.1021/acs.jmedchem.4c00823. 38864348 10.1021/acs.jmedchem.4c00823 PMC11215727

[266] Davis AC, Boburg S, Jr RO. Little-known makers of generic drugs played central role in opioid crisis, records show. Wash Post. 2019; https://www.washingtonpost.com/investigations/little-known-generic-drug-companies-played-central-role-in-opioid-crisis-documents-reveal/2019/07/26/95e08b46-ac5c-11e9-a0c9-6d2d7818f3da_story.html. Accessed 20 Apr 2025

[267] Siebenmann C, Keiser S, Robach P, Lundby C. CORP: the assessment of total hemoglobin mass by carbon monoxide rebreathing. J Appl Physiol. 2017;123:645–54. 10.1152/japplphysiol.00185.2017. 28663373 10.1152/japplphysiol.00185.2017

[268] Lundby C, Pilegaard H, Andersen JL, van Hall G, Sander M, Calbet JAL. Acclimatization to 4100 m does not change capillary density or mRNA expression of potential angiogenesis regulatory factors in human skeletal muscle. J Exp Biol. 2004;207:3865–71. 10.1242/jeb.01225. 15472017 10.1242/jeb.01225

[269] Peacock AJ. Oxygen at high altitude. BMJ Brit Med J. 1998;317:1063–6. 10.1136/bmj.317.7165.1063. 9774298 10.1136/bmj.317.7165.1063 PMC1114067

[270] Wagner PD. Muscle intracellular oxygenation during exercise: optimization for oxygen transport, metabolism, and adaptive change. Eur J Appl Physiol. 2012;112:1–8. 10.1007/s00421-011-1955-7. 21512800 10.1007/s00421-011-1955-7

[271] Richardson RS, Duteil S, Wary C, Wray DW, Hoff J, Carlier PG. Human skeletal muscle intracellular oxygenation: the impact of ambient oxygen availability. J Physiol. 2006;571:415–24. 10.1113/jphysiol.2005.102327. 16396926 10.1113/jphysiol.2005.102327 PMC1796788

[272] Richardson RS, Newcomer SC, Noyszewski EA. Skeletal muscle intracellular PO2 assessed by myoglobin desaturation: response to graded exercise. J Appl Physiol. 2001;91:2679–85. 10.1152/jappl.2001.91.6.2679. 11717234 10.1152/jappl.2001.91.6.2679

[273] Gayeski TE, Connett RJ, Honig CR. Minimum intracellular PO2 for maximum cytochrome turnover in red muscle in situ. Am J Physiol Heart Circ Physiol. 1987;252:H906–15. 10.1152/ajpheart.1987.252.5.H906. 10.1152/ajpheart.1987.252.5.H906 3578540

[274] Deveci D, Marshall JM, Egginton S. Relationship between capillary angiogenesis, fiber type, and fiber size in chronic systemic hypoxia. Am J Physiol Heart Circ Physiol. 2001;281:H241–52. 10.1152/ajpheart.2001.281.1.H241. 11406491 10.1152/ajpheart.2001.281.1.H241

[275] Deveci D, Marshall JM, Egginton S. Chronic hypoxia induces prolonged angiogenesis in skeletal muscles of rat. Exp Physiol. 2002;87:287–91. 10.1113/eph8702377. 12089595 10.1113/eph8702377

[276] Breen E, Tang K, Olfert M, Knapp A, Wagner P. Skeletal muscle capillarity during hypoxia: VEGF and its activation. High Alt Med Biol. 2008;9:158–66. 10.1089/ham.2008.1010. 18578647 10.1089/ham.2008.1010

[277] Milkiewicz M, Hudlicka O, Shiner R, Egginton S, Brown MD. Vascular endothelial growth factor mRNA and protein do not change in parallel during non-inflammatory skeletal muscle ischaemia in rat. J Physiol. 2006;577:671–8. 10.1113/jphysiol.2006.113357. 16990404 10.1113/jphysiol.2006.113357 PMC1890445

[278] Eichner ER. Sports anemia, iron supplements, and blood doping. Med Sci Sports Exerc. 1992;24:S315-318. 1406203

[279] Weight LM, Klein M, Noakes TD, Jacobs P. ‘Sports anemia’ - a real or apparent phenomenon in endurance-trained athletes? Int J Sports Med. 2008;13:344–7. 10.1055/s-2007-1021278. 10.1055/s-2007-1021278 1521949

[280] Oberholzer L, Lundby C. Hematological adaptations to endurance training. Proc Physiol Soc. 2022;49: SA04. https://www.physoc.org/abstracts/hematological-adaptations-to-endurance-training/.

[281] Gillen CM, Lee R, Mack GW, Tomaselli CM, Nishiyasu T, Nadel ER. Plasma volume expansion in humans after a single intense exercise protocol. J Appl Physiol. 1991;71:1914–20. 10.1152/jappl.1991.71.5.1914. 1761491 10.1152/jappl.1991.71.5.1914

[282] Hagberg JM, Goldberg AP, Lakatta L, O’Connor FC, Becker LC, Lakatta EG, et al. Expanded blood volumes contribute to the increased cardiovascular performance of endurance-trained older men. J Appl Physiol. 1998;85:484–9. 10.1152/jappl.1998.85.2.484. 9688724 10.1152/jappl.1998.85.2.484

[283] Schuler B, Arras M, Keller S, Rettich A, Lundby C, Vogel J, et al. Optimal hematocrit for maximal exercise performance in acute and chronic erythropoietin-treated mice. Proc Natl Acad Sci U S A. 2010;107:419–23. 10.1073/pnas.0912924107. 19966291 10.1073/pnas.0912924107 PMC2806754

[284] Pereira FG, Greenfield AM, Kuennen M, Gillum TL. Exercise induced plasma volume expansion lowers cardiovascular strain during 15-km cycling time-trial in acute normobaric hypoxia. PLoS ONE. 2024;19:e0297553. 10.1371/journal.pone.0297553. 38306343 10.1371/journal.pone.0297553 PMC10836693

[285] Fellmann N. Hormonal and plasma volume alterations following endurance exercise. Sports Med. 1992;13:37–49. 10.2165/00007256-199213010-00004. 1553454 10.2165/00007256-199213010-00004

[286] Convertino VA. Blood volume response to physical activity and inactivity. Am J Med Sci. 2007;334:72–9. 10.1097/MAJ.0b013e318063c6e4. 17630597 10.1097/MAJ.0b013e318063c6e4

[287] Mairbäurl H. Red blood cells in sports: Effects of exercise and training on oxygen supply by red blood cells. Front Physiol. 2013. 10.3389/fphys.2013.00332. 24273518 10.3389/fphys.2013.00332 PMC3824146

[288] Telford RD, Sly GJ, Hahn AG, Cunningham RB, Bryant C, Smith JA. Footstrike is the major cause of hemolysis during running. J Appl Physiol. 2003;94(1):38–42. 10.1152/japplphysiol.00631.2001. 12391035 10.1152/japplphysiol.00631.2001

[289] Green HJ, Carter S, Grant S, Tupling R, Coates G, Ali M. Vascular volumes and hematology in male and female runners and cyclists. Eur J Appl Physiol. 1999;79:244–50. 10.1007/s004210050502. 10.1007/s004210050502 10048629

[290] Miller WL. Blood volume expansion: Can an adaptation of endurance training, altitude acclimatization, and pregnancy inform volume homeostasis in chronic heart failure and why does it matter? A viewpoint. Circ Heart Fail. 2025. 10.1161/CIRCHEARTFAILURE.125.013429. 41439309 10.1161/CIRCHEARTFAILURE.125.013429

[291] Ay C, Reinisch A. Gene therapy: principles, challenges and use in clinical practice. Wien Klin Wochenschr. 2024. 10.1007/s00508-024-02368-8. 38713227 10.1007/s00508-024-02368-8 PMC12081535

[292] Lopes R, Prasad MK. Beyond the promise: evaluating and mitigating off-target effects in CRISPR gene editing for safer therapeutics. Front Bioeng Biotechnol. 2024;11:1339189. 10.3389/fbioe.2023.1339189. 38390600 10.3389/fbioe.2023.1339189 PMC10883050

[293] Shchaslyvyi AY, Antonenko SV, Tesliuk MG, Telegeev GD. Current state of human gene therapy: approved products and vectors. Pharmaceuticals Basel. 2023;16:1416. 10.3390/ph16101416. 37895887 10.3390/ph16101416 PMC10609992

[294] O’Hara AJ, Howell JM, Taplin RH, Fletcher S, Lloyd F, Kakulas B, et al. The spread of transgene expression at the site of gene construct injection. Muscle Nerve. 2001;24:488–95. 10.1002/mus.1031. 11268020 10.1002/mus.1031

[295] Song E, Mao T, Dong H, Boisserand LSB, Antila S, Bosenberg M, et al. VEGF-C-driven lymphatic drainage enables immunosurveillance of brain tumours. Nature. 2020;577:689–94. 10.1038/s41586-019-1912-x. 31942068 10.1038/s41586-019-1912-x PMC7100608

[296] Fernandez-Rodriguez R, Cuadrado-Castano S, Edwards A, Rogic A, Bykov Y, Mena I, et al. Lymphangiogenesis driven by VEGF-C reshapes tumor immune landscape and enables tumor eradication by viral immunotherapy. bioRxiv; 2025. p. 2025.08.27.672466. 10.1101/2025.08.27.672466

[297] Koch AE, Harlow LA, Haines GK, Amento EP, Unemori EN, Wong WL, et al. Vascular endothelial growth factor. A cytokine modulating endothelial function in rheumatoid arthritis. J Immunol. 1994;152:4149–56. 10.4049/jimmunol.152.8.4149. 7511670

[298] Duffy AM, Bouchier-Hayes DJ, Harmey J. Vascular Endothelial Growth Factor (VEGF) and its role in non-endothelial cells: autocrine signalling by VEGF. Madame Curie Biosci Database. Austin (TX): Landes Bioscience; 2013.

[299] Barry P. Finding the golden genes: advances in gene therapy could tempt some athletes to enhance their genetic makeup, leading some researchers to work on detection methods just in case. Sci News. 2008;174:16–21. 10.1002/scin.2008.5591740321.

[300] Cantelmo RA, Da Silva AP, Mendes‐Junior CT, Dorta DJ. Gene doping: present and future. Eur J Sport Sci. 2020;20:1093–101. 10.1080/17461391.2019.1695952. 31787029 10.1080/17461391.2019.1695952

[301] de Boer EN, van der Wouden PE, Johansson LF, van Diemen CC, Haisma HJ. A next-generation sequencing method for gene doping detection that distinguishes low levels of plasmid DNA against a background of genomic DNA. Gene Ther. 2019;26:338–46. 10.1038/s41434-019-0091-6. 31296934 10.1038/s41434-019-0091-6 PMC6760532

[302] Yi J-Y, Choi H, Kim M, Jeong Y, Hahn J-S, Son B, et al. High-throughput multiplexed gene and cell doping analysis through CRISPR-Cas12a system integrated with blood direct PCR. Sci Adv. 2025;11:eadv7234. 10.1126/sciadv.adv7234. 40632864 10.1126/sciadv.adv7234 PMC12239944

[303] Beiter T, Zimmermann M, Fragasso A, Armeanu S, Lauer UM, Bitzer M, et al. Establishing a novel single-copy primer-internal intron-spanning PCR (spiPCR) procedure for the direct detection of gene doping. Exerc Immunol Rev. 2008;14:73–85. https://www.scopus.com/inward/record.uri?eid=2-s2.0-62849115532&partnerID=40&md5=1609faa7fe4350c9febf96df388811fb 19203085

[304] Beiter T, Zimmermann M, Fragasso A, Hudemann J, Niess AM, Bitzer M, et al. Direct and long-term detection of gene doping in conventional blood samples. Gene Ther. 2011;18:225–31. 10.1038/gt.2010.122. 20811468 10.1038/gt.2010.122

[305] Naumann N, Do C, Vollmert C, Krajina M, Thomas A, Cheung HW, et al. Multiplex detection of seven transgenes for human gene doping analysis. Sci Rep. 2025. 10.1038/s41598-025-06677-4. 40542126 10.1038/s41598-025-06677-4 PMC12181312

[306] Rauniyar K, Bokharaie H, Jeltsch M. Expansion and collapse of VEGF diversity in major clades of the animal kingdom. Angiogenesis. 2023;26:437–61. 10.1007/s10456-023-09874-9. 37017884 10.1007/s10456-023-09874-9 PMC10328876

[307] Olofsson B, Pajusola K, von Euler G, Chilov D, Alitalo K, Eriksson U. Genomic organization of the mouse and human genes for vascular endothelial growth factor B (VEGF-B) and characterization of a second splice isoform. J Biol Chem. 1996;271:19310–7. 10.1074/jbc.271.32.19310. 8702615 10.1074/jbc.271.32.19310

[308] Cheshenko N, Krougliak N, Eisensmith RC, Krougliak VA. A novel system for the production of fully deleted adenovirus vectors that does not require helper adenovirus. Gene Ther. 2001;8:846–54. 10.1038/sj.gt.3301459. 11423932 10.1038/sj.gt.3301459

[309] Venter JC, Adams MD, Myers EW, Li PW, Mural RJ, Sutton GG, et al. The sequence of the human genome. Science. 2001;291:1304–51. 10.1126/science.1058040. 11181995 10.1126/science.1058040

[310] Scherer S. Gene Structure. Guide Hum Genome. Cold Spring Harbor Laboratory Press; 2010. https://www.cshlp.org/ghg5_all/section/gene.shtml. Accessed 19 Aug 2025

[311] Rohrmann GF. DNA replication and genome processing. Baculovirus Mol Biol Internet 4th Ed. National Center for Biotechnology Information (US); 2019. https://www.ncbi.nlm.nih.gov/books/NBK543453/. Accessed 19 Aug 2025

[312] R&D Systems. Human VEGF Quantikine ELISA Kit. https://www.rndsystems.com/products/human-vegf-quantikine-elisa-kit_dve00. Accessed 14 May 2025

[313] Będkowska GE, Gacuta E, Zbucka-Krętowska M, Ławicki P, Szmitkowski M, Lemancewicz A, et al. Plasma levels and diagnostic utility of VEGF in a three-year follow-up of patients with breast cancer. J Clin Med. 2021;10:5452. 10.3390/jcm10225452. 34830735 10.3390/jcm10225452 PMC8624052

[314] Hanefeld M, Engelmann K, Appelt D, Sandner D, Weigmann I, Ganz X, et al. Intra-individual variability and circadian rhythm of vascular endothelial growth factors in subjects with normal glucose tolerance and type 2 diabetes. PLoS ONE. 2017;12:e0184234. 10.1371/journal.pone.0184234. 28991900 10.1371/journal.pone.0184234 PMC5633167

[315] Nakamizo T, Cologne J, Kishi T, Takahashi T, Inoue M, Ryukaku H, et al. Reliability, stability during long-term storage, and intra-individual variation of circulating levels of osteopontin, osteoprotegerin, vascular endothelial growth factor-A, and interleukin-17A. Eur J Med Res. 2024;29:133. 10.1186/s40001-024-01722-w. 38368424 10.1186/s40001-024-01722-w PMC10873926

[316] Stefanini MO, Wu FTH, Gabhann FM, Popel AS. The presence of VEGF receptors on the luminal surface of endothelial cells affects VEGF distribution and VEGF signaling. PLoS Comput Biol. 2009;5:e1000622. 10.1371/journal.pcbi.1000622. 20041209 10.1371/journal.pcbi.1000622 PMC2790341

[317] Dahl SL, Bapst AM, Khodo SN, Scholz CC, Wenger RH. Fount, fate, features, and function of renal erythropoietin-producing cells. Pflugers Arch Eur J Physiol. 2022;474:783–97. 10.1007/s00424-022-02714-7. 35750861 10.1007/s00424-022-02714-7 PMC9338912

[318] Ruhrberg C, Gerhardt H, Golding M, Watson R, Ioannidou S, Fujisawa H, et al. Spatially restricted patterning cues provided by heparin-binding VEGF-A control blood vessel branching morphogenesis. Genes Dev. 2002;16:2684–98. 10.1101/gad.242002. 12381667 10.1101/gad.242002 PMC187458

[319] Gerhardt H, Golding M, Fruttiger M, Ruhrberg C, Lundkvist A, Abramsson A, et al. VEGF guides angiogenic sprouting utilizing endothelial tip cell filopodia. J Cell Biol. 2003;161:1163–77. 10.1083/jcb.200302047. 12810700 10.1083/jcb.200302047 PMC2172999

[320] Iljukov S, Kauppi J-P, Uusitalo ALT, Peltonen JE, Schumacher YO. Association between implementation of the athlete biological passport and female elite runners’ performance. Int J Sports Physiol Perform. 2020;15:1231–6. 10.1123/ijspp.2019-0643. 32084627 10.1123/ijspp.2019-0643

[321] Equey T, Pastor A, de la Torre FR, Thomas A, Giraud S, Thevis M, et al. Application of the athlete biological passport approach to the detection of growth hormone doping. J Clin Endocrinol Metab. 2022;107:649–59. 10.1210/clinem/dgab799. 34726230 10.1210/clinem/dgab799

[322] Guerraty M, Bhargava A, Senarathna J, Mendelson AA, Pathak AP. Advances in translational imaging of the microcirculation. Microcirc N Y N 1994. 2021;28:e12683. 10.1111/micc.12683. 10.1111/micc.12683 PMC8647298 33524206

[323] Marchand A, Roulland I, Semence F, Schröder K, Domergue V, Audran M. Detection of hypoxia-regulated microRNAs in blood as potential biomarkers of HIF stabilizer Molidustat. MicroRNA. 2019;8:189–97. 10.2174/2211536608666190117170317. 30657053 10.2174/2211536608666190117170317

[324] Hua Z, Lv Q, Ye W, Wong C-KA, Cai G, Gu D, et al. MiRNA-Directed Regulation of VEGF and Other Angiogenic Factors under Hypoxia. Valcarcel J, editor. PLoS One. 2006;1:e116. 10.1371/journal.pone.0000116 10.1371/journal.pone.0000116 PMC1762435 17205120

[325] Dragčević D, Pandžić Jakšić V, Jakšić O. Athlete biological passport: longitudinal biomarkers and statistics in the fight against doping. Arch Ind Hyg Toxicol. 2024;75:24–31. 10.2478/aiht-2024-75-3793. 10.2478/aiht-2024-75-3793 PMC10978099 38548376

[326] de la Chapelle A, Träskelin AL, Juvonen E. Truncated erythropoietin receptor causes dominantly inherited benign human erythrocytosis. Proc Natl Acad Sci U S A. 1993;90:4495–9. 8506290 10.1073/pnas.90.10.4495 PMC46538

[327] Luna SE, Camarena J, Hampton JP, Majeti KR, Charlesworth CT, Soupene E, et al. Enhancement of erythropoietic output by Cas9-mediated insertion of a natural variant in haematopoietic stem and progenitor cells. Nat Biomed Eng. 2024;8:1540–52. 10.1038/s41551-024-01222-6. 38886504 10.1038/s41551-024-01222-6 PMC11668683

[328] Manders F, van Boxtel R, Middelkamp S. The dynamics of somatic mutagenesis during life in humans. Front Aging. 2021;2:802407. 10.3389/fragi.2021.802407. 35822044 10.3389/fragi.2021.802407 PMC9261377

[329] Denduluri N, Yang SX, Berman AW, Nguyen D, Liewehr DJ, Steinberg SM, et al. Circulating biomarkers of bevacizumab activity in patients with breast cancer. Cancer Biol Ther. 2008;7:15–20. 10.4161/cbt.7.1.5337. 18059178 10.4161/cbt.7.1.5337

[330] Hegde PS, Jubb AM, Chen D, Li NF, Meng YG, Bernaards C, et al. Predictive impact of circulating vascular endothelial growth factor in four phase III trials evaluating bevacizumab. Clin Cancer Res. 2013;19:929–37. 10.1158/1078-0432.CCR-12-2535. 23169435 10.1158/1078-0432.CCR-12-2535

[331] Tanaka K, Sugisaka J, Shiraishi Y, Watanabe Y, Daga H, Azuma K, et al. Serum VEGF-A as a biomarker for the addition of bevacizumab to chemo-immunotherapy in metastatic NSCLC. Nat Commun. 2025;16:2825. 10.1038/s41467-025-58186-7. 40121197 10.1038/s41467-025-58186-7 PMC11929838

[332] Souma T, Suzuki N, Yamamoto M. Renal erythropoietin-producing cells in health and disease. Front Physiol. 2015. 10.3389/fphys.2015.00167. 26089800 10.3389/fphys.2015.00167 PMC4452800

[333] Roy S, Driggs J, Elgharably H, Biswas S, Findley M, Khanna S, et al. Platelet rich fibrin matrix improves wound angiogenesis via inducing endothelial cell proliferation. Wound Repair Regen. 2011;19:753–66. 10.1111/j.1524-475X.2011.00740.x. 22092846 10.1111/j.1524-475X.2011.00740.x PMC3623798

[334] Mineur P, Colige AC, Deroanne ChristopheF, Dubail J, Kesteloot F, Habraken Y, et al. Newly identified biologically active and proteolysis-resistant VEGF-A isoform VEGF111 is induced by genotoxic agents. J Cell Biol. 2007;179:1261–73. 10.1083/jcb.200703052. 18086921 10.1083/jcb.200703052 PMC2140032

[335] Abedini A, Klötzer KA, Susztak K. Unmasking the elusive erythropoietin-producing ‘Norn’ cell. Nat Med. 2023;29:1064–5. 10.1038/s41591-023-02322-7. 37130964 10.1038/s41591-023-02322-7 PMC11611266

[336] Carmeliet P, Ng Y-S, Nuyens D, Theilmeier G, Brusselmans K, Cornelissen I, et al. Impaired myocardial angiogenesis and ischemic cardiomyopathy in mice lacking the vascular endothelial growth factor isoforms VEGF 164 and VEGF 188. Nat Med. 1999;5:495–502. 10.1038/8379. 10229225 10.1038/8379

[337] Woolard J, Bevan HS, Harper SJ, Bates DO. Molecular diversity of VEGF-A as a regulator of its biological activity. Microcirculation. 2009;16:572–92. 10.1080/10739680902997333. 19521900 10.1080/10739680902997333 PMC2929464

[338] Nakayama M, Nakayama A, van Lessen M, Yamamoto H, Hoffmann S, Drexler HCA, et al. Spatial regulation of VEGF receptor endocytosis in angiogenesis. Nat Cell Biol. 2013;15:249–60. 10.1038/ncb2679. 23354168 10.1038/ncb2679 PMC3901019

[339] Dragoni S, Turowski P. Polarised VEGFA signalling at vascular blood–neural barriers. Int J Mol Sci. 2018;19:1378. 10.3390/ijms19051378. 29734754 10.3390/ijms19051378 PMC5983809

[340] Patel SA, Nilsson MB, Le X, Cascone T, Jain RK, Heymach JV. Molecular mechanisms and future implications of VEGF/VEGFR in cancer therapy. Clin Cancer Res. 2023;29:30–9. 10.1158/1078-0432.CCR-22-1366. 35969170 10.1158/1078-0432.CCR-22-1366 PMC10274152

[341] Gyanchandani R, Kim S. Predictive biomarkers to anti-VEGF therapy: progress towards an elusive goal. Clin Cancer Res. 2013;19:755–7. 10.1158/1078-0432.CCR-12-3585. 23386694 10.1158/1078-0432.CCR-12-3585 PMC3580199

[342] Zhao W, Jiang J. Advances in predictive biomarkers for anti-angiogenic therapy in non-small cell lung cancer. Cancer Control. 2024;31:10732748241270589. 10.1177/10732748241270589. 39192835 10.1177/10732748241270589 PMC11363049

[343] Hudson N, Powner MB, Sarker MH, Burgoyne T, Campbell M, Ockrim ZK, et al. Differential Apicobasal VEGF signaling at vascular blood-neural barriers. Dev Cell. 2014;30:541–52. 10.1016/j.devcel.2014.06.027. 25175707 10.1016/j.devcel.2014.06.027 PMC4160345

[344] Jain RK, Munn LL. Leaky vessels? Call Ang1! Nat Med. 2000;6:131–2. 10.1038/72212. 10655092 10.1038/72212

[345] Gavard J, Patel V, Gutkind JS. Angiopoietin-1 prevents VEGF-induced endothelial permeability by sequestering Src through mDia. Dev Cell. 2008;14:25–36. 10.1016/j.devcel.2007.10.019. 18194650 10.1016/j.devcel.2007.10.019

[346] Yang R, Ogasawara AK, Zioncheck TF, Ren Z, He G-W, DeGuzman GG, et al. Exaggerated hypotensive effect of Vascular Endothelial Growth Factor in Spontaneously Hypertensive Rats. Hypertension. 2002;39:815–20. 10.1161/hy0302.105398. 11897770 10.1161/hy0302.105398

[347] Waller JP, Burke SP, Engel J, Chade AR, Bidwell GL. A dose-escalating toxicology study of the candidate biologic ELP-VEGF. Sci Rep. 2021;11:6216. 10.1038/s41598-021-85693-6. 33737643 10.1038/s41598-021-85693-6 PMC7973730

[348] Kataru RP, Baik JE, Park HJ, Ly CL, Shin J, Schwartz N, et al. Lymphatic-specific intracellular modulation of receptor tyrosine kinase signaling improves lymphatic growth and function. Sci Signal. 2021; 10.1126/scisignal.abc0836 PMC8567054 34376570

[349] Spinelli L, Lindsay YE, Leslie NR. PTEN inhibitors: an evaluation of current compounds. Adv Biol Regul. 2015;57:102–11. 10.1016/j.jbior.2014.09.012. 25446882 10.1016/j.jbior.2014.09.012

[350] Tomuleasa C, Tigu A-B, Munteanu R, Moldovan C-S, Kegyes D, Onaciu A, et al. Therapeutic advances of targeting receptor tyrosine kinases in cancer. Signal Transduct Target Ther. 2024;9:201. 10.1038/s41392-024-01899-w. 39138146 10.1038/s41392-024-01899-w PMC11323831

[351] Hayashi M, Majumdar A, Li X, Adler J, Sun Z, Vertuani S, et al. VE-PTP regulates VEGFR2 activity in stalk cells to establish endothelial cell polarity and lumen formation. Nat Commun. 2013;4:1672. 10.1038/ncomms2683. 23575676 10.1038/ncomms2683 PMC3644080

[352] Lanahan AA, Lech D, Dubrac A, Zhang J, Zhuang ZW, Eichmann A, et al. PTP1b is a physiologic regulator of Vascular Endothelial Growth Factor signaling in endothelial cells. Circulation. 2014;130:902–9. 10.1161/CIRCULATIONAHA.114.009683. 24982127 10.1161/CIRCULATIONAHA.114.009683 PMC6060619

[353] Liu R, Mathieu C, Berthelet J, Zhang W, Dupret J-M, Rodrigues Lima F. Human Protein Tyrosine Phosphatase 1B (PTP1B): from structure to clinical inhibitor perspectives. Int J Mol Sci. 2022;23:7027. 10.3390/ijms23137027. 35806030 10.3390/ijms23137027 PMC9266911

[354] Shen J, Frye M, Lee BL, Reinardy JL, McClung JM, Ding K, et al. Targeting VE-PTP activates TIE2 and stabilizes the ocular vasculature. J Clin Invest. 2014;124:4564–76. 10.1172/JCI74527. 25180601 10.1172/JCI74527 PMC4191011

[355] Schmidt WFJ, Hoffmeister T, Haupt S, Schwenke D, Wachsmuth NB, Byrnes WC. Chronic exposure to low-dose Carbon Monoxide alters hemoglobin mass and V˙O2max. Med Sci Sports Exerc. 2020;52:1879. 10.1249/MSS.0000000000002330. 32118696 10.1249/MSS.0000000000002330

[356] Xu J, Ding X, Fu Y, Meng Q, Wang L, Zhang M, et al. Discovery of novel and potent Prolyl Hydroxylase Domain-Containing Protein (PHD) inhibitors for the treatment of anemia. J Med Chem. 2024;67:1393–405. 10.1021/acs.jmedchem.3c01932. 38189253 10.1021/acs.jmedchem.3c01932

[357] Mortimer EAJ, Monson RR, MacMahon B. Reduction in mortality from coronary heart disease in men residing at high altitude. N Engl J Med. 1977;296:581–5. 10.1056/NEJM197703172961101. 840241 10.1056/NEJM197703172961101

[358] Neckár J, Szárszoi O, Koten L, Papousek F, Ost’ádal B, Grover GJ, et al. Effects of mitochondrial K(ATP) modulators on cardioprotection induced by chronic high altitude hypoxia in rats. Cardiovasc Res. 2002;55:567–75. 10.1016/s0008-6363(02)00456-x. 12160954 10.1016/s0008-6363(02)00456-x

[359] Ke J, Wang L, Xiao D. Cardiovascular adaptation to high-altitude hypoxia. Hypoxia Hum Dis. 2017. 10.5772/65354.

[360] Stembridge M, Williams AM, Gasho C, Dawkins TG, Drane A, Villafuerte FC, et al. The overlooked significance of plasma volume for successful adaptation to high altitude in Sherpa and Andean natives. Proc Natl Acad Sci U S A. 2019;116:16177–9. 10.1073/pnas.1909002116. 31358634 10.1073/pnas.1909002116 PMC6697886

[361] Pinckard K, Baskin KK, Stanford KI. Effects of exercise to improve cardiovascular health. Front Cardiovasc Med. 2019. 10.3389/fcvm.2019.00069. 31214598 10.3389/fcvm.2019.00069 PMC6557987

[362] Ulrich R, Pope HG, Cléret L, Petróczi A, Nepusz T, Schaffer J, et al. Doping in two elite athletics competitions assessed by randomized-response surveys. Sports Med. 2018;48:211–9. 10.1007/s40279-017-0765-4. 28849386 10.1007/s40279-017-0765-4

