# Supplementary material for: Angiogenic Doping: Plausible Yet Difficult to Detect
Source: Sports Med. 2026 May 21;56(9):2089–119. doi: 10.1007/s40279-026-02447-y (PMC13499858; doi:10.1007/s40279-026-02447-y)
Supplement: Supplementary file 1 [file 40279_2026_2447_MOESM1_ESM.pdf]

*Sports Medicine:* Supplementary Material

# Angiogenic Doping: Plausible Yet Difficult to Detect

Sofie Lehto, Setareh Sima, Jaana Künnapuu, Sergei Iljukov[1], Michael Jeltsch[2]

[1]Terveystalo Lahti, Finland, sergei.iljukov@gmail.com

[2]Drug Research Program, University of Helsinki, Finland, michael@jeltsch.org

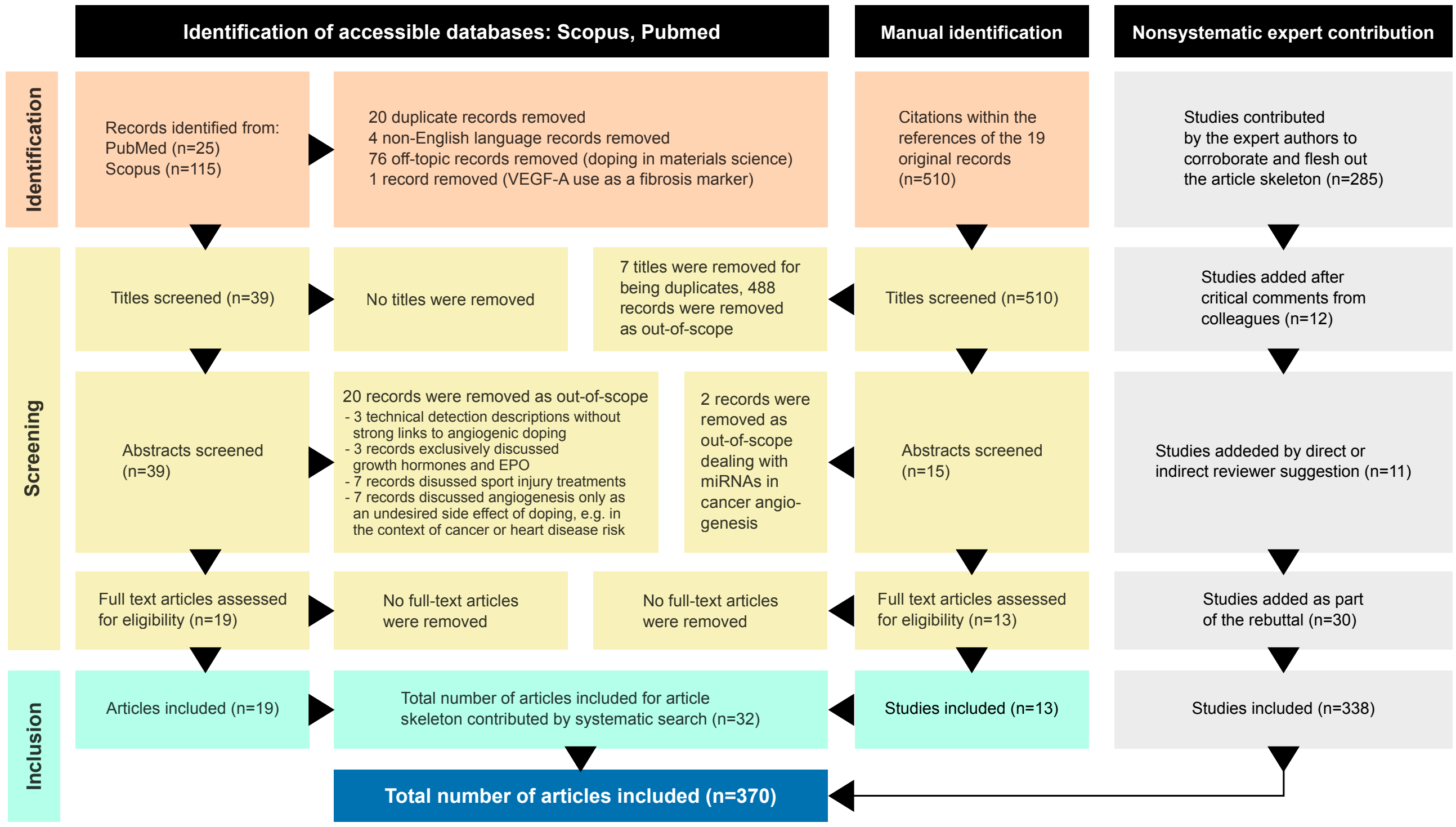


**Figure S1. Literature search diagram.** The major issue during the literature search was the term doping, which has two completely unrelated meanings in science: doping in sports and doping in materials science. When limiting the keyword search to doping in sports, we were missing records, but when we used the unqualified keyword search doping, the results predominantly referred to doping in materials science. Very few articles explicitly combine sports doping with angiogenesis/VEGF keywords, which strongly suggests that angiogenic doping is an underexplored niche within the gene doping literature.
Additionally, the low overlap between the Scopus and PubMed searches indicates that most reviews must cite non-doping research to explore the topic. This was also the case for this review. After assembling the skeleton of the review based on the systematic literature search, the subsequently cited research (and the majority of the total references) was contributed by the authors in their respective areas of expertise.

Scopus search term: (KEY(doping) AND TITLE-ABS-KEY(angiogenesis) OR TITLE-ABS-KEY(vegf) OR TITLE-ABS-KEY(vascular endothelial growth factor) OR TITLE-ABS-KEY(vegfa)).

PubMed search term: (((doping[Title/Abstract]) AND (sports[Title/Abstract])) or (doping in sports[MeSH Terms]) or (blood doping[MeSH Terms]) AND ((angiogenesis[MeSH Terms]) OR (angiogenesis[Text Word]) OR (vegf[Text Word]) OR (vascular endothelial growth factor[Text Word]) OR (vascular endothelial growth factor-a[Text Word])).
